# Colitis ameliorates cholestatic liver disease via suppression of bile acid synthesis

**DOI:** 10.1038/s41467-023-38840-8

**Published:** 2023-06-06

**Authors:** Wenfang Gui, Mikal Jacob Hole, Antonio Molinaro, Karolina Edlund, Kristin K. Jørgensen, Huan Su, Brigitte Begher-Tibbe, Nikolaus Gaßler, Carolin V. Schneider, Uthayakumar Muthukumarasamy, Antje Mohs, Lijun Liao, Julius Jaeger, Christian J. Mertens, Ina Bergheim, Till Strowig, Jan G. Hengstler, Johannes R. Hov, Hanns-Ulrich Marschall, Christian Trautwein, Kai Markus Schneider

**Affiliations:** 1 Department of Medicine III, University Hospital RWTH Aachen, Aachen, 52074 Germany; 2 Norwegian PSC Research Center, Section of Gastroenterology and Research Institute of Internal Medicine, Division of Surgery, Inflammatory Diseases and Transplantation, Oslo University Hospital and University of Oslo, Oslo, Norway; 3 Department of Medicine, Sahlgrenska University Hospital, Gothenburg, Sweden; 4 Norwegian PSC Research Center, Department of Transplantation Medicine, Oslo University Hospital, Rikshospitalet, Oslo, Norway; 5 Research Institute of Internal Medicine, Oslo University Hospital Rikshospitalet, Oslo, Norway; 6 Leibniz Research Centre for Working Environment and Human Factors, Technical University Dortmund, Dortmund, 44139 Germany; 7 Department of Gastroenterology, Akershus University Hospital, Lørenskog, Norway; 8 Institute for Legal Medicine, Section Pathology, University Hospital, Jena, 07747 Germany; 9 Helmholtz Centre for Infection Research, Braunschweig, Germany and Centre for Individualised Infection Medicine (CiiM), a joint venture between the Helmholtz-Centre for Infection Research (HZI) and the Hannover Medical School (MHH), Hannover, 97080 Germany; 10 Department of Anesthesiology and Pain Management, Shanghai East Hospital, School of Medicine, Tongji University, Shanghai, 200120 China; 11 Department of Nutritional Sciences, Molecular Nutritional Science, University of Vienna, Vienna, A-1090 Austria; 12 Department of Molecular and Clinical Medicine/Wallenberg Laboratory, Sahlgrenska Academy, University of Gothenburg, Gothenburg, 41345 Sweden

**Keywords:** Primary sclerosing cholangitis, Mechanisms of disease, Ulcerative colitis

## Abstract

Primary sclerosing cholangitis (PSC) is a chronic cholestatic liver disease characterized by chronic inflammation and progressive fibrosis of the biliary tree. The majority of PSC patients suffer from concomitant inflammatory bowel disease (IBD), which has been suggested to promote disease development and progression. However, the molecular mechanisms by which intestinal inflammation may aggravate cholestatic liver disease remain incompletely understood. Here, we employ an IBD-PSC mouse model to investigate the impact of colitis on bile acid metabolism and cholestatic liver injury. Unexpectedly, intestinal inflammation and barrier impairment improve acute cholestatic liver injury and result in reduced liver fibrosis in a chronic colitis model. This phenotype is independent of colitis-induced alterations of microbial bile acid metabolism but mediated via hepatocellular NF-κB activation by lipopolysaccharide (LPS), which suppresses bile acid metabolism in-vitro and in-vivo. This study identifies a colitis-triggered protective circuit suppressing cholestatic liver disease and encourages multi-organ treatment strategies for PSC.

## Abstract (web summary)

The association between primary sclerosing cholangitis (PSC) and inflammatory bowel disease (IBD) has been known for decades, but mechanisms of gut-liver crosstalk are incompletely understood. Here, the authors show a colitis-triggered protective circuit suppressing cholestatic liver disease which encourages multi-organ treatment strategies for PSC.

## Introduction

Primary sclerosing cholangitis (PSC) is one of the most common chronic cholestatic liver diseases. It is characterized by chronic inflammation and progressive fibrosis of the biliary tree^1^. Up to 80% of PSC patients suffer from concurrent inflammatory bowel disease (IBD) (especially ulcerative colitis)^2,3^. Conversely, only 2–10% of patients with IBD suffer from PSC^3–5^. PSC patients with concomitant IBD exhibit a distinct phenotype characterized by rectal sparing, comparatively quiescent colitis with right-side predominance, backwash ileitis, and increased incidence of colorectal malignancy compared to patients with isolated IBD^3,6,7^. Since there are no effective medical therapies to alter the natural course of PSC, patients often progress to liver cirrhosis and liver transplantation is the only therapeutic option for patients with end-stage PSC^8^.

The frequent coincidence of PSC and IBD suggests some intriguing crosstalk between the gut and liver^9^. Translocation of gut-derived bacterial antigens via the portal vein into the liver may trigger a hepatic immune response resulting in an impairment of the tight junction barrier between hepatocytes^10,11^. It has been proposed that bacteria and microbe-associated molecular patterns (MAMPs) such as lipopolysaccharide, from the portal blood can breach leaky hepatocyte tight junctions to enter the biliary tree. Hepatocyte tight junctions are regarded as a significant barrier between the portal circulation and biliary system. Loss of this barrier may result in bile duct damage, cholestasis, and eventually sclerosing cholangitis^11,12^. A role of lipopolysaccharide (LPS) is supported by histological study that found increased accumulation of LPS in PSC patients compared with controls^13^. Moreover, in a nationwide cohort study colectomy prior to diagnosis of primary sclerosing cholangitis was associated with improved prognosis^14^.

Contrary to these hypotheses, clinical research revealed that some patients develop PSC prior to their IBD diagnosis or even years after total colectomy. Moreover, serious colitis tends to be associated with less progressive PSC defined by the requirement for liver transplantation^15,16^. Further research is needed to uncover the causes of this discrepancy.

Our previous data demonstrated that *Mdr2*^*−/−*^ mice – a model of PSC – develop intestinal dysbiosis, which was partially transmissible to WT mice^17^. In more recent work we could show that microbiota depletion in adult *Mdr2*^*−/−*^ mice resulted in lethal cholestasis due to loss of microbial bile acid (BA) metabolism and subsequent ileal FXR activation^18^. Yet, the molecular pathways by which colitis affects bile acid metabolism and cholestatic liver disease remain incompletely understood. Using an IBD-PSC mouse model, we define a protective circuit by which colitis alleviates liver impairment and fibrosis of murine sclerosing cholangitis, a process mediated by suppression of BA metabolism via hepatic inflammatory signaling and more specifically NF-κB activation. Together, our study uncovers a context in which intestinal barrier impairment is protective and offers a new perspective on the long-standing IBD-PSC association.

## Results

### Acute experimental colitis improves hepatocyte injury and cholestasis in *Mdr2*^*−/−*^ mice

**Fig. 1:**
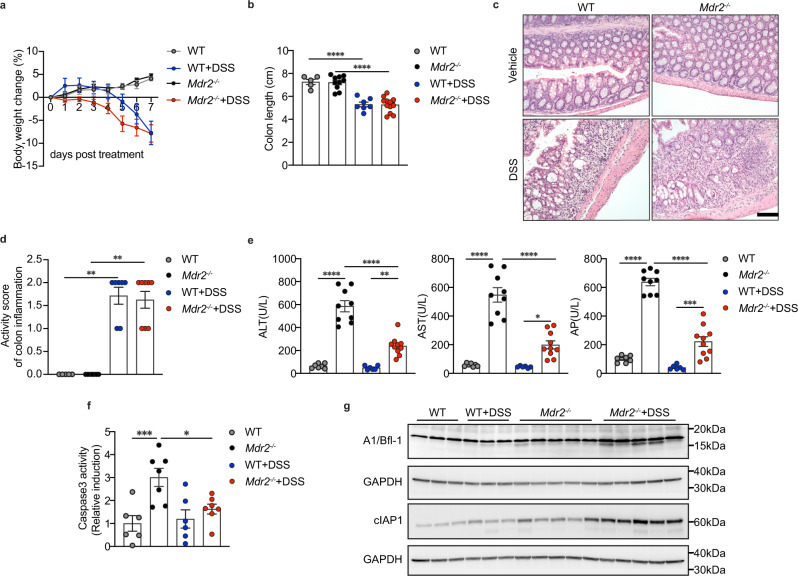
Acute experimental colitis alleviates cholestatic hepatocyte injury in *Mdr2*^*−/−*^ mice. **a** The body weight curves and (**b**) colon length of WT and *Mdr2*^*−/−*^ mice (WT, n = 5; *Mdr2*^*−/−*^,*n* = 10; WT + DSS, *n* = 7; *Mdr2*^*−/−*^+DSS, *n* = 12); one-way ANOVA with Bonferroni’s multiple comparison test (Colon length: WT vs WT + DSS: 95% CI 1.025–2.895, *P* < 0.0001; *Mdr2*^*−/−*^ vs *Mdr2*^*−/−*^+DSS, 95% CI 1.26–2.627, *P* < 0.0001). **c** Representative images of haematoxylin and eosin (H&E) stained distal colon (scale bar, 100 µm) (WT, *n* = 5; *Mdr2*^*−/−*^,n = 9; WT + DSS, n = 7; *Mdr2*^*−/−*^+DSS, n = 12). **d** Scoring of colon inflammation (WT, *n* = 5; *Mdr2*^*−/−*^, *n* = 7; WT + DSS, *n* = 7; *Mdr2*^*−/−*^+DSS, *n* = 8); Kruskal-Wallis test with Dunn’s multiple comparison test (WT vs WT + DSS, *P* = 0.0051; *Mdr2*^*−/−*^ vs *Mdr2*^*−/−*^+DSS, *P* = 0.0021). **e** Serum ALT, AST and AP levels (WT, *n* = 7; *Mdr2*^*−/−*^, *n* = 9; WT + DSS, *n* = 6; *Mdr2*^*−/−*^+DSS, *n* = 10) from 2 representative independent cohorts; one-way ANOVA with Bonferroni’s multiple comparison test (ALT: WT vs *Mdr2*^*−/−*^, 95% CI −645.6 to −399, *P* < 0.0001; *Mdr2*^*−/−*^ vs *Mdr2*^*−/−*^+DSS, 95% CI 234.5–459.3, *P* < 0.0001; WT + DSS vs vs *Mdr2*^*−/−*^+DSS, 95% CI −317 to −64.35, *P* = 0.0016; AST: WT vs *Mdr2*^*−/−*^, 95% CI −617.5 to −360.9, *P* < 0.0001; *Mdr2*^*−/−*^ vs *Mdr2*^*−/−*^+DSS, 95% CI 231.8 to 465.8, P < 0.0001; WT + DSS vs vs *Mdr2*^*−/−*^+DSS, 95% CI −284.7 to −21.68, *P* = 0.0171; AP: WT vs *Mdr2*^*−/−*^, 95% CI −638.1 to −437, *P* < 0.0001; *Mdr2*^*−/−*^ vs *Mdr2*^*−/−*^+DSS, 95% CI 323.9–507.2, *P* < 0.0001; WT + DSS vs vs *Mdr2*^*−/−*^+DSS, 95% CI −284.2 to −78.16, *P* = 0.0003). **f** Analysis of liver caspase 3 activity (WT, *n* = 6; *Mdr2*^*−/−*^, *n* = 7; WT + DSS, *n* = 6; *Mdr2*^*−/−*^+DSS, *n* = 7); one-way ANOVA with Bonferroni’s multiple comparison test (WT vs *Mdr2*^*−/−*^, 95% CI −3.174 to −0.8397, *P* = 0.0009; *Mdr2*^*−/−*^ vs *Mdr2*^*−/−*^+DSS, 95% CI 0.2597–2.503, *P* = 0.0144). **g** Western blot of liver anti-apoptotic A1/Bfl-1 and cIAP1 (WT, *n* = 3; *Mdr2*^*−/−*^, *n* = 5; WT + DSS, *n* = 3; *Mdr2*^*−/−*^+DSS, *n* = 5). All data are graphed as mean ± SEM and considered significant at *p < 0.05, **p < 0.01, ***p < 0.001 and ****p < 0.0001. Source data are provided as a Source Data file.

To identify pathways by which acute colitis affects cholestatic liver injury, we first treated 8–10-week-old WT and *Mdr2*^*−/−*^ mice with 2.5% DSS for 7 days. All mice tolerated this treatment well, and water consumption was comparable between groups. DSS caused acute colitis, which was reflected in weight loss, watery or bloody diarrhea, piloerection of fur, decreased movement and appetite, as well as colon shortening (Fig. 1a, b). The onset of weight loss in *Mdr2*^*−/−*^ mice was earlier, but on day 7 differences between WT and *Mdr2*^*−/−*^ disappeared. (Fig. 1a). Similarly, there was no significant difference in colonic shortening between the groups following DSS treatment (Fig. 1b).

As previously reported, most severe lesions were observed in distal colon after DSS injury (Fig. 1c), whereas no obvious architectural impairment was detected in the ileum and proximal colon (Supplementary Fig. 1a). Subsequently, colitis scores were quantified by a specialized bowel pathologist (N.G.). Control mice presented intact epithelium and mucosa, but DSS groups exhibited disruption of epithelial barrier with neutrophil infiltration and loss of goblet cells, resulting in cryptitis, crypt abscess as well as ulcer formation (acute activity), and ultimately leading to crypt distortion with band-like lamina propria lymphoplasmacytosis (chronic injury). Based on these features, the histological score of colon inflammation and chronic injury revealed no difference between WT DSS and *Mdr2*^*−/−*^ DSS animals (Fig. 1d, Supplementary Fig. 1b).

To investigate the impact of inflamed intestine on cholestatic liver injury, we measured serum liver enzymes. Unexpectedly, acute colitis significantly reduced markers of hepatocyte injury (ALT, AST, GLDH) and cholestasis (AP) in the *Mdr2*^*−/−*^ group (Fig. 1e, Supplementary Fig. 1c). To characterize the cell death, we performed a sensitive assay for detecting caspase 3 activity in liver homogenates. Consistent with liver enzymes, caspase 3 activity was significantly reduced in *Mdr2*^*−/−*^ DSS mice compared with untreated animals (Fig. 1f). And NF-κB-regulated anti-apoptotic A1/Bfl-1 and cIAP1 were upregulated in *Mdr2*^*−/−*^ liver after DSS treatment (Fig. 1g), which has been previously shown to suppress hepatocyte apoptosis in *Mdr2*^*−/−*^ mice^19,20^. Liver histology was assessed by a board-certified pathologist. Following colitis induction, biliary fibrosis, mitotic activity, and bile duct proliferation remained unchanged in *Mdr2*^*−/−*^ liver, which was in line with Sirius red staining and immunohistochemistry (IHC) of Ki67 and CK19 (Supplementary Fig. 1d, e).

Altogether, these results demonstrated a beneficial effect of DSS-induced acute colitis on hepatocyte injury and cholestasis in *Mdr2*^*−/−*^ mice.

### Colitis and intestinal barrier impairment trigger translocation of bacterial components and hepatic inflammation

**Fig. 2:**
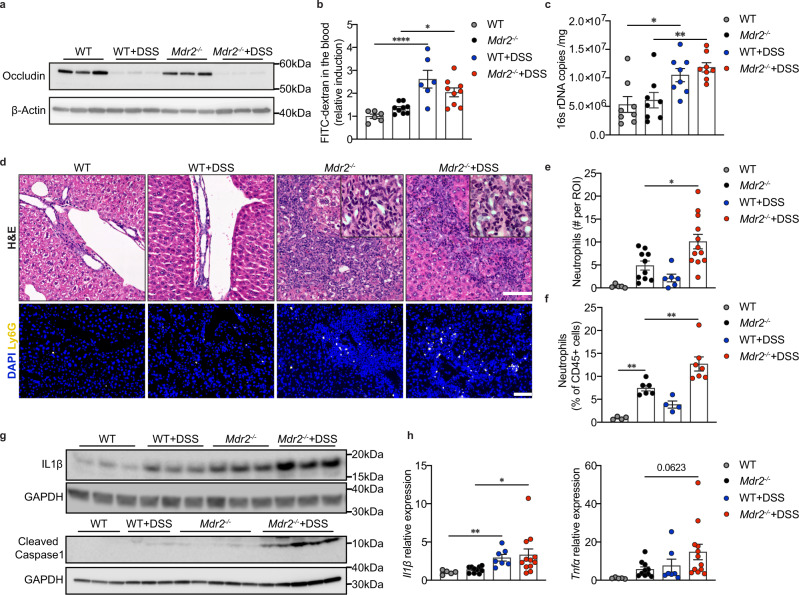
Colitis and intestinal barrier impairment trigger translocation of bacterial components and hepatic inflammation. **a** Images of colon Occludin western blot (*n* = 3 mice per group). **b** Analysis of FITC-dextran assay (WT, *n* = 6; *Mdr2*^*−/−*^, *n* = 9; WT + DSS, *n* = 6; *Mdr2*^*−/−*^+DSS, *n* = 9); one-way ANOVA with Bonferroni’s multiple comparison test (WT vs WT + DSS, 95% CI −2.428 to −0.8037, *P* < 0.0001; *Mdr2*^*−/−*^ vs *Mdr2*^*−/−*^+DSS, 95% CI −1.358 to −0.03156, *P* = 0.0378). **c** Measurement of total bacterial 16S rDNA in the frozen liver (*n* = 8 mice per group); one-way ANOVA with Bonferroni’s multiple comparison test (WT vs WT + DSS, 95% CI −9700599 to −601901, *P* = 0.0212; *Mdr2*^*−/−*^ vs *Mdr2*^*−/−*^+DSS, 95% CI −10300599 to −1201901, *P* = 0.0087). **d** Representative H&E and Ly6G stainings of liver sections (H&E: WT, *n* = 5; *Mdr2*^*−/−*^, *n* = 10; WT + DSS, *n* = 7; *Mdr2*^*−/−*^+DSS, *n* = 12; Ly6G: WT, *n* = 5; *Mdr2*^*−/−*^, *n* = 8; WT + DSS, *n* = 7; *Mdr2*^*−/−*^+DSS, *n* = 10) (scale bar,100 µm). **e** Quantification of liver neutrophils identified by their characteristic morphology in liver H&E staining (WT, *n* = 5; *Mdr2*^*−/−*^, *n* = 10; WT + DSS, *n* = 6; *Mdr2*^*−/−*^+DSS, *n* = 12); one-way ANOVA with Bonferroni’s multiple comparison test (*Mdr2*^*−/−*^ vs *Mdr2*^*−/−*^+DSS, 95% CI −9.688 to −0.7727, *P* = 0.0161). **f** The percentage of liver CD11b^+^Ly6G^+^ neutrophils to CD45^+^ leukocytes (WT, *n* = 4; *Mdr2*^*−/−*^, *n* = 6; WT + DSS, *n* = 4; *Mdr2*^*−/−*^+DSS, *n* = 7); one-way ANOVA with Bonferroni’s multiple comparison test (WT vs *Mdr2*^*−/−*^, 95% CI −11.32 to −1.761, *P* = 0.0055; *Mdr2*^*−/−*^ vs *Mdr2*^*−/−*^+DSS, 95% CI −9.374 to −1.138, *P* = 0.0095). **g** Hepatic protein expression of IL1β (*n* = 3 mice per group) and Cleaved Caspase1 (WT, *n* = 3; *Mdr2*^*−/−*^, *n* = 5; WT + DSS, *n* = 3; *Mdr2*^*−/−*^+DSS, *n* = 5). **h** Liver *Il1β and Tnfα* mRNA expression (WT, *n* = 5; *Mdr2*^*−/−*^, *n* = 10; WT + DSS, *n* = 7; *Mdr2*^*−/−*^+DSS, *n* = 12); Kruskal-Wallis test with Dunn’s multiple comparison test (*Il1β*: WT vs WT + DSS, *P* = 0.0065; *Mdr2*^*−/−*^ vs *Mdr2*^*−/−*^+DSS, *P* = 0.0231; *Tnfα: Mdr2*^*−/−*^ vs *Mdr2*^*−/−*^+DSS, *P* = 0.0623). All data are displayed as mean ± SEM and considered statistically significant at *p < 0.05, **p < 0.01, ***p < 0.001 and ****p < 0.0001. Source data are provided as a Source Data file.

Colitis induces intestinal barrier impairment, which has been shown to have detrimental effects on the liver and other organ systems^21,22^. As expected, immunofluorescence staining, qPCR and western blot confirmed that DSS profoundly decreased intestinal tight junction markers, such as Mucin2 (MUC2), ZO-1 and Occludin (Fig. 2a, Supplementary Fig. 2a, b). To assess intestinal barrier function, we first performed an in-vivo FITC-dextran assay. Consistent with impaired gut barrier, translocation of FITC-dextran was significantly increased in DSS treated WT and *Mdr2*^*−/−*^ mice (Fig. 2b). Furthermore, DSS treatment resulted in an increase of bacterial DNA content in these mice (Fig. 2c, Supplementary Fig. 2c). Together these data provide evidence for intestinal barrier impairment leading to increased translocation of bacterial components.

To elucidate the effect of a leaky gut on hepatic inflammation, histopathological examinations revealed increased neutrophils in the portal area of *Mdr2*^*−/−*^ DSS livers (Fig. 2d, e). Consistently, liver Ly6G^+^ and CD11b^+^ cell counts were significantly increased in *Mdr2*^*−/−*^ DSS mice as evidenced by immunofluorescence staining (Fig. 2d, Supplementary Fig. 3a–c). To further quantify the specific cellular composition, fluorescence-activated cell sorting (FACS) demonstrated the accumulation of neutrophils in livers of DSS-treated *Mdr2*^*−/−*^ mice (Fig. 2f). No differences were found in the number of monocyte-derived macrophages (MoMFs), B cells, CD4^+^ T cells, CD8^+^ T cells, NK cells, and NKT cells between *Mdr2*^*−/−*^ and *Mdr2*^*−/−*^ DSS animals (Supplementary Fig. 3d). Accordingly, DSS-fed *Mdr2*^*−/−*^ mice displayed significantly higher IL1β, cleaved caspase1 and NLRP3 protein levels (Fig. 2g, Supplementary Fig. 3e), in agreement with overexpression of *Il1β*, *Tnfα*, *Nlrp3*, *Nfkb*, *Ccl5*, *Icam-1*, *F4/80* and *Nos2* mRNA, while other inflammatory genes (*Tlr4*, *Ccl2*, *Ifng*, *Il12a*, *Il12b*, *Il10*, *Il6*, *Vcam-1*, *Cox-2*) showed only a trend (Fig. 2h, Supplementary Fig. 3f–h).

### Colitis dampens bile acid synthesis and transport in *Mdr2*^*−/−*^ mice

**Fig. 3:**
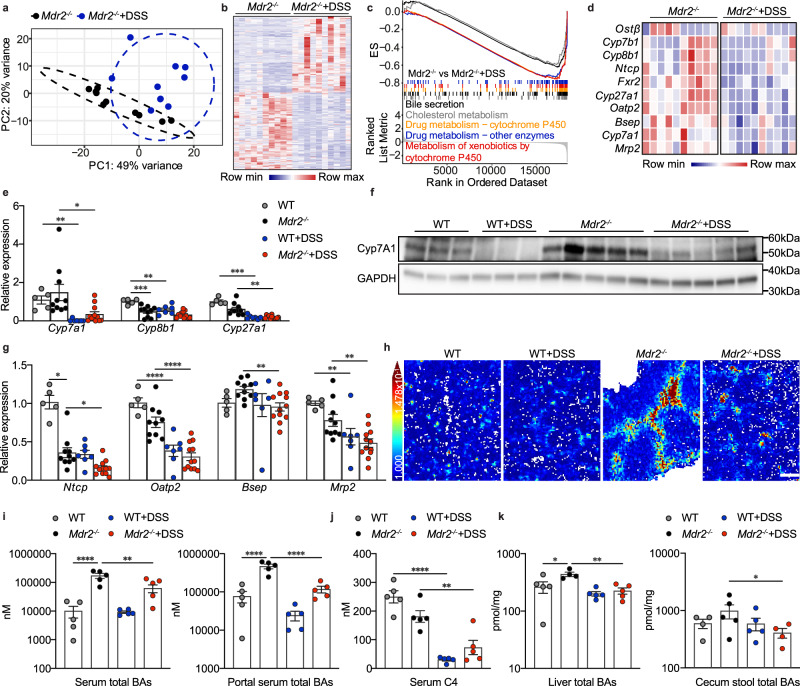
Colitis dampens bile acid synthesis and transport in *Mdr2*^*−/−*^ mice. **a** PCA (principal component analysis) plot of liver RNA-seq data (*n* = 10 mice per group). **b** Heatmap of differentially expressed genes from hepatic RNA-seq data (*n* = 10 mice per group); Wald test with Benjamini-Hochberg adjustment (two-sided) (padj < 0.05 & Log2foldchange > 1 or < −1). **c** Suppressed pathways in DSS-treated *Mdr2*^*−/−*^ liver identified by RNA-seq-based gene set enrichment analysis (*n* = 10 mice per group). **d** Heatmap of liver RNA-seq data shows downregulated genes (*n* = 10 mice per group). **e** mRNA expression of genes related to bile acid synthesis (WT, *n* = 5; *Mdr2*^*−/−*^, *n* = 10; WT + DSS, *n* = 7; *Mdr2*^*−/−*^+DSS, *n* = 12); one-way ANOVA with Bonferroni’s multiple comparison test (*Cyp8b1*: WT vs *Mdr2*^*−/−*^, 95% CI 0.223–0.827, *P* = 0.0004; WT vs WT + DSS, 95% CI 0.1056–0.7512, *P* = 0.0063); Kruskal-Wallis test with Dunn’s multiple comparison test (*Cyp7a1*: WT vs WT + DSS, P = 0.0011; *Mdr2*^*−/−*^ vs *Mdr2*^*−/−*^+DSS, *P* = 0.0197; *Cyp27a1*: WT vs WT + DSS, *P* = 0.0008; *Mdr2*^*−/−*^ vs *Mdr2*^*−/−*^+DSS, *P* = 0.0018). **f** Western blot of liver Cyp7A1 (WT, *n* = 3; *Mdr2*^*−/−*^, *n* = 5; WT + DSS, *n* = 3; *Mdr2*^*−/−*^+DSS, *n* = 5). **g** mRNA expression of genes involved in bile acid trafficking (WT, *n* = 5; *Mdr2*^*−/−*^, *n* = 10; WT + DSS, *n* = 7; *Mdr2*^*−/−*^+DSS, *n* = 12); one-way ANOVA with Bonferroni’s multiple comparison test (*Oatp2*: WT vs WT + DSS, 95% CI 0.3657–0.8851, *P* < 0.0001; *Mdr2*^*−/−*^ vs *Mdr2*^*−/−*^+DSS, 95% CI 0.2581–0.6379, *P* < 0.0001; *Mrp2*: WT vs WT + DSS, 95% CI 0.1302 to 0.7397, *P* = 0.0042; *Mdr2*^*−/−*^ vs *Mdr2*^*−/−*^+DSS, 95% CI 0.0725–0.5182, *P* = 0.0078); Kruskal-Wallis test with Dunn’s multiple comparison test (*Ntcp*: WT vs *Mdr2*^*−/−*^, *P* = 0.0361; *Mdr2*^*−/−*^ vs *Mdr2*^*−/−*^+DSS, *P* = 0.0384; *Bsep*: *Mdr2*^*−/−*^ vs *Mdr2*^*−/−*^+DSS, *P* = 0.0051). **h** Spatial distribution of taurocholic acid in the liver visualized by Matrix-assisted laser desorption/ionization mass spectrometry imaging. (WT, *n* = 5; *Mdr2*^*−/−*^, *n* = 6; WT + DSS, *n* = 7; *Mdr2*^*−/−*^+DSS, *n* = 7) (scale bar, 300 µm; color bar, 147.8–1000). **i** Total bile acids of serum and portal serum (*n* = 5 mice per group); one-way ANOVA with Bonferroni’s multiple comparison test (serum total BAs: WT vs *Mdr2*^*−/−*^, 95% CI −238,281 to −83,938, *P* < 0.0001; *Mdr2*^*−/−*^ vs *Mdr2*^*−/−*^+DSS, 95% CI 31,605–185,948, *P* = 0.0044; portal serum total BAs: WT vs *Mdr2*^*−/−*^, 95% CI −537,812 to −240,590, *P* < 0.0001; *Mdr2*^*−/−*^ vs *Mdr2*^*−/−*^+DSS, 95% CI 200,324–497,546, *P* < 0.0001). **j** C4 in serum (*n* = 5 mice per group); one-way ANOVA with Bonferroni’s multiple comparison test (WT vs WT + DSS, 95% CI 141.7–296.5, *P* < 0.0001; *Mdr2*^*−/−*^ vs *Mdr2*^*−/−*^+DSS, 95% CI 30.64–185.4, *P* = 0.0048). **k** Total bile acids of liver and cecum stool (*n* = 5 mice per group. For technical reason, bile acids can not be assayed in few samples leading to different sample sizes); one-way ANOVA with Bonferroni’s multiple comparison test (liver total BAs: WT vs *Mdr2*^*−/−*^, 95% CI −338.6 to −11.27, *P* = 0.0336; *Mdr2*^*−/−*^ vs *Mdr2*^*−/−*^+DSS, 95% CI 49.95–377.2, *P* = 0.0085; total BAs of cecum stool: *Mdr2*^*−/−*^ vs *Mdr2*^*−/−*^+DSS, 95% CI 17.64–1,138, *P* = 0.0441).All data are expressed as mean ± SEM and considered statistically significant at *p < 0.05, **p < 0.01, ***p < 0.001 and ****p < 0.0001. Source data are provided as a Source Data file.

To investigate the mechanism by which DSS-induced colitis improves cholestatic liver injury in *Mdr2*^*−/−*^ mice, liver RNA sequencing analysis was performed. Principal component analysis (PCA) compared global gene expression signatures and revealed that the transcriptomic profiles of *Mdr2*^*−/−*^ DSS livers were separated from those of *Mdr2*^*−/−*^ control mice (Fig. 3a). 196 genes were significantly downregulated, while 193 genes were markedly upregulated in *Mdr2*^*−/−*^ DSS livers compared to *Mdr2*^*−/−*^ controls (Fig. 3b, Supplementary Fig. 5a).

To explore global changes in transcriptomic programs upon colitis induction in an unbiased way, we performed gene set enrichment analysis (GSEA). Interestingly, GSEA uncovered a suppression of pathways involved in bile secretion, cholesterol metabolism, drug or xenobiotic metabolism by cytochrome P450 in DSS-treated *Mdr2*^*−/−*^ liver tissues (Fig. 3c), possibly contributing to the inhibition of bile acid synthesis and transport. Consistently, several genes involved in bile acid synthesis and its regulation were strongly downregulated in these mice (Fig. 3d). To validate the results from RNA-seq analysis, real-time PCR was performed to quantify hepatic expression of *Cyp7a1* and *Cyp27a1*, two genes encoding enzymes for bile acid synthesis, showing a strong reduction in *Mdr2*^*−/−*^ mice after DSS treatment (Fig. 3e). While *Cyp8b1* was only slightly decreased (Fig. 3e), Cyp7A1 protein expression was strongly reduced in DSS-treated *Mdr2*^*−/−*^ livers (Fig. 3f). In addition, hepatic RT-qPCR showed a reduction of sinusoidal BA importers (*Ntcp* and *Oatp2*) as well as canalicular BA exporters (*Bsep* and *Mrp2*) (Fig. 3g), with no impact on basolateral BA efflux carriers (*Mrp3*, *Mrp4*, and *Ostα*–*Ostβ* complex) (Supplementary Fig. 5b).

Next, we evaluated whether these genetic changes resulted in shifts of bile acid distribution and composition. To this end, we first performed matrix-assisted laser desorption/ionization mass spectrometry imaging (MALDI-MSI) to visualize the spatial distribution of taurocholic acid (TCA), the most enriched bile acid in mice. For identification of the periportal area, MALDI images were superimposed with adjacent liver sections stained by CK19.

As expected, untreated *Mdr2*^*−/−*^ mice exhibited increased TCA intensity mostly in the periportal area compared to the WT group. Upon DSS treatment, hepatic bile acid accumulation was significantly reduced in *Mdr2*^*−/−*^ mice, while TCA signal was not strikingly altered in WT mice (Fig. 3h, Supplementary Fig. 5c, d). To validate these findings, bile acids were measured in different serum compartments using HPLC-MS/MS. In line with MALDI and gene expression data, DSS dramatically lowered total bile acids in the blood taken from the cava or portal vein of *Mdr2*^*−/−*^ mice (Fig. 3i). Similarly, DSS treatment also decreased concentrations of the bile acid intermediate 7α-hydroxy-4-cholesten-3-one (C4), a surrogate marker for bile acid synthesis^23^ (Fig. 3j). In addition, *Mdr2*^*−/−*^ mice exposed to DSS had significantly lower total BA levels in their liver and cecum stool (Fig. 3k).

In conclusion, DSS-triggered acute colitis induces a pronounced suppression of hepatic transcriptional programs involved in BA synthesis, uptake and secretion, which results in reduced hepatic bile acid accumulation and systemic BA levels.

### DSS-triggered liver inflammation mediates alterations of BA metabolism

**Fig. 4:**
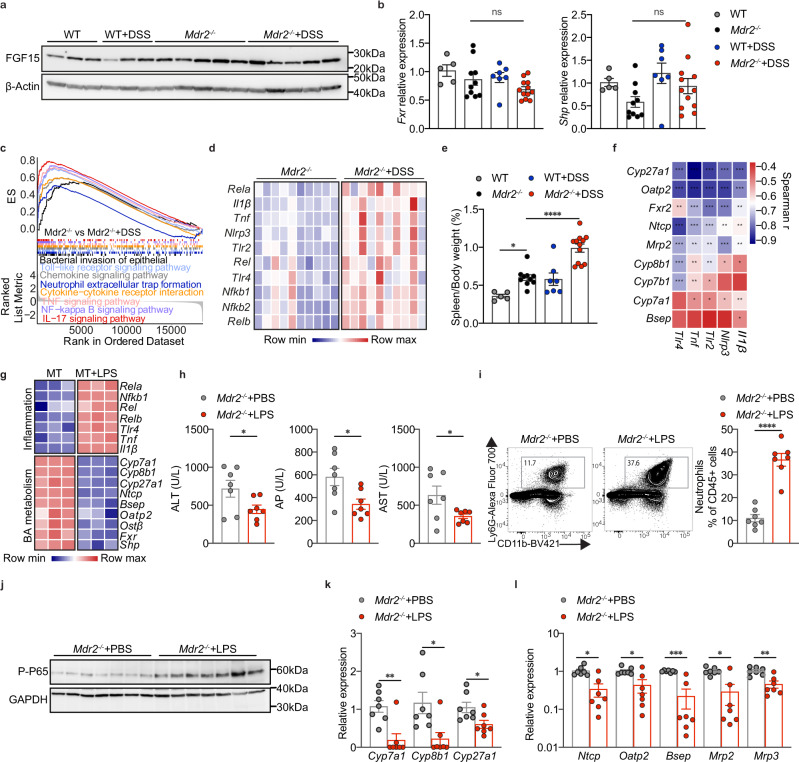
DSS-triggered liver inflammation mediates alterations of BA metabolism. **a** Western blot of distal ileum FGF15 (WT, *n* = 3; *Mdr2*^*−/−*^, *n* = 5; WT + DSS, *n* = 3; *Mdr2*^*−/−*^+DSS, *n* = 5). **b** mRNA expression of liver *Fxr* and *Shp* (WT, *n* = 5; *Mdr2*^*−/−*^, *n* = 10; WT + DSS, *n* = 7; *Mdr2*^*−/−*^+DSS, *n* = 12); one-way ANOVA with Bonferroni’s multiple comparison test (*Fxr & Shp*: no statistical difference between *Mdr2*^*−/−*^ vs *Mdr2*^*−/−*^+DSS). **c** Activated pathways in DSS-treated *Mdr2*^*−/−*^ liver identified by RNA-seq-based gene set enrichment analysis (*n* = 10 mice per group). **d** Heatmap of liver RNA-seq data demonstrates upregulated inflammatory genes (*n* = 10 mice per group). **e** Spleen to body weight ratio (WT, *n* = 5; *Mdr2*^*−/−*^, *n* = 10; WT + DSS, *n* = 7; *Mdr2*^*−/−*^+DSS, *n* = 12); one-way ANOVA with Bonferroni’s multiple comparison test (WT vs *Mdr2*^*−/−*^, 95% CI −0.4801 to −0.007131, *P* = 0.0412; *Mdr2*^*−/−*^ vs *Mdr2*^*−/−*^+DSS, 95% CI −0.5723 to −0.2025, *P* < 0.0001). **f** Spearman correlation heatmap reveals negative correlation between liver inflammatory genes and BA metabolism genes(*Mdr2*^*−/−*^, *n* = 10; *Mdr2*^*−/−*^+DSS, *n* = 10). **g** Heatmap of differentially expressed genes from microarray analysis of co-cultured human primary hepatocytes and Kupffer cells treated with or without LPS in 3-dimentional Human Liver Microtissues (MT) (*n* = 3 samples per group). **h** Serum ALT, AP and AST levels (*n* = 7 mice per group); unpaired two-tailed Student’s t-test (ALT: *Mdr2*^*−/−*^+PBS vs *Mdr2*^*−/−*^+LPS, *P* = 0.0494; AP: *Mdr2*^*−/−*^+PBS vs *Mdr2*^*−/−*^+LPS, *P* = 0.0186; AST: *Mdr2*^*−/−*^+PBS vs *Mdr2*^*−/−*^+LPS, *P* = 0.0432). **i** The percentage of liver neutrophils to CD45^+^ leukocytes (*n* = 7 mice per group); unpaired two-tailed Student’s t-test (*Mdr2*^*−/−*^+PBS vs *Mdr2*^*−/−*^+LPS, *P* < 0.0001). **j** Western blot of liver phosphorylated NF-κB P65 (*n* = 7 mice per group). **k** mRNA expression of hepatic genes responsible for bile acid synthesis (*n* = 7 mice per group); unpaired two-tailed Student’s t-test (*Mdr2*^*−/−*^+PBS vs *Mdr2*^*−/−*^+LPS: *Cyp27a1*, *P* = 0.0247); Two-tailed Mann–Whitney test (*Mdr2*^*−/−*^+PBS vs *Mdr2*^*−/−*^+LPS: *Cyp7a1*, *P* = 0.007; *Cyp8b1*, *P* = 0.0111). **l** mRNA expression of hepatic genes associated with bile acid transporter (*n* = 7 mice per group); unpaired two-tailed Student’s t-test (*Mdr2*^*−/−*^+PBS vs *Mdr2*^*−/−*^+LPS: *Mrp3*, *P* = 0.0011); Two-tailed Mann–Whitney test (*Mdr2*^*−/−*^+PBS vs *Mdr2*^*−/−*^+LPS: *Ntcp*, *P* = 0.0111; *Oatp2*, *P* = 0.0175; *Bsep*, *P* = 0.0006; *Mrp2*, *P* = 0.0111). All data are mean ± SEM and considered significant at *p < 0.05, **p < 0.01, ***p < 0.001 and ****p < 0.0001. Source data are provided as a Source Data file.

Bile acid synthesis is controlled by a negative feedback from ileum via FXR-FGF15-FGFR4 and liver FXR-SHP^24^. We hypothesized that colitis might activate this intestinal negative feedback loop. However, ileal FGF15 protein levels did not differ between the *Mdr2*^*−/−*^ and *Mdr2*^*−/−*^ DSS groups (Fig. 4a, Supplementary Fig. 6a), which was consistent with no alterations of ileum FXR regulated genes (*Asbt* and *Ostα*–*Ostβ* complex) (Supplementary Fig. 6b). Additionally, DSS treatment had no impact on hepatic mRNA expression of *Fxr* and *Shp* (Fig. 4b). These findings imply that neither ileal nor hepatic FXR activation caused an inhibition of bile acid synthesis.

TNFα is known to profoundly modulate genes relevant for bile acid metabolism in sepsis-induced cholestasis^25^. To further address the mechanism by which DSS triggered a modification of bile acid metabolism, we hypothesized that inflammatory signaling induced changes of hepatobiliary genes in *Mdr2*^*−/−*^ DSS animals. Gene sets linked to inflammatory pathways including bacterial invasion of epithelial cells, toll-like receptor signaling, chemokine signaling, neutrophil extracellular trap formation, cytokine-cytokine receptor interaction, TNF, NF-κB, and IL-17 signaling were among the most significantly upregulated pathways in *Mdr2*^*−/−*^ DSS livers (Fig. 4c). Likewise, several inflammatory genes were upregulated (Fig. 4d) and an increased spleen to body weight ratio indicated systemic immune activation and gut toxin translocation after DSS treatment in *Mdr2*^*−/−*^ animals (Fig. 4e). Most interestingly, spearman correlation revealed a negative correlation between inflammatory genes (*Tnf*, *Il1β*, *Nlrp3*, *Tlrs*) and genes associated with BA metabolism (*Cyp7a1*, *Cyp8b1*, *Cyp27a1*, *Cyp7b1*, *Fxr2*, *Ntcp*, *Oatp2*, *Bsep*, *Mrp2*) (Fig. 4f).

Subsequently, we sought to extend these observations by exploring the ex vivo impact of inflammation on hepatocellular BA homeostasis. To this end, we analyzed the transcriptomic data from primary human hepatocytes that were cocultured with Kupffer cells in a three-dimensional multicellular microtissue (Jiang et al., 2019). Similar to what was observed in the *Mdr2*^*−/−*^ liver, LPS stimulation resulted in a strong downregulation of genes involved in bile acid transport and synthesis in this in-vitro system (Fig. 4g).

To expand the effect of translocation of bacterial components in vivo, we injected LPS into 8-week-old *Mdr2*^*−/−*^ mice. Strikingly, LPS treatment phenocopied the effects of colitis. *Mdr2*^*−/−*^ LPS mice exhibited remission of cholestatic hepatocyte impairment as evidenced by significantly lower levels of serum AST, ALT and AP (Fig. 4h). However, caspase 3 activity was significantly increased in the LPS injected *Mdr2*^*−/−*^ liver (Supplementary Fig. 6c). To further dissect which cell type was engaged in apoptosis, we performed immunohistochemistry stainings. Expression of cleaved caspase 3 was detected predominantly in immune cells at the portal area of LPS injected mice (Supplementary Fig. 6d). Importantly, similar to the acute colitis model, anti-apoptotic A1/Bfl-1 and cIAP1 proteins were upregulated in LPS injected *Mdr2*^*−/−*^ mice compared to controls (Supplementary Fig. 6e, f). Moreover, co-staining of TUNEL and HNF4α (hepatocyte nuclear factor 4α) revealed less TUNEL positive hepatocytes in LPS injected *Mdr2*^*−/−*^ mice (Supplementary Fig. 6g). These data demonstrate that LPS injection results in pronounced apoptosis of immune cells, but not hepatocytes. Liver FACS was used to elucidate the inflammatory response triggered by various immune cell types. In the LPS-injected *Mdr2*^*−/−*^ livers, numbers of infiltrating neutrophils were dramatically increased (Fig. 4i), along with NF-κB activation evidenced by increased protein expression and hepatocyte nuclear translocation of phosphorylated NF-κB P65 (Fig. 4j, Supplementary Fig. 6h, i), as well as higher expression of inflammatory genes (*Nfκb*, *Tnfα*, *Il1β*, *Nlrp3*, *Ccl5*, *Ccl2*, *Ifng*, *Il12a*, *Il10*, *Il6*, *Icam-1*, *Vcam-1*, *Nos2 and Cox-2*) (Supplementary Fig. 6j–l).

Since we had discovered that DSS induced inhibition of bile acid synthesis, we explored whether this also took place in LPS-treated *Mdr2*^*−/−*^ mice. In line with the previous DSS model, mRNA levels of genes encoding critical enzymes Cyp7A1, Cyp8B1 and Cyp27A1 were significantly downregulated in LPS injected *Mdr2*^*−/−*^ mice (Fig. 4k). Additionally, bile acid transporters (*Ntcp*, *Oatp2*, *Bsep*, *Mrp2* and *Mrp3*) were also notably decreased (Fig. 4l), whereas only a slight reduction was observed among genes responsible for FXR signaling (*Fxr* and *Shp*) (Supplementary Fig. 6l).

Overall, these results indicate that changes in bile acid homeostasis occur independently of feedback suppression via ileum or liver FXR signaling but are caused by DSS-induced hepatic inflammation.

### Abrogation of NF-κB signaling aggravates cholestasis in *Mdr2*^*−/−*^ mice

**Fig. 5:**
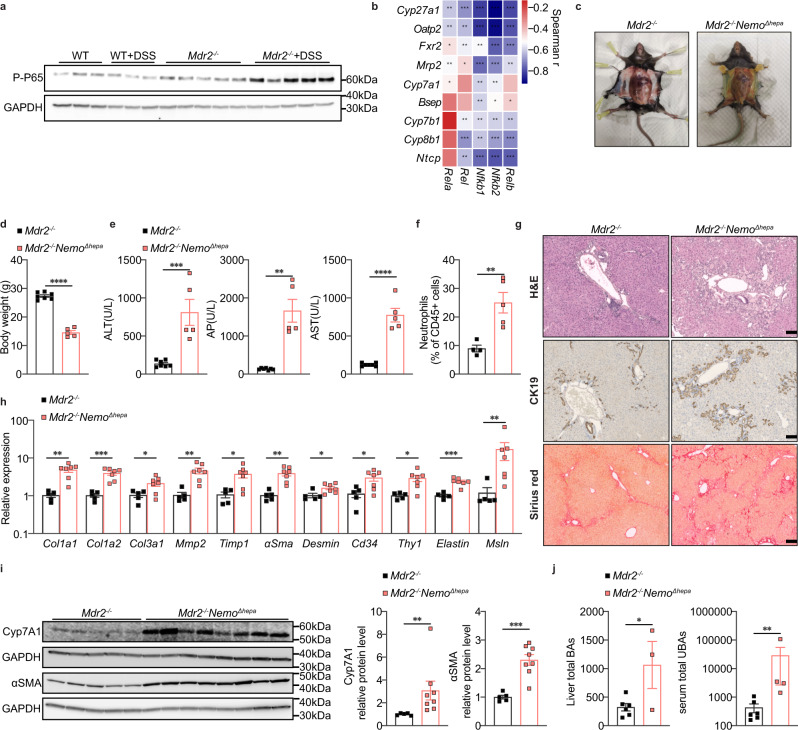
Abrogation of NF-κB signaling aggravates cholestasis in *Mdr2*^*−/−*^ mice. **a** Western blot of liver phosphorylated NF-κB P65 (WT, *n* = 3; *Mdr2*^*−/−*^, *n* = 5; WT + DSS, *n* = 3; *Mdr2*^*−/−*^+DSS, *n* = 5). **b** Spearman correlation heatmap of RNA-seq analysis demonstrates negative correlation between liver *Nfkb* and BA metabolism genes(*Mdr2*^*−/−*^, *n* = 10; *Mdr2*^*−/−*^+DSS, *n* = 10). **c** Representative images of *Mdr2*^*−/−*^ and *Mdr2*^*−/−*^*Nemo*^*Δhepa*^ mice. **d** Body weight of *Mdr2*^*−/−*^ and *Mdr2*^*−/−*^*Nemo*^*Δhepa*^ mice (*Mdr2*^*−/−*^, *n* = 7; *Mdr2*^*−/−*^*Nemo*^*Δhepa*^, *n* = 5); unpaired two-tailed Student’s t-test (*Mdr2*^*−/−*^ vs *Mdr2*^*−/−*^*Nemo*^*Δhepa*^, *P* < 0.0001). **e** Serum ALT, AP and AST levels (*Mdr2*^*−/−*^, *n* = 7; *Mdr2*^*−/−*^*Nemo*^*Δhepa*^, *n* = 5); unpaired two-tailed Student’s t-test (ALT: *Mdr2*^*−/−*^ vs *Mdr2*^*−/−*^*Nemo*^*Δhepa*^, *P* = 0.0008; AST: *Mdr2*^*−/−*^ vs *Mdr2*^*−/−*^*Nemo*^*Δhepa*^, *P* < 0.0001); Two-tailed Mann–Whitney test (AP: *Mdr2*^*−/−*^ vs *Mdr2*^*−/−*^*Nemo*^*Δhepa*^, *P* = 0.0025). **f** The percentage of hepatic neutrophils to CD45^+^ leukocytes (*Mdr2*^*−/−*^, *n* = 4; *Mdr2*^*−/−*^*Nemo*^*Δhepa*^, *n* = 5); unpaired two-tailed Student’s t-test (*Mdr2*^*−/−*^ vs *Mdr2*^*−/−*^*Nemo*^*Δhepa*^, *P* = 0.0064). **g** Representative images of liver H&E, CK19 and Sirius red stainings (*Mdr2*^*−/−*^, *n* = 8; *Mdr2*^*−/−*^*Nemo*^*Δhepa*^, *n* = 6) (scale bar, 100 µm). **h** Liver mRNA expression of fibrotic markers (*Mdr2*^*−/−*^, *n* = 5; *Mdr2*^*−/−*^*Nemo*^*Δhepa*^, *n* = 7); unpaired two-tailed Student’s t-test (*Mdr2*^*−/−*^ vs *Mdr2*^*−/−*^*Nemo*^*Δhepa*^: *Col1a1*, *P* = 0.0013; *Col1a2*, *P* = 0.0006; *Col3a1*, *P* = 0.035; *Mmp2*, *P* = 0.0047; *Timp1*, *P* = 0.0181; *αSma*, *P* = 0.0025; *Cd34*, *P* = 0.0247; *Thy1*, *P* = 0.0164; *Elastin*, *P* = 0.0002); Two-tailed Mann–Whitney test (*Mdr2*^*−/−*^ vs *Mdr2*^*−/−*^*Nemo*^*Δhepa*^: *Desmin*, *P* = 0.0303; *Msln*, *P* = 0.0051). **i** Western blot of liver Cyp7A1 and αSMA (*Mdr2*^*−/−*^, *n* = 5; *Mdr2*^*−/−*^*Nemo*^*Δhepa*^, *n* = 8); unpaired two-tailed Student’s t-test (αSMA: *Mdr2*^*−/−*^ vs *Mdr2*^*−/−*^*Nemo*^*Δhepa*^, *P* = 0.0003); Two-tailed Mann–Whitney test (Cyp7A1: *Mdr2*^*−/−*^ vs *Mdr2*^*−/−*^*Nemo*^*Δhepa*^, *P* = 0.0016). **j** Bile acids measurement of liver (*Mdr2*^*−/−*^, *n* = 5; *Mdr2*^*−/−*^*Nemo*^*Δhepa*^, *n* = 3) and serum (*Mdr2*^*−/−*^, *n* = 5; *Mdr2*^*−/−*^*Nemo*^*Δhepa*^, *n* = 4); unpaired two-tailed Student’s t-test (Liver total BAs: *Mdr2*^*−/−*^ vs *Mdr2*^*−/−*^*Nemo*^*Δhepa*^, *P* = 0.0359); Two-tailed Mann–Whitney test (Serum total UBAs: *Mdr2*^*−/−*^ vs *Mdr2*^*−/−*^*Nemo*^*Δhepa*^, *P* = 0.0095). All data are mean ± SEM and considered significant at *p < 0.05, **p < 0.01, ***p < 0.001 and ****p < 0.0001. Source data are provided as a Source Data file.

We hypothesized that NF-κB is an essential modulator to prevent cholestasis in *Mdr2*^*−/−*^ mice, because protein levels and hepatocyte nuclear translocation of phosphorylated NF-κB P65 were markedly elevated in the liver tissues of *Mdr2*^*−/−*^ DSS animals (Fig. 5a, Supplementary Fig. 7a–c), and RNA-seq data showed a pronounced negative correlation between *Nfκb* and genes involved in BA metabolism (Fig. 5b).

Hence, we generated *Mdr2*^*−/−*^*Nemo*^*Δhepa*^ DKO mice to investigate whether NEMO-mediated NF-κB activation is the pivotal downstream effector contributing to colitis-induced inhibition of bile acid synthesis in *Mdr2*^*−/−*^ animals. *Mdr2*^*−/−*^*Nemo*^*Δhepa*^ mice had very low reproductive potential. At a young age, they suffered from severe jaundice, body weight loss, and even spontaneous death (Fig. 5c, d), which prohibited further colitis induction. Even in homeostasis, NF-κB deficiency exacerbated cholestatic liver injury as indicated by elevated plasma parameters ALT, AP and AST (Fig. 5e).

Flow cytometry showed an accumulation of neutrophils in *Mdr2*^*−/−*^*Nemo*^*Δhepa*^ livers (Fig. 5f). However, hepatocytic NEMO deletion had minor effect on liver inflammatory pathways compared to *Mdr2*^*−/−*^ controls (Supplementary Fig. 7d-f). This is potentially due to the inability of hepatocytes to activate NF-κB in the absence of NEMO leading to reduced inflammatory gene expression. Moreover, as previously reported, NEMO deficiency can alter hepatic lipid metabolism^26^, which has been shown to alleviate inflammation and fibrosis in *Mdr2*^*−/−*^ mice following a high-fat diet feeding^27^. Histological examination of *Mdr2*^*−/−*^*Nemo*^*Δhepa*^ liver sections revealed extensive bile duct proliferation and worsened fibrosis as determined by H&E, CK19 and Sirius red stainings (Fig. 5g, Supplementary Fig. 7g-h). Since myofibrolasts originating from hepatic stellate cells (HSC) and portal fibroblasts (PF), but not fibrocytes, play a critical role in extracellular matrix deposition of *Mdr2*^*−/−*^ mice^28^, we next studied the fibrogenic response. Analysis of gene expression revealed an upregulation of pro-fibrotic markers (*Col1a1*, *Col1a2*, *Col3a1*, *Mmp2*), activated HSC markers (*Timp1*, *αSma*, *Desmin*) and PF markers (*Cd34*, *Thy1*, *Elastin*, *Msln*) in *Mdr2*^*−/−*^*Nemo*^*Δhepa*^ mice compared with *Mdr2*^*−/−*^ controls (Fig. 5h). Consistently, western blot analysis revealed significantly increased αSMA protein expression (Fig. 5i).

Importantly in the absence of *NEMO*, *Mdr2*^*−/−*^ mice failed to downregulate BA synthesis despite the strong biliary inflammation. Conversely, we found a remarkable upregulation of Cyp7A1 in *Mdr2*^*−/−*^*Nemo*^*Δhepa*^ mice at the protein level (Fig. 5i). Corresponding to changes observed in the rate-limiting enzyme of BA synthesis, *NEMO* deletion led to increased levels of total BAs in liver and total unconjugated BAs in serum (Fig. 5j).

Together, these findings indicate that hepatocellular NF-κB limits cholestasis in *Mdr2*^*−/−*^ mice and serves as a downstream mediator of the protective effect of colitis.

### Chronic colitis attenuates liver disease progression in *Mdr2*^*−/−*^ mice

**Fig. 6:**
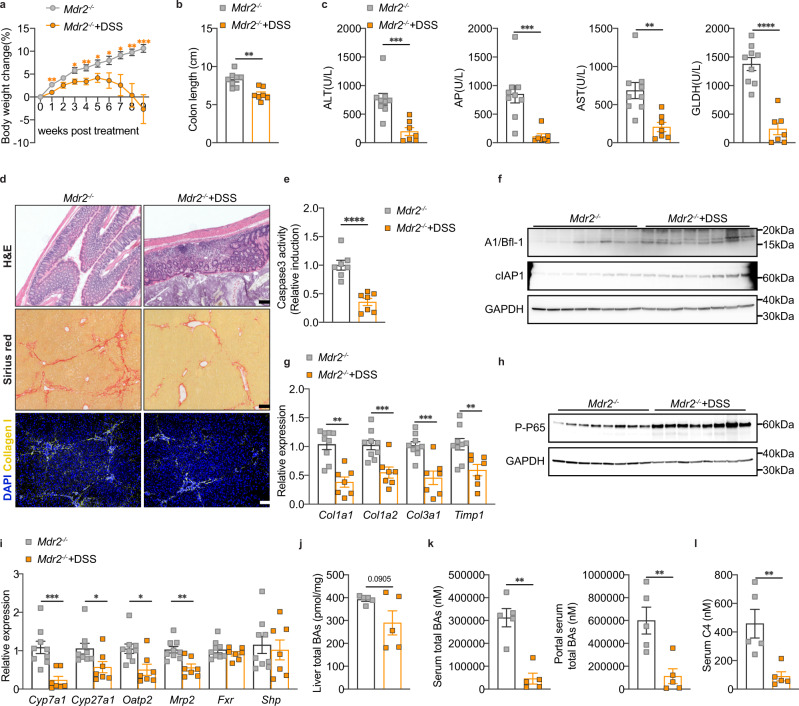
Chronic colitis attenuates liver disease progression in *Mdr2*^*−/−*^ mice. **a** Body weight change of *Mdr2*^*−/−*^ mice after 1% DSS treatment for 9 weeks (*Mdr2*^*−/−*^, *n* = 9; *Mdr2*^*−/−*^+DSS, *n* = 7); unpaired two-tailed Student’s t-test (*Mdr2*^*−/−*^ vs *Mdr2*^*−/−*^+DSS: week1, *P* = 0.004; week3, *P* = 0.0143; week4, *P* = 0.0094; week5, *P* = 0.0242; week6, *P* = 0.0279; week8, P = 0.0016; week9, *P* = 0.0005); Two-tailed Mann–Whitney test (*Mdr2*^*−/−*^ vs *Mdr2*^*−/−*^+DSS: week7, *P* = 0.0229). **b** Colon length of *Mdr2*^*−/−*^ mice with or without 1% DSS treatment (*Mdr2*^*−/−*^, *n* = 8; *Mdr2*^*−/−*^+DSS, *n* = 7); unpaired two-tailed Student’s t-test (*Mdr2*^*−/−*^ vs *Mdr2*^*−/−*^+DSS, *P* = 0.001). **c** Serum ALT, AP, AST and GLDH levels (*Mdr2*^*−/−*^, *n* = 9; *Mdr2*^*−/−*^+DSS, *n* = 7); unpaired two-tailed Student’s t-test (*Mdr2*^*−/−*^ vs *Mdr2*^*−/−*^+DSS: AST, *P* = 0.0028; GLDH, *P* < 0.0001); Two-tailed Mann–Whitney test (*Mdr2*^*−/−*^ vs *Mdr2*^*−/−*^+DSS: ALT, *P* = 0.0007; AP, *P* = 0.0003). **d** Representative images of H&E stained distal colon (*Mdr2*^*−/−*^, *n* = 9; *Mdr2*^*−/−*^+DSS, *n* = 7), liver Sirius red (*Mdr2*^*−/−*^, *n* = 9; *Mdr2*^*−/−*^+DSS, *n* = 7) and collagen I stainings (*Mdr2*^*−/−*^, *n* = 8; *Mdr2*^*−/−*^+DSS, *n* = 7) (scale bar, 100 µm). **e** Analysis of liver caspase 3 activity (*n* = 7 mice per group); unpaired two-tailed Student’s t-test (*Mdr2*^*−/−*^ vs *Mdr2*^*−/−*^+DSS, *P* < 0.0001). **f** Western blot of liver A1/Bfl-1 and cIAP1 (*n* = 8 mice per group); **g** Real-time PCR analysis of fibrotic genes (*Mdr2*^*−/−*^, *n* = 9; *Mdr2*^*−/−*^+DSS, *n* = 7); unpaired two-tailed Student’s t-test (*Mdr2*^*−/−*^ vs *Mdr2*^*−/−*^+DSS: *Col1a2*, *P* = 0.0018; *Col3a1*, *P* = 0.0006; *Timp1*, *P* = 0.007); Two-tailed Mann–Whitney test (*Mdr2*^*−/−*^ vs *Mdr2*^*−/−*^+DSS: *Col1a1*, *P* = 0.0007). **h** Western blot of liver phosphorylated NF-κB P65 (*n* = 8 mice per group). **i** Gene expression related to bile acid metabolism (*Mdr2*^*−/−*^, *n* = 9; *Mdr2*^*−/−*^+DSS, *n* = 7); unpaired two-tailed Student’s t-test (*Mdr2*^*−/−*^ vs *Mdr2*^*−/−*^+DSS: *Mrp2*, *P* = 0.0023; *Fxr & Shp*, no statistical differences); Two-tailed Mann–Whitney test (*Mdr2*^*−/−*^ vs *Mdr2*^*−/−*^+DSS: *Cyp7a1*, *P* = 0.0007; *Cyp27a1*, *P* = 0.0311; *Oatp2*, *P* = 0.0229). **j** Total bile acids of liver (*n* = 5 mice per group); unpaired two-tailed Student’s t-test (*Mdr2*^*−/−*^ vs *Mdr2*^*−/−*^+DSS: *P* = 0.0905). **k** Total bile acids of serum and portal serum (*n* = 5 mice per group); unpaired two-tailed Student’s t-test (*Mdr2*^*−/−*^ vs *Mdr2*^*−/−*^+DSS: portal serum total BAs, *P* = 0.0068); Two-tailed Mann–Whitney test (*Mdr2*^*−/−*^ vs *Mdr2*^*−/−*^+DSS: serum total BAs, *P* = 0.0079). **l** Serum C4 (*n* = 5 mice per group); unpaired two-tailed Student’s t-test (*Mdr2*^*−/−*^ vs *Mdr2*^*−/−*^+DSS: *P* = 0.0081). All data are graphed as mean ± SEM and considered significant at *p < 0.05, **p < 0.01, ***p < 0.001 and ****p < 0.0001. Source data are provided as a Source Data file.

IBD in PSC patients presents as chronic, widespread, and mild colitis^3,29^. To model the clinical profile of human IBD-PSC more closely, we administered a low dosage of 1 % DSS to 12-week-old *Mdr2*^*−/−*^ mice continuously for 9 weeks. *Mdr2*^*−/−*^ mice showed slower weight gain after initiation of DSS and noticeable weight loss at later time points. However, the magnitude of body weight loss was lower and the colitis symptoms were milder compared to acute colitis induced by a high concentration of DSS (Fig. 6a). Additionally, DSS-treated *Mdr2*^*−/−*^ mice presented signs of colitis with diarrhea, inflamed colon, shortened colon length, and higher spleen to body weight ratio (Fig. 6b, d, Supplementary Fig. 8a).

Consistent with DSS-induced acute colitis, serum biochemical parameters (ALT, AST, AP and GLDH) were significantly decreased in *Mdr2*^*−/−*^ mice after chronic DSS feeding (Fig. 6c). Of note, ALT and AP of some mice were suppressed to the levels of WT controls. In line, caspase 3 activity was remarkably decreased in the *Mdr2*^*−/−*^ DSS mice (Fig. 6e), together with upregulated anti-apoptotic proteins (A1/Bfl-1 and cIAP1) (Fig. 6f, Supplementary Fig. 8b). Most interestingly, biliary septal fibrosis was improved in *Mdr2*^*−/−*^ DSS mice, as evidenced by quantification of Sirius red and collagen I stainings (Fig. 6d, Supplementary Fig. 8c–e). In agreement with histological findings, expression of fibrosis markers (*Col1a1*, *Col1a2*, *Col3a1*) and the activated HSC marker (*Timp1*) were markedly reduced in *Mdr2*^*−/−*^ mice exposed to DSS (Fig. 6g), whereas *αSma* was only mildly decreased and PF markers (*Cd34*, *Thy1*, *Elastin*, *Msln*) were not significantly changed in the treatment group (Supplementary Fig. 8f).

Expression of inflammatory genes (*Icam-1*, *F4/80, Tlr4*, *Il1β*, *Nlrp3*, *Nfkb*, *Ccl5*, *Ifng*, *Il12a*, *Il10*) in the liver of *Mdr2*^*−/−*^ mice was significantly increased after chronic DSS treatment (Supplementary Fig. 8g–i), accompanied by elevated protein levels of phosphorylated NF-κB P65 and nucleus translocation in hepatocytes (Fig. 6h, Supplementary Fig. 8j–k). Conversely, mRNA levels of genes related to bile acid synthesis and transport (*Cyp7a1*, *Cyp27a1*, *Oatp2*, *Mrp2*) were strongly decreased while liver FXR signaling (*Fxr*, *Shp*) remained unaffected (Fig. 6i).

Consequently, DSS-induced chronic colitis led to decreased levels of total bile acids in the liver (Fig. 6j) and plasma collected from vena cava or portal vein (Fig. 6k). Furthermore, C4 was substantially reduced in *Mdr2*^*−/−*^ DSS mice (Fig. 6l), thereby confirming that the improved biochemical and pathological phenotype of *Mdr2*^*−/−*^ DSS mice might be explained by inhibition of bile acid synthesis.

Taken together, these findings suggest that chronic colitis-triggered liver inflammation reduces cholestatic liver injury and biliary septal fibrosis via suppression of bile acid synthesis.

### Intestinal inflammation is associated with longer liver transplantation-free survival in PSC patients

**Fig. 7:**
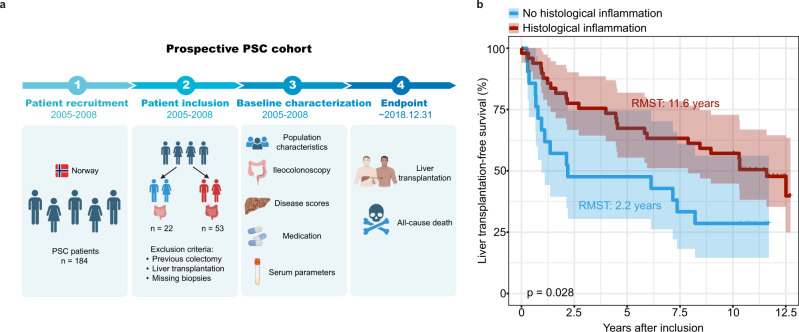
Histological inflammation of intestine improves the liver transplantation-free survival in PSC patients. **a** Diagram of the study design for the PSC cohort. **b** Kaplan–Meier survival curves of PSC patients according to the presence of histological inflammation of intestine. (Histological inflammation: *n* = 49; No histological inflammation: *n* = 21; 4 patients with histological inflammation and 1 patient without were excluded to avoid bias caused by patients already being listed for a liver transplantation since they reached endpoint within 3 months from inclusion). Survival probabilites for each group are shown using the Kaplan-Meier estimator with corresponding (coloured) 95% confidence intervals and the log-rank test (P = 0.028). Average survival times are shown in figure using restricted mean survival time (RMST). Source data are provided as a Source Data file.

To further strengthen the translational link, we analyzed the association between intestinal inflammation and PSC transplant free survival in a previously described well-characterized prospective PSC cohort^30^. A set of 70 non-colectomized PSC patients underwent ileocolonoscopy at a single time point between 2005 and 2008 with biopsies from several segments (from rectum to terminal ileum). Biopsies were thoroughly assessed by the same pathologist for inflammation. The presence of histological inflammation in any segment (terminal ileum, ascending, descending or sigmoid colon) was associated with reduced risk of liver transplantation or death (Fig. 7a, b), even after correcting for the Amsterdam-Oxford risk score for PSC (multivariate cox proportional hazards model, HR: 0.52, 95% CI 0.27–0.99, *p* = 0.047)^31^. Moreover, the Kaplan-Meier plot analysis showed that the severity of histological inflammatory activity (graded as no inflammation, mild inflammation, moderate and severe inflammation) was associated with different survival estimates, with worse outcomes observed in the no inflammation group (Supplementary Fig. 9). Regarding confounding parameters different between the inflammation and non-inflammation groups (Supplementary Table 1), the Amsterdam-Oxford score takes into account both age at diagnosis and key biochemical parameters. Prednisone use was also different in the groups but did not have a significant impact on the patient’s outcome in a univariate model (HR 1.03, 95% CI 0.48–2.23, *p* = 0.93).

Mayo PSC score, FIB-4 score and Bilirubin were reduced, and trends existed towards decreased AOM score in PSC patients with histological inflammation compared to those without (Supplementary Table 1). These data suggest that histological inflammation is associated with longer transplantation-free survival supporting a potential protective role against disease progression. However, future prospective and sufficiently powered clinical studies are required to validate this hypothesis.

## Discussion

Increasing evidence suggests an intimate relationship between primary sclerosing cholangitis and inflammatory bowel disease^32,33^. Whereas experimental data indicate that unfavorable microbiota and barrier impairment may promote or even cause sclerosing cholangitis^10,11,34–36^, epidemiological data are less clear with some studies pointing towards a liver disease-promoting role of IBD and others indicating that active IBD may even delay the progression of PSC^15,16,37–40^.

Colitis and barrier dysfunction have been demonstrated to elicit negative impacts on numerous diseases of the liver and other organ systems^11,41–43^. In contrast, our IBD-PSC mouse model uncovers a protective effect of colitis on liver injury in *Mdr2*^*−/−*^ mice. We found that local intestinal inflammation triggered by DSS induced major changes in hepatic transcriptomic programs with upregulation of inflammatory pathways and suppression of bile acid synthesis and transport. The mRNA expression of liver *Cyp7a1, Cyp27a1, Ntcp, Oatp2, Mrp2* was downregulated in both WT and *Mdr2*^*−/−*^ mice after DSS treatment. Hence, the colitis-induced inhibition of genes responsible for bile acid synthesis and transporters is not *Mdr2*^*−/−*^ specific but occurs in WT animals as well. However, except for C4 levels, total bile acids in different compartments (serum, portal serum, liver and cecum stool) did not differ between WT and WT DSS groups, whereas *Mdr2*^*−/−*^ DSS mice exhibited a significant reduction of bile acids compared with *Mdr2*^*−/−*^ controls, hereby improving cholestatic liver injury. Our data demonstrate that in *Mdr2*^*−/−*^ mice increased bile acid synthesis results in accumulation of bile acids in liver tissue and eventually spillover in the systemic circulation as evidenced by MALDI imaging as well as measurement of bile acid concentrations in tissue and serum samples, which could be counteracted by DSS treatment. This does not happen in WT mice due to the absence of cholestasis. However, contrary to our findings, Zhou et al.^44^ found increased levels of Cyp7A1, which was linked to reduced ileal FGF15 expression. Since this is an important mechanism of gut-liver crosstalk in cholestatic liver disease, we have studied this axis in detail. Importantly, our experiments did not reveal differences in ileal FGF15 expression or its target genes. This is very likely due to the strong differences between the experimental setups used in the study of Zhou et al. and our study. Zhou et al. induced colitis by administration of 2.5% (w/v) DSS in drinking water for 7 days followed by a 14-day washout with drinking water for in total 3 cycles. In our study, DSS was administered continuously either for 7 days with 2.5% (w/v) or for 9 weeks at a concentration of 1%. We did not include a washout phase or different cycles of DSS treatment. Our data are in line with previous results showing that colitis and severe intestinal barrier impairment cause systemic translocation of MAMPs^45^. Since the liver receives 2/3 of its blood supply from the intestine, it is strongly affected by these influences^46^. Consistent with what was observed in endotoxin-induced cholestasis^25,47–49^, in-vivo LPS administration profoundly suppressed hepatobiliary genes. Our data extend on these findings by demonstrating how LPS affects pre-existing cholestatic liver injury. Contrary to minor hepatocellular damage seen in sepsis-associated cholestasis^50^, LPS-injected *Mdr2*^*−/−*^ mice mimicked the phenotype of our colitis model, which is triggered by barrier impairment and translocation of MAMPs. LPS treatment was sufficient to protect against cholestatic liver injury.

DSS-induced chronic colitis attenuated biliary septal fibrosis, which may be attributed to less BA accumulation mediated inhibition of activated HSCs (*Col1a1*, *Col1a2*, *Col3a1* and *Timp1*) without affecting PFs in *Mdr2*^*−/−*^ mice. This is in line with previous publication showing that activated HSCs are mostly localized in the sinusoidal and capsular regions, while PFs are mainly located in the portal areas where neutrophils migrate into during the early stage of intestinal barrier impairment. Activated HSCs demonstrate a stronger fibrogenic phenotype than PFs, thereby acting as a major source of extracellular matrix in *Mdr2*^*−/−*^ mice^28^.

Together our study has several important implications. It highlights hepatocellular NF-κB as a double-edged sword in the cholestatic liver. While inflammatory gene expression may fuel liver injury and disease progression, hepatocytic NF-κB signaling protects *Mdr2*^*−/−*^ mice from severe cholestatic liver damage via anti-apoptotic gene expression^20,51^. However, relatively little is known about the involvement of bile acid metabolism in this process. Here, we observed a strong inverse correlation between inflammatory gene expression and genes involved in bile acid synthesis and transport. In sepsis-induced cholestasis, TNFα was the driving force to alter BA metabolism^52,53^. Since this cytokine was also strongly induced in DSS-treated *Mdr2*^*−/−*^ mice, we hypothesized that TNFα released from cells in the hepatic inflammatory microenvironment may trigger a protective NF-kappaB-mediated program in hepatocytes. In line with this hypothesis, *Mdr2*^*−/−*^ mice lacking hepatic NF-κb signaling (*Mdr2*^*−/−*^*Nemo*^*Δhepa*^) demonstrated aggravated cholestatic liver disease even in the absence of colitis. However, TNFα neutralization neither exacerbated liver injury in *Mdr2*^*−/−*^ DSS mice nor restored the DSS-induced suppression of BA metabolism-associated genes (Supplementary Fig. 10). Since translocation of bacterial components induced various pattern recognition receptors (PRRs) and inflammatory cytokines, it is likely that there are redundant pathways, which result in hepatic NF-κB activation as the most downstream event mediating the protective phenotype induced by colitis.

Additionally, our study offers a new perspective on the pathophysiology of PSC. Its auto-immune inflammatory nature suggests that immunosuppressive or anti-inflammatory treatments might improve cholestatic liver disease. However, to date, all anti-inflammatory PSC treatments have failed^54^. In a recent trial, Vedolizumab, which blocks the integrin α4β7 and has been used successfully to treat patients with IBD, has even worsened cholestatic liver disease reflected by increased serum alkaline phosphatase and liver transaminase levels^55^. Interestingly, only a subset of patients with cirrhosis showed a biochemical response to Vedolizumab. In previous work, we have shown that patients with advanced disease and cirrhosis are more likely to have suppressed BA synthesis^18^. Thus, we speculate that these cirrhotic patients don’t rely on inflammation-mediated suppression of bile acid synthesis since they suffer from very poor hepatocyte functions (e.g., BA synthesis). In this case, however, our animal models are at a much earlier stage of the disease spectrum where BA synthesis is still active and contributes to disease progression. Therefore, our preclinical data raise the provocative question of whether colitis and bacterial translocation in early-stage PSC patients with maintained BA synthesis may even protect from cholestasis and disease progression. Do certain PSC patients rely on intestinal inflammation to suppress BA synthesis? Consistent with what we observed in *Mdr2*^*−/−*^ DSS mice, we provide first clinical evidence that intestinal inflammation might be associated with increased transplantation-free survival in PSC patients. Moreover, a recently published paper showed a negative correlation between genes related to BA metabolism and inflammation in human PSC, primary biliary cholangitis (PBC) and autoimmune hepatitis (AIH) using RNA sequencing analysis of liver biopsies^52^, which aligns with our findings that colitis-induced liver inflammation inhibited bile acid metabolism in *Mdr2*^*−/−*^ mice. Whilst these data and some epidemiological evidence^15,16^ suggest that similar mechanisms are relevant in human PSC, further clinical studies are clearly warranted.

Finally, as a consequence, our current data could provide an explanation for why so many PSC treatments have failed: Anti-inflammatory treatments may release the NF-κB-mediated break on bile acid synthesis and therefore aggravate cholestatic liver injury. If this concept can be confirmed in human PSC, future treatments should combine anti-inflammatory agents with drugs that suppress BA synthesis (e.g. FXR agonists).

Some technical and conceptual limitations of this study need to be mentioned. First, our IBD-PSC animal model demonstrates serious colitis predominantly in the distal colon, which does not perfectly mimic the distinct intestinal phenotype of human IBD-PSC. An improved animal model should be established in the future. Second, our current experiments do not dissect whether gut-derived bacterial components exert their effect on hepatocytes directly or indirectly via immune cells in the hepatic microenvironment. In line with the latter concept, we observed a strong infiltration of neutrophils in DSS or LPS treated *Mdr2*^*−/−*^ mice, which could be the source of inflammatory mediators that act on hepatocytes. It is likely that various inflammatory mediators, directly and indirectly, act on hepatocytes to activate NF-κB as the most downstream event. Further, our current study does not address potential NF-κB-independent functions of *NEMO* in *Mdr2*^*−/−*^ mice, which could contribute to the phenotype as well. Finally, most conclusions in our study are based on murine data.

Our in-vitro and prospective IBD-PSC cohort indicate that similar mechanisms might be relevant in humans, which is also supported by two previous studies suggesting a protective role of IBD in PSC^15,16^. In our PSC cohort, intestinal inflammation was only assessed at a single time point, whereas ideally the relationship between the gut and liver disease should be studied with multiple observations longitudinally to quantify the overall exposure of the liver to intestinal inflammation. Hence, further assessments in well-balanced and larger prospective studies are warranted to firmly establish the proposed concept and dissociate the interactions between inflammation and bile acid metabolism in patients with early-stage and advanced PSC.

In conclusion, our study uncovers an unexpected context in which intestinal barrier impairment is protective, offers a new perspective on the long-standing IBD-PSC association and provides a strong rationale for clinical research into multi-organ treatment strategies for PSC (Supplementary Fig. 11).

## Methods

### Mice

All animals were housed at a temperature of 21−23 °C with relative humidity of 35–65% and under specific pathogen free conditions of 12 h:12 h light-dark cycle in individually ventilated cages with standard chow diet (ssniff #V1534-300) and water ad libitum. All in vivo experiments were repeated independently at least twice by using littermates. At the end of each experiment, all mice were euthanized under general anaesthesia using isoflurane. All experimental procedures were performed according to the *Guide for the Care and Use of Laboratory Animals* and approved by the local authorities (LANUV, Germany; no. AZ-84-02.04. 2017.A327 (C.T.), no. AZ-81-02.04. 2020.A033(C.T.), no. AZ-81-02.04. 2022.A230(C.T.))

### PSC cohort

As described in a previous publication^30^, 184 liver transplanted and non-transplanted PSC patients were prospectively recruited at Rikshospitalet (Oslo, Norway) between 2005 and 2008, and underwent ileocolonoscopy. Cause of referral was follow-up or confirmation of PSC. Patients with previous colectomy, liver transplantation or missing biopsies were excluded, leaving 70 PSC patients eligible for time-to-event analysis. Biopsies from at least 2 out of 4 segments were available for histopathological evaluation (terminal ileum, ascending, descending and sigmoid colon). Histological inflammation was defined as any degree of mucosal inflammation present in at least 1 segment. Diagnosis of PSC was done according to clinical guidelines^56^. Biochemistry (patients records) was obtained at inclusion, and date of death (Norwegian cause of death registry, FHI, Oslo, Norway) and liver transplantation (Nordic Liver Transplant Registry) was collected up until the 31st of December 2018. Liver transplantation-free survival was defined as time from inclusion until liver transplantation or all-cause death. Patients that reached endpoint within 3 months from inclusion, were excluded from survival analysis to avoid bias caused by patients already being listed for a liver transplantation. Cox proportional hazards models, Kaplan-Meier analysis and survival plots was generated in R and Quarto using the survival and survminer packages (R studio version 2022.07.02). This human study was approved by the Regional Committee for Medical and Health Research Ethics (projects 2015/2140 and 2016/1690) at Rikshospitalet (Oslo, Norway) and all included patients gave informed consent.

### DSS induction of acute and chronic colitis

To establish acute colitis, littermates of 8–10-week-old WT and *Mdr2*^*−/−*^ (FVB/N) male mice were randomly allocated to experimental or control groups. Subsequently, mice were treated with 2.5% dextran sodium sulfate (DSS; MW 36,000-50,000, MP Biomedicals, S2839) or drinking water as control for 7 days. Body weight was recorded every day.

For inducing chronic colitis, littermates of 12-week-old male *Mdr2*^*−/−*^ (FVB/N) mice were randomly assigned to experimental or control groups. Next, mice received 1% DSS or drinking water for 9 consecutive weeks, and body weight was documented weekly.

In both acute and chronic DSS model, freshly prepared DSS was replaced every second day.

### Treatment with lipopolysaccharide

To investigate the role of liver inflammation on cholestatic liver injury, 8-week-old *Mdr2*^*−/−*^ (FVB/N) mice were injected intraperitoneally with 5 mg/kg Lipopolysaccharide (LPS, from Escherichia Coli O127:B8, Sigma, L3129). The littermate control mice were injected with the same volume of phosphate-buffered saline (PBS). After 16 h, mice were sacrificed for analysis.

### Generation of *Mdr2*^*−/−*^*Nemo*^*Δhepa*^ mice

Albumin-cre transgenics were crossed with C57BL/6 mice bearing loxP-flanked *NEMO* alleles to obtain mice with hepatocyte-specific deficiency of *NEMO* (*Nemo*^*Δhepa*^). *Mdr2*^*−/−*^*Nemo*^*Δhepa*^ double-knockout mice were then generated by crossbreeding *Mdr2*^*−/−*^ mice (C57Bl6/J background) with *Nemo*^*Δhepa*^. *Mdr2*^*−/−*^*Nemo*^*f/f*^ mice were used as control. To investigate the functional role of NEMO-mediated NF-κB signaling, 9–14-week-old male *Mdr2*^*−/−*^*Nemo*^*f/f*^ as well as *Mdr2*^*−/−*^*Nemo*^*Δhepa*^ mice were sacrificed for further analysis due to low reproductive potential and early spontaneous death.

### Injection with Infliximab

To explore whether TNFα was the mediator to induce DSS-triggered inhibition of bile acid metabolism, littermates of 8–10-week-old *Mdr2*^*−/−*^ (FVB/N) mice were treated with 2.5% DSS for 9 days. From day 6 to day 8, *Mdr2*^*−/−*^ mice were injected with 10 mg/kg infliximab (Remicade, 06G233821LA) or PBS daily for 3 consecutive days. At day 9, mice were sacrificed for analysis. Fresh DSS solution was replaced every second day, and body weight was recorded every day.

### GEO dataset acquisition

To explore the effect of inflammation on bile acid metabolism in human hepatocytes, we downloaded microarray data from Gene Expression Omnibus (GEO) database by the accession number of GSE104601^57^. In this dataset, J. Jiang et al. stimulated co-cultured primary human hepatocytes and Kupffer cells (3-dimentional Human Liver Microtissues) with 10 μg/ml LPS. After 24 h, the cells were harvested by J. Jiang et al. for RNA isolation and microarray analysis.

### H&E and Sirius red staining

Colon with fecal pellets and liver tissues were fixed in Carnoy’s fixative (10% acetic acid, 30% chloroform, 60% methanol), or 4% formaldehyde solution respectively for 24 h, followed by paraffin embedding. Sections were cut (2 μm), deparaffinized and rehydrated. For H&E staining, the hematoxylin solution was applied to the rehydrated sections for 5 min, followed by a rinse with running tap water for 5 min. Then the slides were submerged in Eosin solution for 2 min and subsequently processed with distilled water shortly. In the end, sections were dehydrated through a series of ethanol with increasing concentration and xylol.

To perform Sirius red staining, rehydrated sections were immersed in 0.1% Sirius red solution for 1 h. The samples were then incubated twice in 0.5% glacial acetic acid for 15 s each. Lastly, the tissue sections were dehydrated in the same way as for H&E staining. Images were obtained with AxioVision 4.9 (Zeiss) and positive areas of Sirius red staining were quantified by ImageJ (NIH).

### Histological score assessment of colitis

H&E-stained colonic sections were evaluated by a pathologist as previously described^58^. The severity of inflammation activity was scored as follows (based on neutrophils): 0, no neutrophils in the lamina propria; 1, increased neutrophils in the lamina propria or epithelium; 2: crypt abscess, erosion or ulcer. Chronic injury was scored as follows: 0, no crypt distortion and absence of elevated lymphoplasmacytic cells in the lamina propria; 1, crypt distortion and no band-like lamina propria lymphoplasmacytosis; 2, crypt distortion and band-like lamina propria lymphoplasmacytosis.

### Quantification of liver neutrophils by morphometry

H&E-stained liver slides were assessed by a pathologist. From every section, up to 6 areas (each ROI: 0.3125 mm²) were randomly selected to quantify the infiltrating neutrophils identified by their characteristic morphology. For further statistical analysis, average count of neutrophils per ROI was used.

### Serum biochemistry

Serum samples were diluted with PBS (1:5). Liver injury markers ALT, AP, AST and GLDH were subsequently examined at the central lab of clinical chemistry in University Hospital RWTH Aachen.

### Immunohistochemistry

5 μm paraffin-embedded sections of liver were deparaffinized, rehydrated and then immersed in citrate buffer (pH 6.0) in a steam cooker for antigen unmasking. Subsequently, endogenous peroxidases were blocked via immersing slides in 3% hydrogen peroxide for 10 min. Next, 2,5% goat serum was utilized to block nonspecific binding. Cytokeratin 19 (CK19), Cleaved Caspase 3 and Ki67 antibodies were diluted 1:200 in blocking solution and applied onto the surface of tissue sections overnight at 4 °C in a humidified chamber. Following washing with PBST (PBS, 0.05% Tween 20), slides were incubated with horseradish peroxidase (HRP)-linked secondary antibodies for 2 h at room temperature. DAB detection kit was used to stain HRP and nuclei were counterstained with Mayer’s haematoxylin. Lastly, all stained slides were dehydrated through an increasing ethanol concentration for 2 min each and coverslipped with Roti histokitt. The images were acquired using AxioVision 4.9 (Zeiss), and quantification of targeted signals was performed by ImageJ (NIH).

### Immunofluorescence staining

5 μm tissue-Tek-embedded frozen sections were dried in air for 30 min and fixed with 4% formaldehyde solution for 10 min. Subsequently, slides were blocked with 5% goat serum for 1 h, and incubated with primary antibodies against ZO-1, Collagen I, Ly6G or CD11b (1:200) overnight at 4 °C, followed by washing in PBST. The appropriate fluorochrome-conjugated secondary antibodies (1:400) were added to slices and incubated at room temperature for 1 h in the dark. Finally, the slides were mounted with DAPI to stain nuclei. Prior to incubation with fluorescence-labeled secondary antibody, immunofluorescence staining against MUC2 (1:200) was performed on paraffin-embedded sections by following the same protocol as described for immunohistochemistry.

### Co-staining of HNF4α and TUNEL or phosphorylated P65

5 μm sections of tissue-Tek-embedded frozen liver were dried in air for 30 min and fixed with 4% formaldehyde solution for 10 min. Slides were then incubated in permeabilization solution (0.1% Triton X-100, 0.1% sodium citrate) for 2 min at 4 °C. After washing with PBST, liver sections were incubated with TUNEL reaction mixture (Roche, #11684795910, #11767291910 and #11966006001) in a humidified chamber overnight at 4 °C in the dark according to the manufacturer’s instruction. Following blocking with 50% FCS, 5% BSA, 0.3% Triton X-100 for 1 h at RT, slides were incubated with HNF4α (1:200) antibody overnight at 4 °C in a humidified chamber. Subsequently, donkey anti-goat IgG Alexa 647 (1:400) was added onto the slides and incubated for 1 h at RT. In the end, stained sections were mounted with DAPI.

Regarding the co-staining of HNF4α and phosphorylated P65 (P-P65), the slides were incubated with the antibody mixture (HNF4α and P-P65, 1:200) overnight at 4 °C. After washing with PBST, liver sections were serially incubated with donkey anti-goat IgG Alexa 647 (1:400) and goat anti-rabbit IgG Alexa 488 (1:400) for 1 hour at RT. Other steps were the same as for HNF4α and TUNEL co-staining.

### Real-time quantitative PCR

Multiple studies demonstrated that DSS contamination of RNA extracts effectively prevented successful reverse transcription and cDNA polymerization from colon and other nonenteric organs in DSS treated mice^59,60^. We herein used LiCl purification protocol to remove DSS from RNA preparations as described in ref. ^61^. In brief, total RNA solution was purified by LiCl twice after previous Trizol extraction. Then, sodium acetate and prechilled 100% ethanol were added for RNA precipitation. Following washing with 70% ethanol, RNA pellets were air dried for 10 min and resuspended in DEPC-treated water. RNA concentration and purity were determined by NanoDrop (Thermo Scientific). For reverse transcription of RNA into cDNA, High Capacity cDNA Reverse Transcription Kit was employed in accordance with manufacturer’s specifications. Real-time polymerase chain reaction was carried out with PowerUp SYBR Green Master Mix in QuantStudio Flex system (Applied Bioscience, Thermo Fisher). Relative gene expression was calculated according to the 2^−ΔΔCT^ method. Primer sequences are available in Supplementary Table 2.

### Western blotting

Colon, ileum and liver frozen tissues were homogenized and lysed in NP-40 buffer (50 mM Tris HCl pH 7.5, 150 mM NaCl, 50 mM NaF, 0,5% NP40) or RIPA lysis buffer (25 mM Tris HCl pH 7.6, 150 mM NaCl, 1% NP-40, 1% sodium deoxycholate, 0.1% SDS) with phosphatase and protease inhibitors tablets (PhosSTOP EASY pack, complete Mini, Roche; 1 tablet each/10 ml NP-40 buffer), as well as 0.001 M DTT and 0.001 M pefabloc. Protein concentrations were then tested by using BIO-RAD dye reagent according to the manufacturer’s instruction and 4x laemmli buffer (0.2 M Tris HCl pH 6.8, 40% glycerol, 8% SDS, 0.2% bromophenol blue, 20% 2-Mercaptoethanol) was added to denature proteins at 95 °C for 5 min. Subsequently, 40 μg denatured proteins were electrophoretically separated on pre-cast 4–12% polyacrylamide gel (Bio-Rad) by SDS-PAGE at 140 V for approximately 1 h, and then transferred to nitrocellulose membranes through the Trans-Blot Turbo System (Bio-Rad). Afterwards membranes were blocked with 5% dry milk or BSA and immunoblotted overnight at 4 °C with primary antibodies. Following TBST (1x Tris-Buffered Saline, 0.05% Tween 20) wash and incubation with HRP-conjugated secondary antibodies, immunodetection was performed with ECL substrate and the membranes were exposed by chemiluminescence in the LAS mini 4000 developing machine (Fuji). Protein levels were quantified with ImageJ (NIH) and normalized to GAPDH or β-Actin expression. Detailed information of all antibodies is listed in Supplementary Table 3.

### Flow cytometry of intrahepatic leukocytes

Around 0.3 g fresh liver from the same lobe was chopped into small pieces and digested by collagenase type IV and DNase I at 37 °C for 30 min. The mixture was then filtered through 70 μm cell strainer, proceeded by multiple centrifugation steps as described in ref. ^62^ and removal of erythrocytes with RBC (Red blood cell) lysing buffer. Next, immune cells were divided into two panels and stained with corresponding subset of fluorochrome-conjugated antibodies: 1, myeloid panel: CD45::APC-Cy7, CD11b::V450, Ly6G::AF700, CD11c::APC, MHC II::FITC, F4/80::PE-Cy7, Ly6C::PerCP-Cy5.5, CD80::PE; 2, Lymphoid panel: CD45::APC-Cy7, CD3::APC, CD19::AF700, CD4:: eFluor 450, CD8::FITC, NK1.1::PE-Cy7, TIM3::BV650, CTLA4::PE, PD1:: PE/Dazzle 594, CD25::PERCP-Cy5.5. In the end, labeled cells were acquired by BD FACSDiva 6 on LSRFortessa (BD Biosciences). The data were analyzed by FlowJo software (version 10).

### Quantification of bile acids and C4

Bile acids and 7α-hydroxy-4-cholesten-3-one (C4) were extracted from liver, cecum content, and plasma of vena cava or portal vein by quick methanol extraction as previously published in ref. ^63^. To summarize, internal standards containing methanol were added to samples, followed by homogenization and subsequent centrifugation. Afterwards, the supernatant was fully evaporated under a flux of N_2_ at 40 °C and resuspended in Methanol: H2O (1:1). Gradient elution was then started on Kinetex silica C18 column (100 × 2.1 mm with1.7 µm particles) at 60 °C to separate bile acids. In the end, detection was performed by ultra-performance liquid chromatography tandem mass-spectrometry (UPLC-MS/MS, Wallenberglab laboratory at Sahlgrenska University Hospital, Sweden).

### MALDI-MSI

Liver tissues from the same lobe were frozen-fixed in Tissue-Tek OCT compound. Two adjacent frozen liver sections of 5 μm thickness were then prepared using a Leica CM3050 S cryostat (Leica, Wetzlar, Germany). Next, they were thawed and mounted on IntelliSlides, and then dried in a desiccator. Slides were sprayed (4 layers) with 5 mg/mL 2- mercaptobenzothiazole in acetone/water (5:1) using a HTX imaging sprayer (HTX Technologies LLC, Chapel Hill, NC, USA) at 30 °C and 10 psi nitrogen. Subsequently, one section was processed for MALDI imaging, and the other was stained for CK19 to determine the location of bile ducts. MALDI measurements were performed at the MS1 level using TIMSTOF fleX (Bruker Daltonics, Bremen, Germany) in negative mode without ion-mobility separation and within a mass range of 100–800 m/z. Internal calibration was carried out using 2-mercaptobenzothiazole matrix peaks and taurocholate. Data were interpreted using SCiLS Lab MVS (version 2021c). Immunohistochemistry of CK19 was performed with adjacent frozen section (5 µm). After storage at −80 °C, the slides were stored directly in ice-cold methanol for 3 min, followed by washing in PBS for 5 min. The staining procedure was performed automatically in the autostainer (Discovery Ultra, Roche). We used the Discovery Chromo Map DAB RUO Kit for staining and CK19 antibody was diluted 1:1000 in PBS for immunoreaction. In the end, MALDI picture was overlaid with CK19-stained image to visualize the spatial distribution of TCA signal.

### Caspase 3 activity assay

Frozen liver tissues were homogenized in the lysis buffer (10 mM Hepes pH 7.4, 0.1% Chaps, 2 mM EDTA pH 8.0, 5 mM DTT, 1 mM pefabloc) with protease inhibitors tablets (complete Mini, Roche, 1 tablet each/10 ml lysis buffer). Protein concentrations were then tested by using BIO-RAD dye reagent according to the manufacturer’s instruction. Next, caspase 3 activity was measured by incubating 12.5 μl tissue lysate with 25 μM Ac-DEVD-AFC (Enzo Life Sciences, ALX-260-032-M001) in the reaction buffer (10 mM Pipes pH 7.4, 2 mM EDTA pH 8.0, 0.1% Chaps, 5 mM DTT). Ac-DEVD-AFC was dissolved in DMSO. The release of fluorescent AFC was monitored every 1 h in the dark at 37 °C for 3 h in a fluorescence plate reader (Cytation 3 imaging reader, BioTek) with excitation at 390 nm and emission at 510 nm. The data were caculated as the time-rate of increase in fluorescence (∆fluorescence/hour) after normalizing to the protein concentration and the relative induction was presented.

### FITC-dextran assay

To study the gut permeability of acute colitis in vivo, mice were fasted for 4 h at the end of colitis induction. Subsequently, mice were gavaged with 150 μl of 100 mg/ml Fluorescein isothiocyanate-dextran (FITC-dextran, Sigma, 46944, dissolved in PBS). 4 h later, mice were sacrificed, and blood samples were harvested from the inferior vena cava and kept in the dark at 4 °C immediately. The concentration of FITC-dextran in plasma was measured in a fluorescence plate reader with excitation at 492 nm and emission at 525 nm. The relative induction rate was determined by calculating the fluorescence signal relative to WT controls.

### 16S rDNA gene quantification

As previously described in ref. ^64–67^, microbial DNA was extracted from frozen liver tissues using an optimized tissue-specific technique. Numerous controls were performed both in vitro and in silico to ensure the absence of artefacts such as bacterial DNA contaminants from reagents or non-specific amplification of eukaryotic DNA. To ensure a low background signal from bacterial contamination of reagents and consumables, negative controls consisting of molecular grade water were added in an empty tube separately at the DNA extraction step and amplified and sequenced at the same time as the extracted DNA of the liver samples, as well as added separately at the quantification step. All samples were processed within the same DNA extraction run by the same single experimenter. Specific measures were taken to avoid cross-contamination, such as cleaning and DNA decontamination of the MSPs (safety hoods) in-between each extraction run. Total DNA concentrations were then determined by UV spectroscopy (Nanodrop®, Thermo Scientific).

Real-time PCR amplification was carried out with 16S universal primers (Vaiomer universal 16S primers) aimed at the V3-V4 region of the bacterial 16S ribosomal gene. qPCR steps were executed on a VIIA 7® PCR system (Life Technologies) using Sybr Green technology and the following amplification cycles: hold stage of 10 min at 95 °C, then 40 cycles of 15 s at 95 °C, 1 min at 63 °C and 1 min 72 °C. The total number of bacterial 16S rDNA gene copies present in the samples was measured by qPCR in triplicate and normalized using a plasmid-based standard range (Vaiomer Universal standard plasmids).

### RNA sequencing analysis

Liver total RNA was isolated and purified according to the manufacturer’s protocol of PureLink RNA Mini Kit. Subsequently, RNA concentrations were determined on a Qubit 4 Fluorometer with the RNA BR Assay Kit (Thermo Fisher) and RNA integrity was assessed on a 2100 Bioanalyzer with the RNA 6000 Nano Kit (Agilent Technologies), according to the manufacturers’ instructions. All samples had an RNA integrity value (RIN) > 8 (range 9.2–10.0). Libraries were generated using the TruSeq Stranded mRNA Kit with unique dual indexes (Illumina), according to the manufacturer’s protocol. In brief, poly(A) containing RNA molecules were isolated from 500 ng of total RNA using magnetic beads, fragmented, and first-strand cDNA synthesis was performed using random primers, followed by second-strand cDNA synthesis, incorporating dUTP in place of dTTP to achieve strand specificity. After purification of the double-stranded cDNA fragments with AMPure XP beads (Beckman Coulter) and adenylation of the 3’ ends, unique indexing adapters were ligated to the cDNA, followed by PCR amplification. The final libraries were quantified using the Qubit 1X dsDNA HS Assay Kit (Thermo Fisher) and the library sizes were checked on an Agilent 2100 Bioanalyzer with the DNA 1000 Kit (Agilent Technologies). Each library was paired-end sequenced (2 x 75 bp) using the 500/550 High Output Kit v2.5 (Illumina) on an Illumina NextSeq 550.

For analyzing sequencing files, FastQC (v0.11.9) was used to evaluate the quality of all data. Next, Salmon (v1.8.0) was applied to perform transcript quantification. Further visualisations were carried out in R (version 4.0.3) as previously described in ref. ^68^. Briefly, Salmon quantification results and metadata were imported into R by tximeta package (1.16.1)^69^. Differential gene expression was then analyzed using Deseq2 package (1.38.3)^70^. When adjusted *P* < 0.05, expression differences between groups were considered significant. Subsequently, a two-dimensional PCA clustering map was plotted to compare the global gene expression by using ggplot2 package (3.4.1). Pheatmap (1.0.12) served to graph gene expression heatmap. Gene set enrichment analysis (GSEA) was performed using ClusterProfiler (3.18.1)^71,72^. Spearman correlation was analyzed with Psych package (2.2.9).

### Quantification and statictical analysis

GraphPad Prism 9 (GraphPad Software, La Jolla, CA) was used to perform statistical comparisons. All data are presented as mean ± SEM. Differences between two groups were assessed by unpaired two-tailed Student’s t-test (normal distribution) or two-tailed Mann–Whitney test (non-normal distribution). In cases of more than two groups, significance was analyzed by one-way ANOVA with Bonferroni’s multiple comparison test (normal distribution) or Kruskal–Wallis test with Dunn–Bonferroni test (non-normal distribution). *P* < 0.05 was considered statistical significance indicated as *p* < 0.05 (*), *p* < 0.01 (**), *p* < 0.001 (***) and *p* < 0.0001 (****). Sample sizes were chosen according to the previous experience from our group. Data were excluded only in the case where a technical error occurred during sample preparation. Statistical details of every figure panel, including statistical tests, animal numbers per group, confidence intervals and p value, can be found in the figure legends.

### Reporting summary

Further information on research design is available in the Nature Portfolio Reporting Summary linked to this article.

## Supplementary information

Supplementary Information

Reporting Summary

## Data Availability

In this study, RNA-seq raw data have been uploaded and deposited in the Sequence Read Archive (SRA) database of National Center for Biotechnology Information (NCBI), which becomes publicly accessible via the accession number PRJNA924130. The Gene Expression Omnibus (GEO) dataset of co-cultured primary human hepatocytes and Kupffer cells treated with LPS is available via the accession number GSE104601. The remaining data are available within the article, supplementary information or source data files. Source data are provided with this paper.

## Source data

Source Data

## References

[1] Ponsioen CY (2021). Defining primary sclerosing Cholangitis: Results from an international primary sclerosing cholangitis study group consensus process. Gastroenterology.

[2] Boonstra K, Beuers U, Ponsioen CY (2012). Epidemiology of primary sclerosing cholangitis and primary biliary cirrhosis: A systematic review. J. Hepatol..

[3] Hirschfield GM, Karlsen TH, Lindor KD, Adams DH (2013). Primary sclerosing cholangitis. Lancet.

[4] Loftus EV (2005). PSC-IBD: A unique form of inflammatory bowel disease associated with primary sclerosing cholangitis. Gut.

[5] Dyson JK, Beuers U, Jones DEJ, Lohse AW, Hudson M (2018). Primary sclerosing cholangitis. Lancet.

[6] Barberio B (2021). Prevalence of Primary sclerosing cholangitis in patients with inflammatory bowel disease: A systematic review and meta-analysis. Gastroenterology.

[7] Wijnands AM (2021). Prognostic factors for advanced colorectal neoplasia in inflammatory bowel disease: Systematic review and meta-analysis. Gastroenterology.

[8] Mendes F, Lindor KD (2010). Primary sclerosing cholangitis: Overview and update. Nat. Rev. Gastroenterol. Hepatol..

[9] de Krijger M, Wildenberg ME, de Jonge WJ, Ponsioen CY (2019). Return to sender: Lymphocyte trafficking mechanisms as contributors to primary sclerosing cholangitis. J. Hepatol..

[10] O’Toole A (2012). Primary sclerosing cholangitis and disease distribution in inflammatory bowel disease. Clin. Gastroenterol. Hepatol..

[11] Lora L (1997). Hepatocyte tight-junctional permeability is increased in rat experimental colitis. Gastroenterology.

[12] Eaton JE, Talwalkar JA, Lazaridis KN, Gores GJ, Lindor KD (2013). Pathogenesis of primary sclerosing cholangitis and advances in diagnosis and management. Gastroenterology.

[13] Sasatomi K, Noguchi K, Sakisaka S, Sata M, Tanikawa K (1998). Abnormal accumulation of endotoxin in biliary epithelial cells in primary biliary cirrhosis and primary sclerosing cholangitis. J. Hepatol..

[14] Nordenvall C (2018). Colectomy prior to diagnosis of primary sclerosing cholangitis is associated with improved prognosis in a nationwide cohort study of 2594 PSC-IBD patients. Aliment Pharm. Ther..

[15] Marelli L (2011). Does the severity of primary sclerosing cholangitis influence the clinical course of associated ulcerative colitis?. Gut.

[16] Navaneethan U (2012). Progressive primary sclerosing cholangitis requiring liver transplantation is associated with reduced need for colectomy in patients with ulcerative colitis. Clin. Gastroenterol. Hepatol..

[17] Liao L (2019). Intestinal dysbiosis augments liver disease progression via NLRP3 in a murine model of primary sclerosing cholangitis. Gut.

[18] Schneider KM (2021). Gut microbiota depletion exacerbates cholestatic liver injury via loss of FXR signalling. Nat. Metab..

[19] Schoemaker MH (2003). Resistance of rat hepatocytes against bile acid-induced apoptosis in cholestatic liver injury is due to nuclear factor-kappa B activation. J. Hepatol..

[20] Pikarsky E (2004). NF-kappaB functions as a tumour promoter in inflammation-associated cancer. Nature.

[21] Bisgaard, T. H., Allin, K. H., Keefer, L., Ananthakrishnan, A. N. & Jess, T. Depression and anxiety in inflammatory bowel disease: epidemiology, mechanisms and treatment. *Nat. Rev. Gastroenterol. Hepatol*. 10.1038/s41575-022-00634-6 (2022). 10.1038/s41575-022-00634-6 35732730

[22] Isaacs-Ten A (2020). Intestinal microbiome-macrophage crosstalk contributes to cholestatic liver disease by promoting intestinal permeability in mice. Hepatology.

[23] Vijayvargiya P (2020). Combined fasting serum C4 and primary bile acids from a single stool sample to diagnose bile acid diarrhea. Gastroenterology.

[24] Modica S (2012). Selective activation of nuclear bile acid receptor FXR in the intestine protects mice against cholestasis. Gastroenterology.

[25] Green RM, Beier D, Gollan JL (1996). Regulation of hepatocyte bile salt transporters by endotoxin and inflammatory cytokines in rodents. Gastroenterology.

[26] Luedde T (2007). Deletion of NEMO/IKKgamma in liver parenchymal cells causes steatohepatitis and hepatocellular carcinoma. Cancer Cell.

[27] Moustafa T (2012). Alterations in lipid metabolism mediate inflammation, fibrosis, and proliferation in a mouse model of chronic cholestatic liver injury. Gastroenterology.

[28] Nishio T (2019). Activated hepatic stellate cells and portal fibroblasts contribute to cholestatic liver fibrosis in MDR2 knockout mice. J. Hepatol..

[29] Lunder AK (2016). Prevalence of Sclerosing Cholangitis Detected by Magnetic Resonance Cholangiography in Patients With Long-term Inflammatory Bowel Disease. Gastroenterology.

[30] Jørgensen KK (2012). Inflammatory bowel disease in patients with primary sclerosing cholangitis: Clinical characterization in liver transplanted and nontransplanted patients. Inflamm. Bowel Dis..

[31] de Vries EM (2018). A novel prognostic model for transplant-free survival in primary sclerosing cholangitis. Gut.

[32] Janse M, Lamberts LE, Verdonk RC, Weersma RK (2012). IBD is associated with an increase in carcinoma in PSC irrespective of the presence of dominant bile duct stenosis. J. Hepatol..

[33] Navaneethan U, Venkatesh PG, Lashner BA, Shen B, Kiran RP (2012). The impact of ulcerative colitis on the long-term outcome of patients with primary sclerosing cholangitis. Aliment Pharm. Ther..

[34] Nakamoto N (2019). Gut pathobionts underlie intestinal barrier dysfunction and liver T helper 17 cell immune response in primary sclerosing cholangitis. Nat. Microbiol..

[35] O’Mahony CA, Vierling JM (2006). Etiopathogenesis of primary sclerosing cholangitis. Semin Liver Dis..

[36] Lichtman SN, Keku J, Schwab JH, Sartor RB (1991). Hepatic injury associated with small bowel bacterial overgrowth in rats is prevented by metronidazole and tetracycline. Gastroenterology.

[37] Mueller T (2011). Enhanced innate immune responsiveness and intolerance to intestinal endotoxins in human biliary epithelial cells contributes to chronic cholangitis. Liver Int.

[38] Ngu JH, Gearry RB, Wright AJ, Stedman CA (2011). Inflammatory bowel disease is associated with poor outcomes of patients with primary sclerosing cholangitis. Clin. Gastroenterol. Hepatol..

[39] Wee A, Ludwig J (1985). Pericholangitis in chronic ulcerative colitis: Primary sclerosing cholangitis of the small bile ducts?. Ann. Intern Med.

[40] Rabinovitz M (1990). Does primary sclerosing cholangitis occurring in association with inflammatory bowel disease differ from that occurring in the absence of inflammatory bowel disease? A study of sixty-six subjects. Hepatology.

[41] Numata Y, Tazuma S, Nishioka T, Ueno Y, Chayama K (2004). Immune response in mouse experimental cholangitis associated with colitis induced by dextran sulfate sodium. J. Gastroenterol. Hepatol..

[42] Schneider KM (2015). CX3CR1 is a gatekeeper for intestinal barrier integrity in mice: Limiting steatohepatitis by maintaining intestinal homeostasis. Hepatology.

[43] Gabele E (2011). DSS induced colitis increases portal LPS levels and enhances hepatic inflammation and fibrogenesis in experimental NASH. J. Hepatol..

[44] Zhou X (2014). PPARalpha-UGT axis activation represses intestinal FXR-FGF15 feedback signalling and exacerbates experimental colitis. Nat. Commun..

[45] Batra A (2012). Mesenteric fat - control site for bacterial translocation in colitis?. Mucosal. Immunol..

[46] Balmer ML (2014). The liver may act as a firewall mediating mutualism between the host and its gut commensal microbiota. Sci. Transl. Med..

[47] Vos TA (1998). Up-regulation of the multidrug resistance genes, Mrp1 and Mdr1b, and down-regulation of the organic anion transporter, Mrp2, and the bile salt transporter, Spgp, in endotoxemic rat liver. Hepatology.

[48] Trauner M, Arrese M, Lee H, Boyer JL, Karpen SJ (1998). Endotoxin downregulates rat hepatic ntcp gene expression via decreased activity of critical transcription factors. J. Clin. Invest.

[49] Feingold KR, Spady DK, Pollock AS, Moser AH, Grunfeld C (1996). Endotoxin, TNF, and IL-1 decrease cholesterol 7 alpha-hydroxylase mRNA levels and activity. J. Lipid Res..

[50] Vos TA (1997). Differential effects of nitric oxide synthase inhibitors on endotoxin-induced liver damage in rats. Gastroenterology.

[51] Ehlken H (2011). Hepatocyte IKK2 protects Mdr2−/− mice from chronic liver failure. PLoS One.

[52] Glaser F (2019). Liver infiltrating T cells regulate bile acid metabolism in experimental cholangitis. J. Hepatol..

[53] Miyake JH, Wang SL, Davis RA (2000). Bile acid induction of cytokine expression by macrophages correlates with repression of hepatic cholesterol 7alpha-hydroxylase. J. Biol. Chem..

[54] Chapman MH (2019). British society of gastroenterology and UK-PSC guidelines for the diagnosis and management of primary sclerosing cholangitis. Gut.

[55] Lynch KD (2020). Effects of Vedolizumab in patients with primary sclerosing cholangitis and inflammatory bowel diseases. Clin. Gastroenterol. Hepatol..

[56] Karlsen TH, Folseraas T, Thorburn D, Vesterhus M (2017). Primary sclerosing cholangitis - a comprehensive review. J. Hepatol..

[57] Jiang J (2019). Human 3D multicellular microtissues: An upgraded model for the in vitro mechanistic investigation of inflammation-associated drug toxicity. Toxicol. Lett..

[58] Lang-Schwarz C (2021). Maximizing the diagnostic information from biopsies in chronic inflammatory bowel diseases: recommendations from the Erlangen International Consensus Conference on Inflammatory Bowel Diseases and presentation of the IBD-DCA score as a proposal for a new index for histologic activity assessment in ulcerative colitis and Crohn’s disease. Virchows Arch..

[59] Krych L (2018). Have you tried spermine? A rapid and cost-effective method to eliminate dextran sodium sulfate inhibition of PCR and RT-PCR. J. Microbiol Methods.

[60] Oldak B, Cruz-Rivera M, Flisser A, Mendlovic F (2018). RNA Purity, Real-Time PCR sensitivity, and colon segment influence mRNA relative expression in murine dextran sodium sulfate experimental colitis. J. Biomol. Tech..

[61] Viennois, E., Tahsin, A. & Merlin, D. Purification of Total RNA from DSS-treated Murine Tissue via Lithium Chloride Precipitation. *Bio. Protoc.* **8**, 10.21769/BioProtoc.2829 (2018). 10.21769/BioProtoc.2829 PMC6017984 29951571

[62] Karlmark KR (2009). Hepatic recruitment of the inflammatory Gr1+ monocyte subset upon liver injury promotes hepatic fibrosis. Hepatology.

[63] Tremaroli V (2015). Roux-en-Y Gastric bypass and vertical banded gastroplasty induce long-term changes on the human gut microbiome contributing to fat mass regulation. Cell Metab..

[64] Schneider KM (2022). Imbalanced gut microbiota fuels hepatocellular carcinoma development by shaping the hepatic inflammatory microenvironment. Nat. Commun..

[65] Lelouvier B (2016). Changes in blood microbiota profiles associated with liver fibrosis in obese patients: A pilot analysis. Hepatology.

[66] Lluch J (2015). The Characterization of novel tissue microbiota using an optimized 16S metagenomic sequencing pipeline. PLoS One.

[67] Paisse S (2016). Comprehensive description of blood microbiome from healthy donors assessed by 16S targeted metagenomic sequencing. Transfusion.

[68] Love MI, Anders S, Kim V, Huber W (2015). RNA-Seq workflow: Gene-level exploratory analysis and differential expression. F1000Res..

[69] Love MI (2020). Tximeta: Reference sequence checksums for provenance identification in RNA-seq. PLoS Comput. Biol..

[70] Love MI, Huber W, Anders S (2014). Moderated estimation of fold change and dispersion for RNA-seq data with DESeq2. Genome Biol..

[71] Yu G, Wang LG, Han Y, He QY (2012). clusterProfiler: An R package for comparing biological themes among gene clusters. OMICS.

[72] Stephens M (2017). False discovery rates: A new deal. Biostatistics.

