# Supplementary material for: Colitis ameliorates cholestatic liver disease via suppression of bile acid synthesis
Source: Nat Commun. 2023 Jun 6;14:3304. doi: 10.1038/s41467-023-38840-8 (PMC10244448; doi:10.1038/s41467-023-38840-8)
Supplement: Supplementary file 1 — Supplementary Information [file 41467_2023_38840_MOESM1_ESM.pdf]

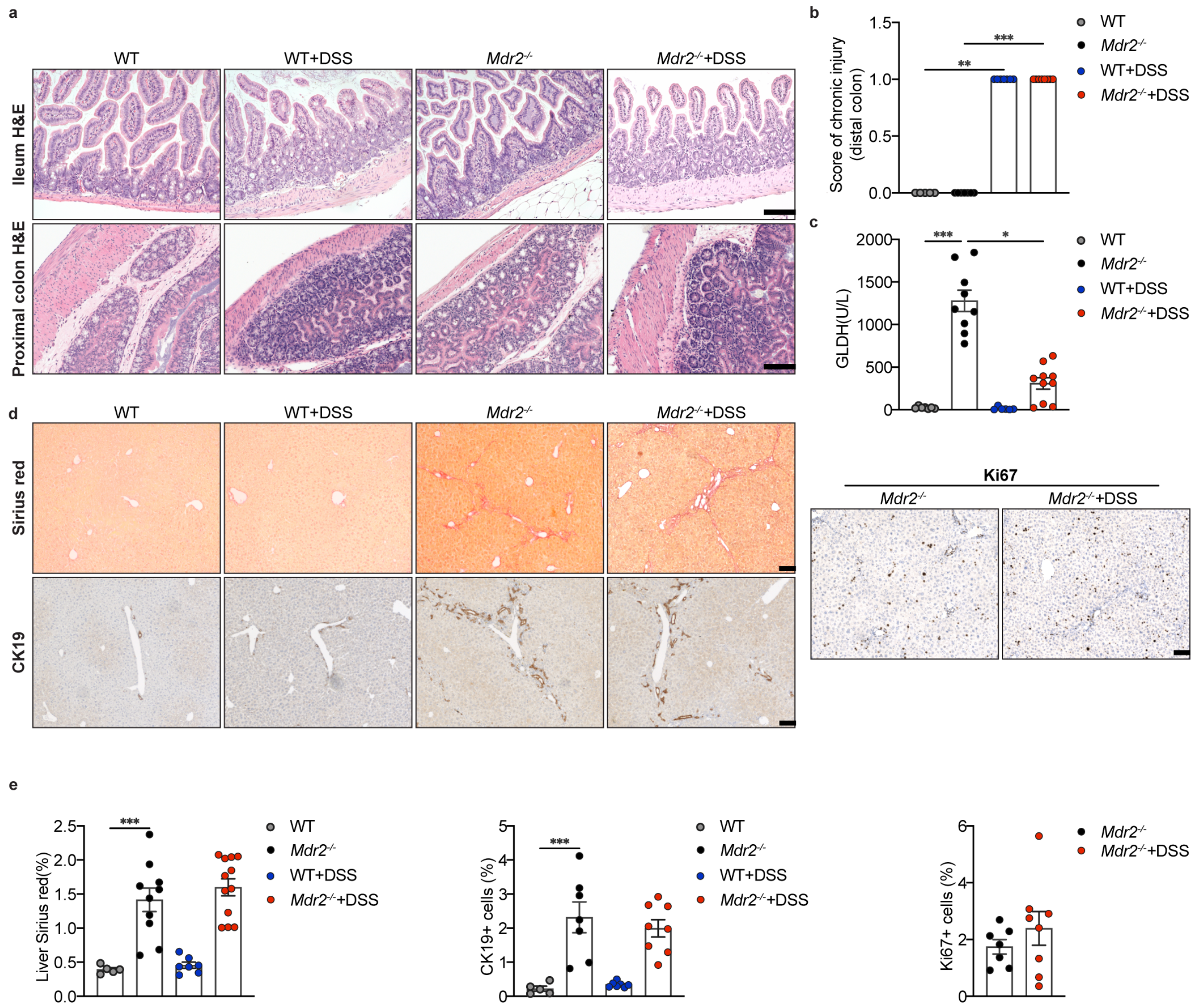

Supplementary Fig. 1 Effects of DSS induced acute colitis on cholestatic liver disease in *Mdr2*$^{-/-}$ mice. a H&E staining of ileum and proximal colon sections (scale bar, 100 μm) (WT, n = 5;*Mdr2*$^{-/-}$,n = 8; WT+DSS, n = 6; *Mdr2*$^{-/-}$+DSS, n = 8). **b** Histological scoring of chronic colon injury (WT, n = 5; *Mdr2*$^{-/-}$,n = 7; WT+DSS, n = 7; *Mdr2*$^{-/-}$+DSS, n = 8); Kruskal-Wallis test with Dunn's multiple comparison test (WT vs WT+DSS, P = 0.003; *Mdr2*$^{-/-}$ vs *Mdr2*$^{-/-}$+DSS, P = 0.0005). **c** Serum GLDH levels (WT, n = 7; *Mdr2*$^{-/-}$,n = 9; WT+DSS, n = 6; *Mdr2*$^{-/-}$+DSS, n = 10) from 2 representative independent cohorts. Kruskal-Wallis test with Dunn's multiple comparison test (GLDH: WT vs *Mdr2*$^{-/-}$, P = 0.0002; *Mdr2*$^{-/-}$ vs *Mdr2*$^{-/-}$+DSS, P = 0.0359). **d** Representative images of liver sections stained by Sirius red (WT, n = 5; *Mdr2*$^{-/-}$,n = 10; WT+DSS, n = 7; *Mdr2*$^{-/-}$+DSS, n = 12), CK19 (WT, n = 5; *Mdr2*$^{-/-}$,n = 7; WT+DSS, n = 7; *Mdr2*$^{-/-}$+DSS, n = 8) and Ki67(*Mdr2*$^{-/-}$,n = 7; *Mdr2*$^{-/-}$+DSS, n = 8) (scale bar, 100 μm). **e** Quantification analysis of liver sections stained by Sirius red (WT, n = 5; *Mdr2*$^{-/-}$,n = 10; WT+DSS, n = 7; *Mdr2*$^{-/-}$+DSS, n = 12), CK19 (WT, n = 5; *Mdr2*$^{-/-}$,n = 7; WT+DSS, n = 7; *Mdr2*$^{-/-}$+DSS, n = 8) and Ki67(*Mdr2*$^{-/-}$,n = 7; *Mdr2*$^{-/-}$+DSS, n = 8); one-way ANOVA with Bonferroni's multiple comparison test (Sirius red: WT vs *Mdr2*$^{-/-}$, 95% CI -1.608 to -0.4371, P = 0.0003; CK19: WT vs *Mdr2*$^{-/-}$, 95% CI -3.256 to -0.9273, P = 0.0003); unpaired two-tailed Student's t-test (Ki67: *Mdr2*$^{-/-}$ vs *Mdr2*$^{-/-}$+DSS, no significant difference). All data are displayed as mean ± SEM and considered statistically significant at $p < 0.05$ (*), $p < 0.01$ (**), $p < 0.001$ (***) and $p < 0.0001$ (****). Source data are provided as a Source Data file.

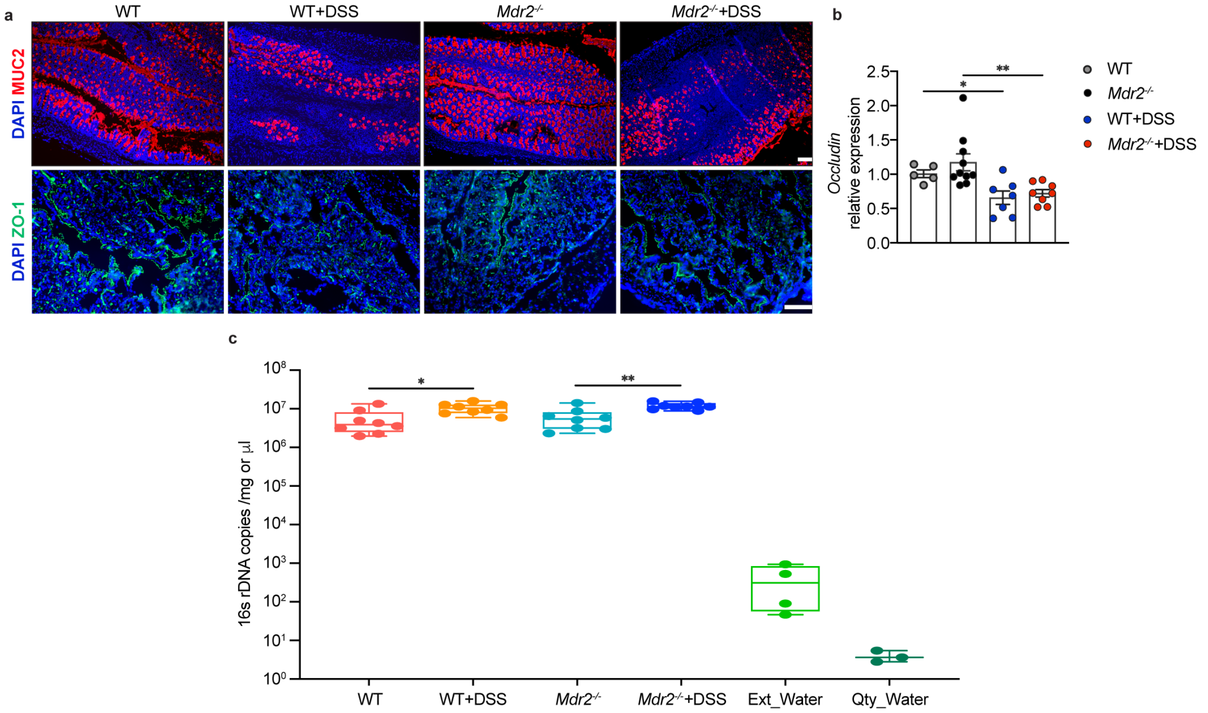

Supplementary Fig. 2 DSS-induced acute colitis promotes gut barrier dysfunction and translocation of bacterial components in both WT and *Mdr2*$^{-/-}$ mice. a Representative MUC2 and ZO-1 immunofluorescence pictures from colon sections (MUC2: WT, n = 5; *Mdr2*$^{-/-}$,n = 6; WT+DSS, n = 7; *Mdr2*$^{-/-}$+DSS, n = 8; ZO-1: WT, n = 4; *Mdr2*$^{-/-}$,n = 7; WT+DSS, n = 5; *Mdr2*$^{-/-}$+DSS, n = 4) (scale bar, 100 µm). **b** Colon mRNA expression level of tight junction marker *Occludin*(WT, n = 5; *Mdr2*$^{-/-}$,n = 10; WT+DSS, n = 7; *Mdr2*$^{-/-}$+DSS, n = 8); Kruskal-Wallis test with Dunn's multiple comparison test (WT vs WT+DSS, P = 0.0304; *Mdr2*$^{-/-}$ vs *Mdr2*$^{-/-}$+DSS, P = 0.0038). **c** Quantity of 16s rDNA gene copies per mg of the liver tissue in acute DSS-treated WT and *Mdr2*$^{-/-}$ mice (n = 8 mice per group), and 16s rDNA gene copies per µl of the negative controls (extraction with molecular grade water (Ext_Water) as starting material, n = 4; quantification with molecular grade water (Qty_Water) as starting material, n = 3); one-way ANOVA with Bonferroni's multiple comparison test (WT vs WT+DSS, 95% CI -9700599 to -601901, P = 0.0212; *Mdr2*$^{-/-}$ vs *Mdr2*$^{-/-}$+DSS, 95% CI -10300599 to -1201901, P = 0.0087). The box-and-whisker plot shows the distribution of each set of data, with the whiskers extending to the minimum and maximum values and the boxes spanning from the first quartile (25th percentile) to the third quartile (75th percentile) of the data. The centre line of the box represents the median value of the group.. All data are displayed as mean ± SEM and considered statistically significant at $p < 0.05$ (*), $p < 0.01$ (**), $p < 0.001$ (***) and $p < 0.0001$ (****). Source data are provided as a Source Data file.

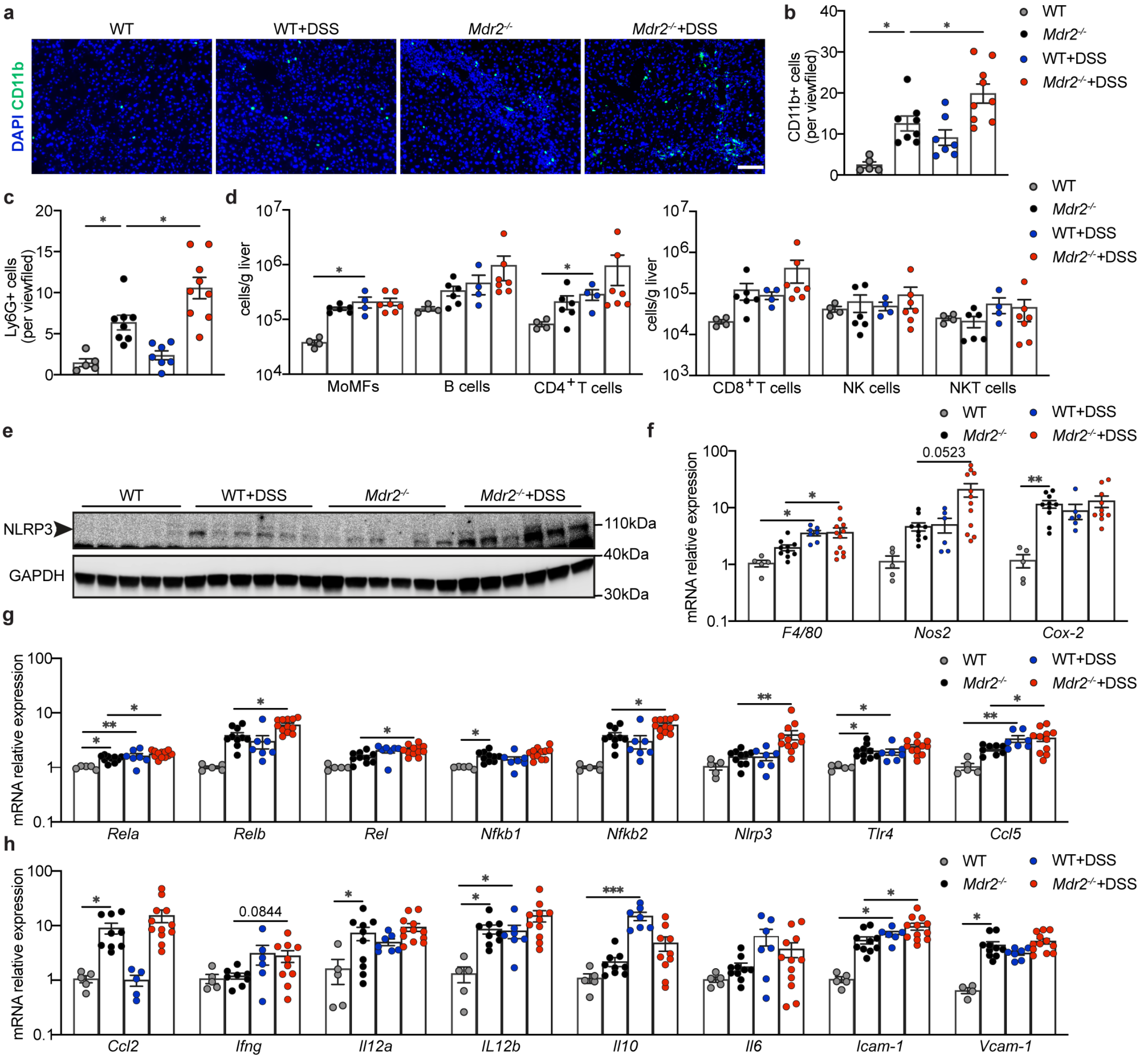

Supplementary Fig. 3 DSS-induced acute colitis enhances hepatic inflammation in *Mdr2*$^{-/-}$ mice. **a** Representative images and **b** quantification of hepatic CD11b staining (WT, n = 5; *Mdr2*$^{-/-}$,n = 8; WT+DSS, n = 7; *Mdr2*$^{-/-}$+DSS, n = 9) (scale bar, 100 µm); one-way ANOVA with Bonferroni's multiple comparison test (WT vs *Mdr2*$^{-/-}$, 95% CI -18.38 to -1.758, P = 0.0128; *Mdr2*$^{-/-}$ vs *Mdr2*$^{-/-}$+DSS, 95% CI -14.38 to -0.2079, P = 0.0416). **c** Quantification of Ly6G staining in liver sections (WT, n = 5; *Mdr2*$^{-/-}$,n = 8; WT+DSS, n = 7; *Mdr2*$^{-/-}$+DSS, n = 9); one-way ANOVA with Bonferroni's multiple comparison test (WT vs *Mdr2*$^{-/-}$, 95% CI -9.031 to -0.7423, P = 0.0159; *Mdr2*$^{-/-}$ vs *Mdr2*$^{-/-}$+DSS, 95% CI -7.739 to -0.7727, P = 0.0161). **d** Numbers of liver infiltrating MoMFs, B cells, CD4$^+$ T cells, CD8$^+$ T cells, NK cells and NKT cells (WT, n = 4; *Mdr2*$^{-/-}$,n = 6; WT+DSS, n = 4; *Mdr2*$^{-/-}$+DSS, n = 7); Kruskal-Wallis test with Dunn's multiple comparison test (MoMFs: WT vs WT+DSS, P = 0.0207; CD4$^+$ T cells: WT vs WT+DSS, P = 0.0453; B cells, CD8$^+$ T cells, NK cells and NKT cells: no statistical difference between *Mdr2*$^{-/-}$ vs *Mdr2*$^{-/-}$+DSS). **e** Western blotting images of liver NLRP3 (WT, n = 5; WT+DSS, *Mdr2*$^{-/-}$, *Mdr2*$^{-/-}$+DSS, n = 6). **f-h** Analysis of gene expression in the liver of WT and *Mdr2*$^{-/-}$ mice with acute colitis (WT, n = 5; *Mdr2*$^{-/-}$,n = 10; WT+DSS, n = 7; *Mdr2*$^{-/-}$+DSS, n = 12); one-way ANOVA with Bonferroni's multiple comparison test (*Rela*: WT vs *Mdr2*$^{-/-}$, 95% CI -0.8429 to -0.06854, P = 0.0168; WT vs WT+DSS , 95% CI -1.014 to -0.1862, P = 0.0028; *Mdr2*$^{-/-}$ vs *Mdr2*$^{-/-}$+DSS, 95% CI -0.6093 to -0.004023, P = 0.0462; *Nfkb1*: WT vs *Mdr2*$^{-/-}$, 95% CI -1.080 to -0.1265, P = 0.0112; *Tlr4*: WT vs *Mdr2*$^{-/-}$, 95% CI -1.815 to -0.1796, P = 0.0146; WT vs WT+DSS , 95% CI -1.778 to -0.03058, P = 0.0414; *Ccl5*: WT vs WT+DSS, 95% CI -3.930 to -0.6451, P = 0.0054; *Mdr2*$^{-/-}$ vs *Mdr2*$^{-/-}$+DSS, 95% CI -2.632 to -0.02579, P = 0.0450; *Ifng*: *Mdr2*$^{-/-}$ vs *Mdr2*$^{-/-}$+DSS, 95% CI -3.425 to 0.2328, P = 0.0844; *Il10*: WT vs WT+DSS, 95% CI -19.71 to -8.097, P

< 0.0001; *Icam-1*: WT vs WT+DSS, 95% CI -10.84 to -1.090, P = 0.0144; *Mdr2*$^{-/-}$ vs *Mdr2*$^{-/-}$+DSS, 95% CI -7.697 to -0.8029, P = 0.0136; *F4/80*: WT vs WT+DSS, 95% CI -4.813 to -0.2342, P = 0.0286; *Mdr2*$^{-/-}$ vs *Mdr2*$^{-/-}$+DSS, 95% CI -3.379 to -0.03107, P = 0.0453); Kruskal-Wallis test with Dunn's multiple comparison test (*Relb*: *Mdr2*$^{-/-}$ vs *Mdr2*$^{-/-}$+DSS, P = 0.0402; *Rel*: *Mdr2*$^{-/-}$ vs *Mdr2*$^{-/-}$+DSS, P = 0.0186; *Nfkb2*: *Mdr2*$^{-/-}$ vs *Mdr2*$^{-/-}$+DSS, P = 0.0402; *Nlrp3*: *Mdr2*$^{-/-}$ vs *Mdr2*$^{-/-}$+DSS, P = 0.0082; *Ccl2*: WT vs *Mdr2*$^{-/-}$, P = 0.0224; *Il12a*: WT vs *Mdr2*$^{-/-}$, P = 0.0361; *Il12b*: WT vs *Mdr2*$^{-/-}$, P = 0.0138; WT vs WT+DSS, P = 0.0369; *Il6*: *Mdr2*$^{-/-}$ vs *Mdr2*$^{-/-}$+DSS, no significant difference; *Vcam-1*: WT vs *Mdr2*$^{-/-}$, P = 0.0103; *Nos2*: *Mdr2*$^{-/-}$ vs *Mdr2*$^{-/-}$+DSS, P = 0.0523; *Cox-2*: WT vs *Mdr2*$^{-/-}$, P = 0.0015). All data are displayed as mean ± SEM and considered statistically significant at p < 0.05 (*), p < 0.01 (**), p < 0.001 (***) and p < 0.0001 (****). Source data are provided as a Source Data file.

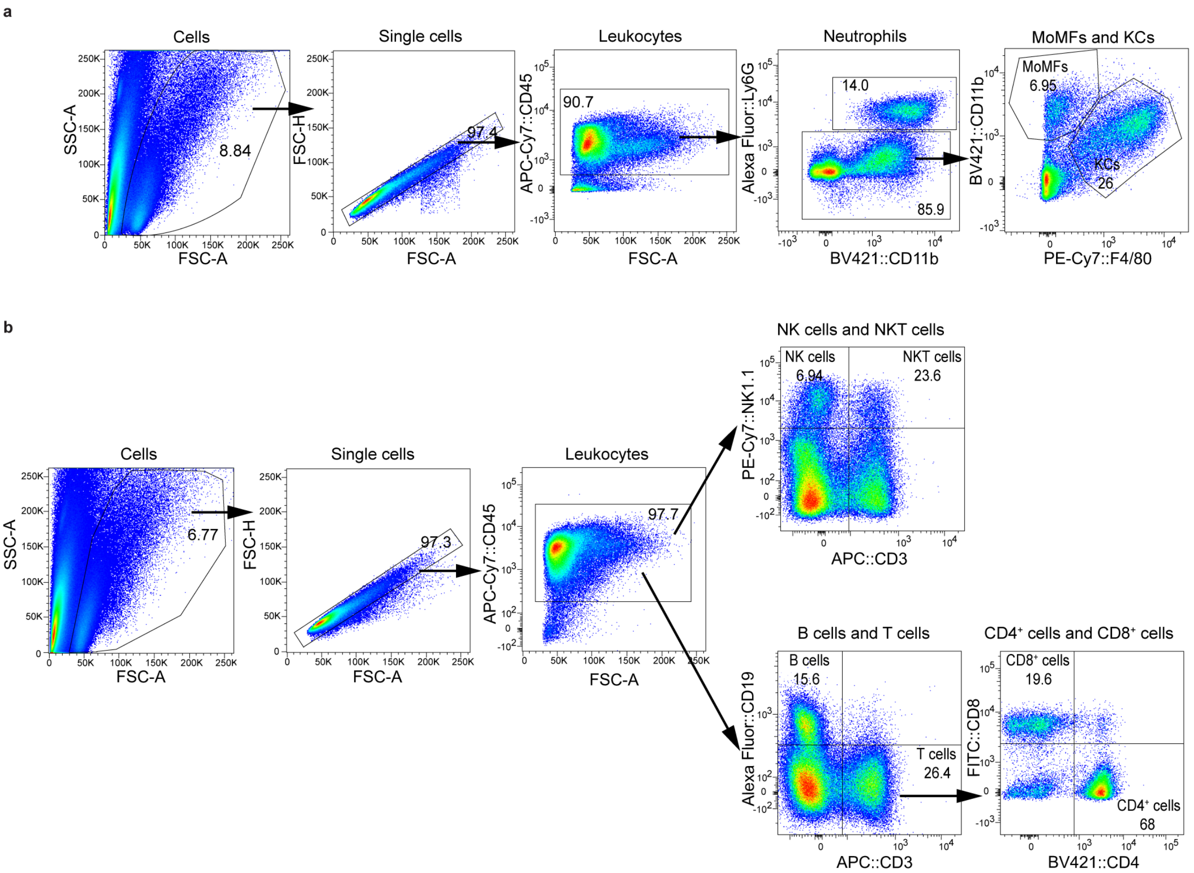

Supplementary Fig. 4 Gating strategies for fluorescence-activated cell sorting. a Gating strategies for myeloid panel. **b** Gating strategies for lymphoid panel.

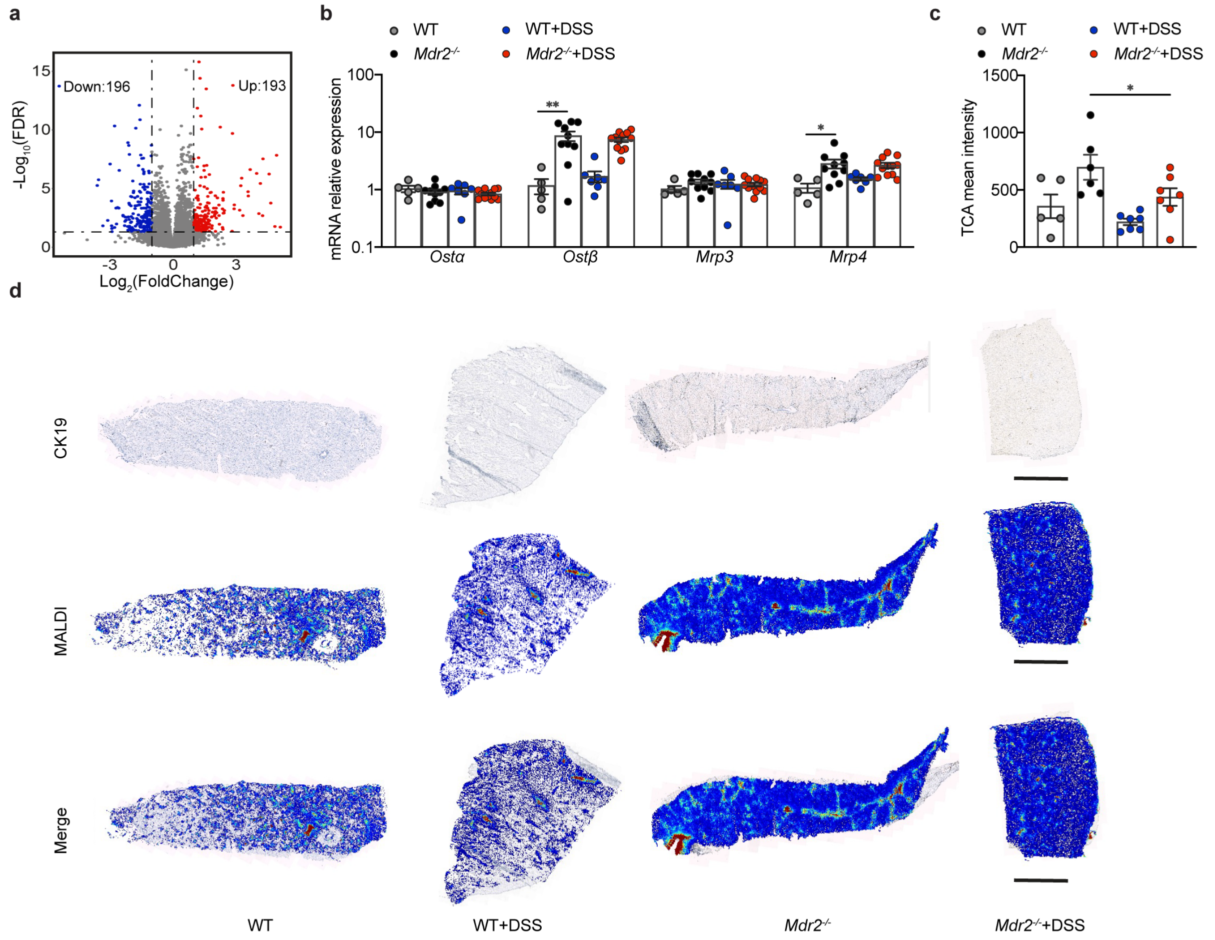

Supplementary Fig. 5 DSS induced colitis modulates hepatic bile acid homeostasis in *Mdr2*$^{-/-}$ mice. a Volcano plot of liver RNA-seq analysis (*Mdr2*$^{-/-}$,n = 10; *Mdr2*$^{-/-}$+DSS, n = 10) Wald test with Benjamini-Hochberg adjustment (two-sided) (padj < 0.05 & Log2foldchange > 1 or < -1). **b** mRNA expression of genes relavant to bile acid transport (WT, n = 5; *Mdr2*$^{-/-}$,n = 10; WT+DSS, n = 7; *Mdr2*$^{-/-}$+DSS, n = 12); one-way ANOVA with Bonferroni's multiple comparison test (*Ostα*: no statistical difference between *Mdr2*$^{-/-}$ vs *Mdr2*$^{-/-}$+DSS; *Mrp4*: WT vs *Mdr2*$^{-/-}$, 95% CI -3.304 to -0.2167, P = 0.02); Kruskal-Wallis test with Dunn's multiple comparison test (*Ostβ*: WT vs *Mdr2*$^{-/-}$, P = 0.004; *Mrp3*: no statistical difference between *Mdr2*$^{-/-}$ vs *Mdr2*$^{-/-}$+DSS). **c** Quantification of TCA intensity in MALDI-MSI (WT, n = 5; *Mdr2*$^{-/-}$,n = 6; WT+DSS, n = 7; *Mdr2*$^{-/-}$+DSS, n = 7); one-way ANOVA with Bonferroni's multiple comparison test (*Mdr2*$^{-/-}$ vs *Mdr2*$^{-/-}$+DSS, 95% CI 26.43 to 493.3, P = 0.0308). **d** Whole slide scans of CK19 staining, MALDI-MSI of taurocholic acid and merged images from liver sections (scale bar, 2 mm). All data are graphed as mean ± SEM and considered significant at p < 0.05 (*), p < 0.01 (**), p < 0.001 (***) and p < 0.0001 (****). Source data are provided as a Source Data file.

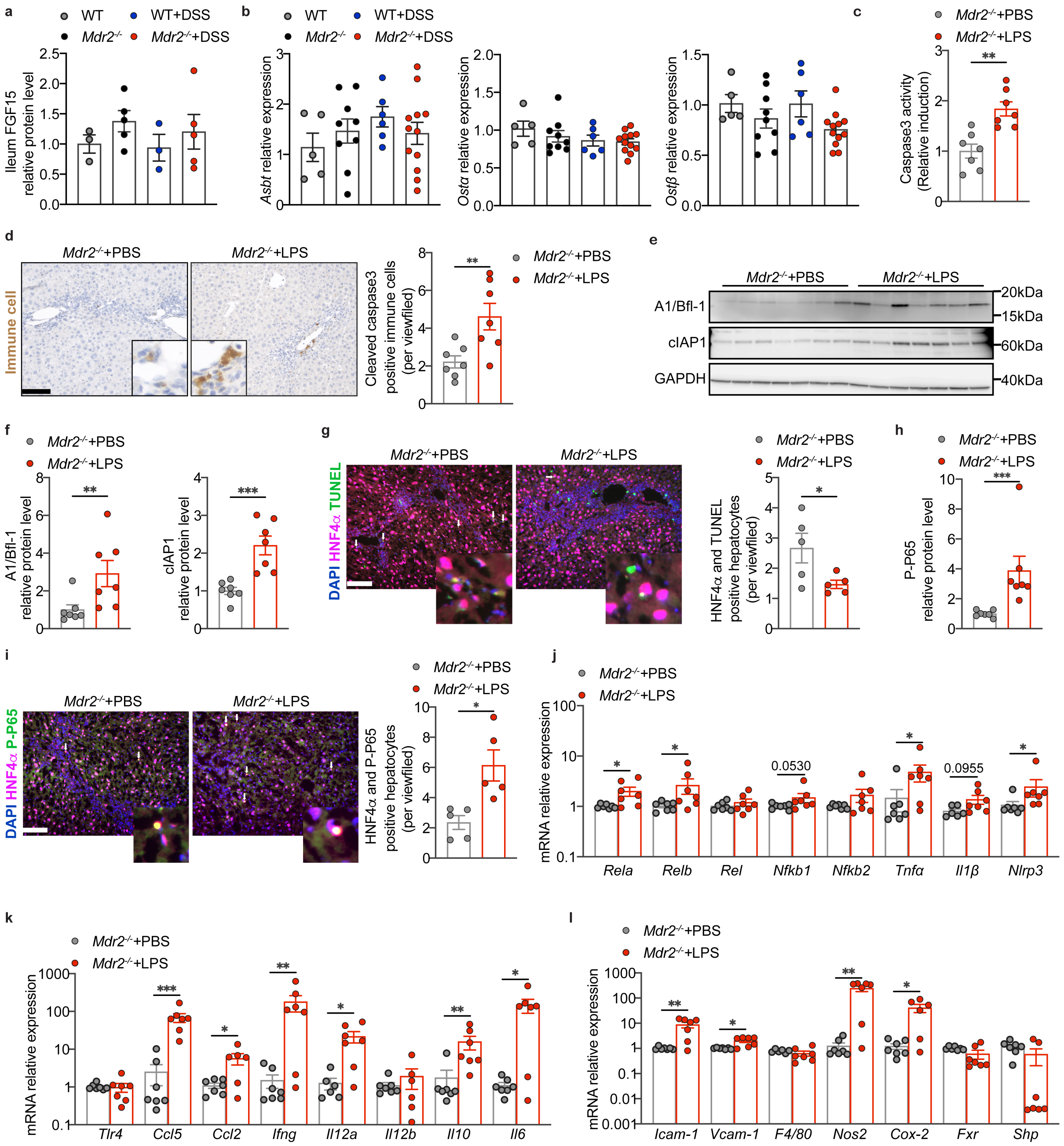

Supplementary Fig. 6 Ileum FXR signaling is not activated in acute DSS treated *Mdr2*$^{-/-}$ mice and LPS injection increases liver inflammation while reducing hepatocyte apoptosis in *Mdr2*$^{-/-}$ mice. **a** Quantification of ileum FGF15 protein (WT, n = 3; WT+DSS, n = 3; *Mdr2*$^{-/-}$,n = 5; *Mdr2*$^{-/-}$+DSS, n = 5); one-way ANOVA with Bonferroni's multiple comparison test (no significant difference between groups). **b** Ileum mRNA expression of *Fxr* regulated genes (WT, n = 5; WT+DSS, n = 6; *Mdr2*$^{-/-}$,n = 9; *Mdr2*$^{-/-}$+DSS, n = 12); one-way ANOVA with Bonferroni's multiple comparison test (*Asbt*, *Ostα* and *Ostβ:* no significant difference between groups). **c** Analysis of liver caspase 3 activity (n = 7 mice per group); unpaired two-tailed Student's t-test (*Mdr2*$^{-/-}$+PBS vs *Mdr2*$^{-/-}$+LPS, P = 0.0011). **d** Cleaved caspase 3 staining in the liver of LPS-injected *Mdr2*$^{-/-}$ mice (n = 7 mice per group); unpaired two-tailed Student's t-test (*Mdr2*$^{-/-}$+PBS vs *Mdr2*$^{-/-}$+LPS, P = 0.0092) (scale bar, 100 μm). **e-f** Western blot of liver A1/Bfl-1 and cIAP1 (n = 7 mice per group); unpaired two-tailed Student's t-test (cIAP1: *Mdr2*$^{-/-}$+PBS vs *Mdr2*$^{-/-}$+LPS, P = 0.0007); Two-tailed Mann–Whitney test (A1/Bfl-1: *Mdr2*$^{-/-}$+PBS vs *Mdr2*$^{-/-}$+LPS, P = 0.007. **g** HNF4α and TUNEL co-staining in the liver of LPS or PBS treated *Mdr2*$^{-/-}$ mice (scale bar, 100 μm) (n = 5 mice per group); unpaired two-tailed Student's t-test (*Mdr2*$^{-/-}$+PBS vs *Mdr2*$^{-/-}$+LPS, P = 0.0459). **h** Analysis of liver phosphorylated NF-κB P65 western blot (n = 7 mice per group). Two-tailed Mann–

Whitney test (*Mdr2*$^{-/-}$+PBS vs *Mdr2*$^{-/-}$+LPS: P-P65, P = 0.0006). **i** HNF4α and Phosphorylated NF-κB P65 co-staining in the liver of LPS injected *Mdr2*$^{-/-}$ mice (n = 5 mice per group) (scale bar, 100 μm): unpaired two-tailed Student's t-test (*Mdr2*$^{-/-}$+PBS vs *Mdr2*$^{-/-}$+LPS, P = 0.0101). **j-l** mRNA expression of genes involved in inflammation in the liver of LPS injected *Mdr2*$^{-/-}$ mice (n = 7 mice per group); unpaired two-tailed Student's t-test (*Mdr2*$^{-/-}$+PBS vs *Mdr2*$^{-/-}$+LPS: *Rela*, P = 0.0315; *Il1*β, P = 0.0955; *Ccl2*, P = 0.0231; *Il12a*, P =0.0248; *Icam-1*, P = 0.0048; *Cox-2*, P = 0.0129; *Rel*, *Nfkb2* and *F4/80,* no significant difference); Two-tailed Mann–Whitney test (*Mdr2*$^{-/-}$+PBS vs *Mdr2*$^{-/-}$+LPS: *Relb*, P = 0.0262; *Nfkb1*, P = 0.053; *Tnfα*, P = 0.0379; *Nlrp3*, P = 0.0175; *Ccl5*, P = 0.0006; *Ifng*, P = 0.0070; *Il10*, P = 0.0064; *Il6*, P = 0.0344; *Vcam-1*, P = 0.0111; *Nos2*, P = 0.0023; *Tlr4* and *Il12b*, no significant difference). All data are mean ± SEM and considered significant at p < 0.05 (*), p < 0.01 (**), p < 0.001 (***) and p < 0.0001 (****). Source data are provided as a Source Data file.

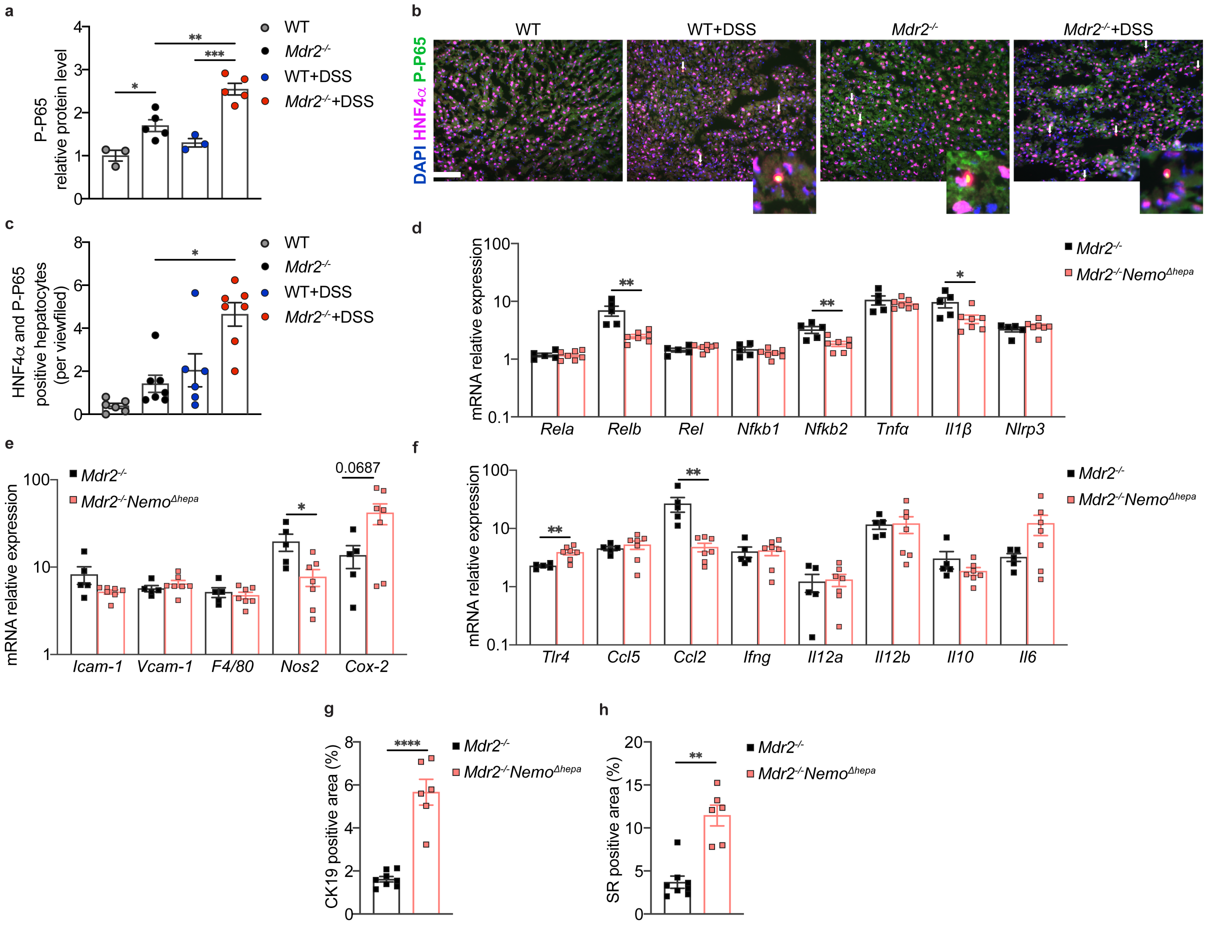

Supplementary Fig. 7 NF-κB is activated in the liver of *Mdr2*$^{-/-}$ mice with acute colitis and hepatocytic NEMO deletion shows minor effect on the expression of inflammatory genes. a Analysis of phosphorylated NF-κB P65 western blot in the liver of acute DSS treated *Mdr2*$^{-/-}$ mice (WT, n = 3; *Mdr2*$^{-/-}$,n = 5; WT+DSS, n = 3; *Mdr2*$^{-/-}$+DSS, n = 5). one-way ANOVA with Bonferroni's multiple comparison test (P-P65: WT vs *Mdr2*$^{-/-}$, 95% CI -1.283 to -0.1139, P = 0.0173; *Mdr2*$^{-/-}$ vs *Mdr2*$^{-/-}$+DSS, 95% CI -1.354 to -0.3411, P = 0.0014; WT+DSS vs *Mdr2*$^{-/-}$+DSS, 95% CI -1.830 to -0.6607, P = 0.0002); **b-c** HNF4α and phosphorylated NF-κB P65 co-staining in the liver of acute DSS treated *Mdr2*$^{-/-}$ mice (WT, n = 6; *Mdr2*$^{-/-}$,n = 7; WT+DSS, n = 6; *Mdr2*$^{-/-}$+DSS, n = 7) (scale bar, 100 μm). Kruskal-Wallis test with Dunn's multiple comparison test (*Mdr2*$^{-/-}$ vs *Mdr2*$^{-/-}$+DSS, P = 0.0219). **d-f** Analysis of gene expression in the liver of *Mdr2*$^{-/-}$ and *Mdr2*$^{-/-}$*Nemo*$^{\Delta hepa}$ mice (*Mdr2*$^{-/-}$,n = 5; *Mdr2*$^{-/-}$*Nemo*$^{\Delta hepa}$, n = 7); unpaired two-tailed Student's t-test (*Mdr2*$^{-/-}$ vs *Mdr2*$^{-/-}$*Nemo*$^{\Delta hepa}$: *Relb*, P = 0.0031; *Nfkb2*, P = 0.0080; *Il1β*, P = 0.0314; *Tlr4*, P = 0.0050; *Ccl2*, P = 0.0061; *Nos2*, P = 0.0167; *Cox-2*, P = 0.0687; *Rela*, *Rel*, *Nfkb1, Tnfα, Nlrp3, Ccl5, Ifng, Il12a, Il12b, Il6, Icam-1, Vcam-1* & *F4/80,* no significant difference); Two-tailed Mann–Whitney test (*Mdr2*$^{-/-}$ vs *Mdr2*$^{-/-}$*Nemo*$^{\Delta hepa}$: *Il10*, no significant difference). **g-h** Quantification of CK19 and Sirius red stainings from liver sections of *Mdr2*$^{-/-}$ and *Mdr2*$^{-/-}$*Nemo*$^{\Delta hepa}$ mice (*Mdr2*$^{-/-}$,n = 8; *Mdr2*$^{-/-}$*Nemo*$^{\Delta hepa}$, n = 6); unpaired two-tailed Student's t-test (*Mdr2*$^{-/-}$ vs *Mdr2*$^{-/-}$*Nemo*$^{\Delta hepa}$: CK19 positive area, P < 0.0001); Two-tailed Mann–Whitney test (*Mdr2*$^{-/-}$ vs *Mdr2*$^{-/-}$*Nemo*$^{\Delta hepa}$: SR positive area, P = 0.0027). All data are mean ± SEM and considered significant at p < 0.05 (*), p < 0.01 (**), p < 0.001 (***) and p < 0.0001 (****). Source data are provided as a Source Data file.

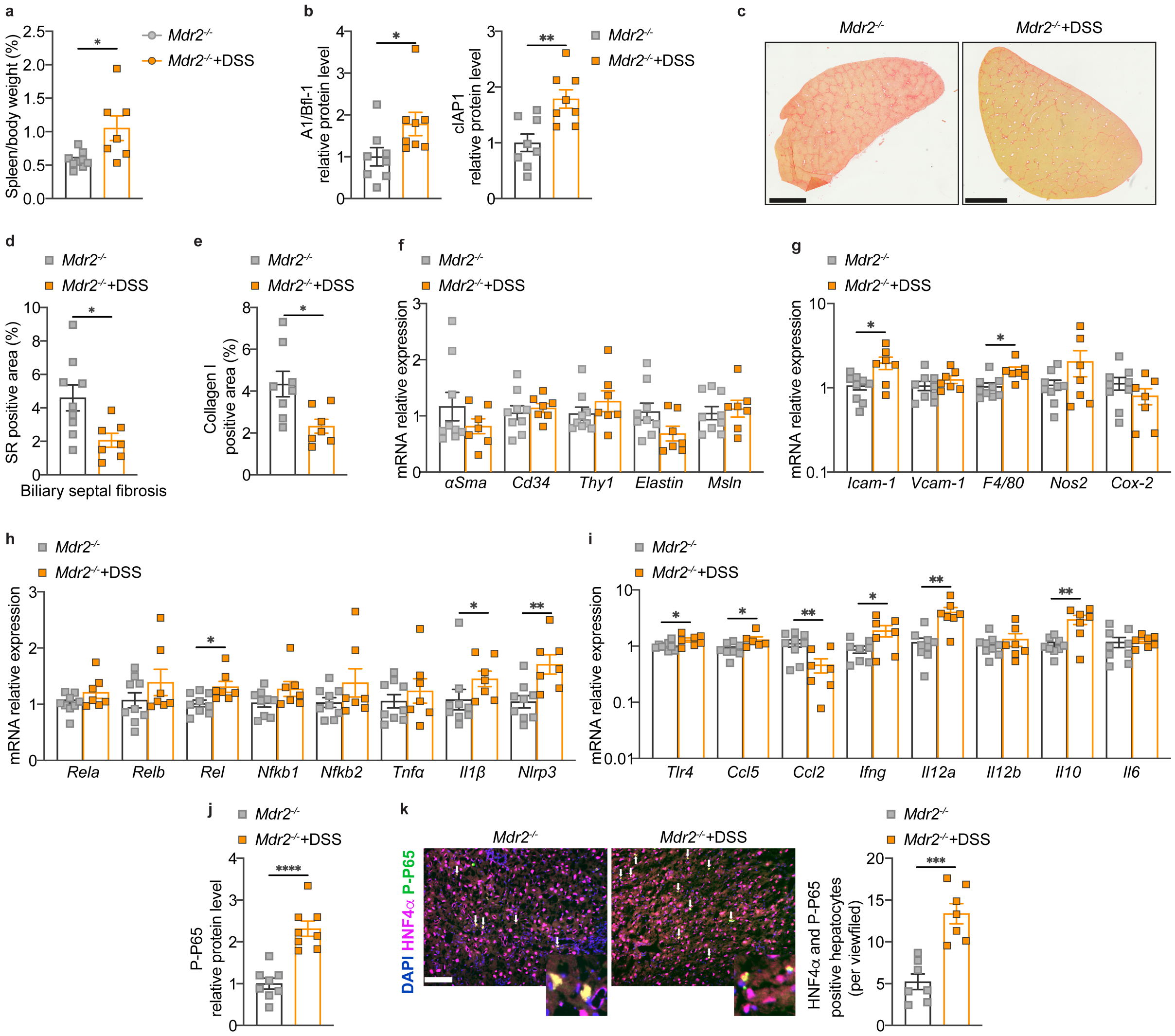

Supplementary Fig. 8 DSS-induced chronic colitis mitigates biliary septal fibrosis while aggrevating liver inflammation in $Mdr2^{-/-}$ mice. a Spleen to body weight ratio ($Mdr2^{-/-}$, n = 9; $Mdr2^{-/-}$ +DSS, n = 7); unpaired two-tailed Student's t-test ($Mdr2^{-/-}$ vs $Mdr2^{-/-}$+DSS, P = 0.0129). **b** Analysis of liver A1/Bfl-1 and cIAP1 western blot (n = 8 mice per group); unpaired two-tailed Student's t-test (cIAP1: $Mdr2^{-/-}$ vs $Mdr2^{-/-}$+DSS, P = 0.0038); Two-tailed Mann–Whitney test (A1/Bfl-1: $Mdr2^{-/-}$ vs $Mdr2^{-/-}$+DSS, P = 0.0281). **c** Representative images of whole slide scan from liver Sirius red staining (n = 7 per group) (scale bar, 2 mm). **d-e** Quantification of biliary septal fibrosis ($Mdr2^{-/-}$,n = 9; $Mdr2^{-/-}$+DSS, n = 7) and collagen I positive area ($Mdr2^{-/-}$,n = 8; $Mdr2^{-/-}$+DSS, n = 7); unpaired two-tailed Student's t-test ($Mdr2^{-/-}$ vs $Mdr2^{-/-}$+DSS: biliary septal fibrosis, P = 0.019; collagen I positive area, P = 0.0153). **f** Liver real-time PCR analysis of HSCs markers (*αSma*) and PFs markers (*Cd34, Thy1, Elastin, Msln*) ($Mdr2^{-/-}$,n = 9; $Mdr2^{-/-}$+DSS, n = 7); unpaired two-tailed Student's t-test (no statistical difference between groups). **g-i** qPCR analysis of liver inflammatory genes ($Mdr2^{-/-}$,n = 9; $Mdr2^{-/-}$+DSS, n = 7); unpaired two-tailed Student's t-test ($Mdr2^{-/-}$ vs $Mdr2^{-/-}$+DSS: *Nlrp3*, P = 0.0044; *Tlr4*, P = 0.0288; *Ccl5*, P = 0.0328; *Ccl2*, P = 0.0098; *Ifng*, P = 0.0311; *Il12a*, P = 0.0016; *Il10*, P = 0.0017; *Icam-1*, P = 0.0112; *Rela*, *Nfkb1, Tnfα, Il12b, Il6, Vcam-1, Nos2* & *Cox-2,* no statistical differences); Two-tailed Mann–Whitney test ($Mdr2^{-/-}$ vs $Mdr2^{-/-}$+DSS: *Rel,* P = 0.0229; *Il1β*, P = 0.0229; *Il18*, P = 0.0311; *F4/80,* P = 0.0115; *Relb* & *Nfkb2,* no statistical differences). **j** Analysis of phosphorylated NF-κB P65 western blot in the liver of chronic DSS treated $Mdr2^{-/-}$ mice (n = 8 mice per group). unpaired two-tailed Student's t-test (P-P65: $Mdr2^{-/-}$ vs $Mdr2^{-/-}$+DSS, P < 0.0001). **k** Liver HNF4α and phosphorylated NF-κB P65 co-staining (n = 7 mice per group) (scale bar, 100 μm): unpaired two-tailed Student's t-test ($Mdr2^{-/-}$ vs $Mdr2^{-/-}$+DSS, P = 0.0002). All data

are mean ± SEM and considered significant at $p < 0.05$ (*), $p < 0.01$ (**), $p < 0.001$ (***) and $p < 0.0001$ (****). Source data are provided as a Source Data file.

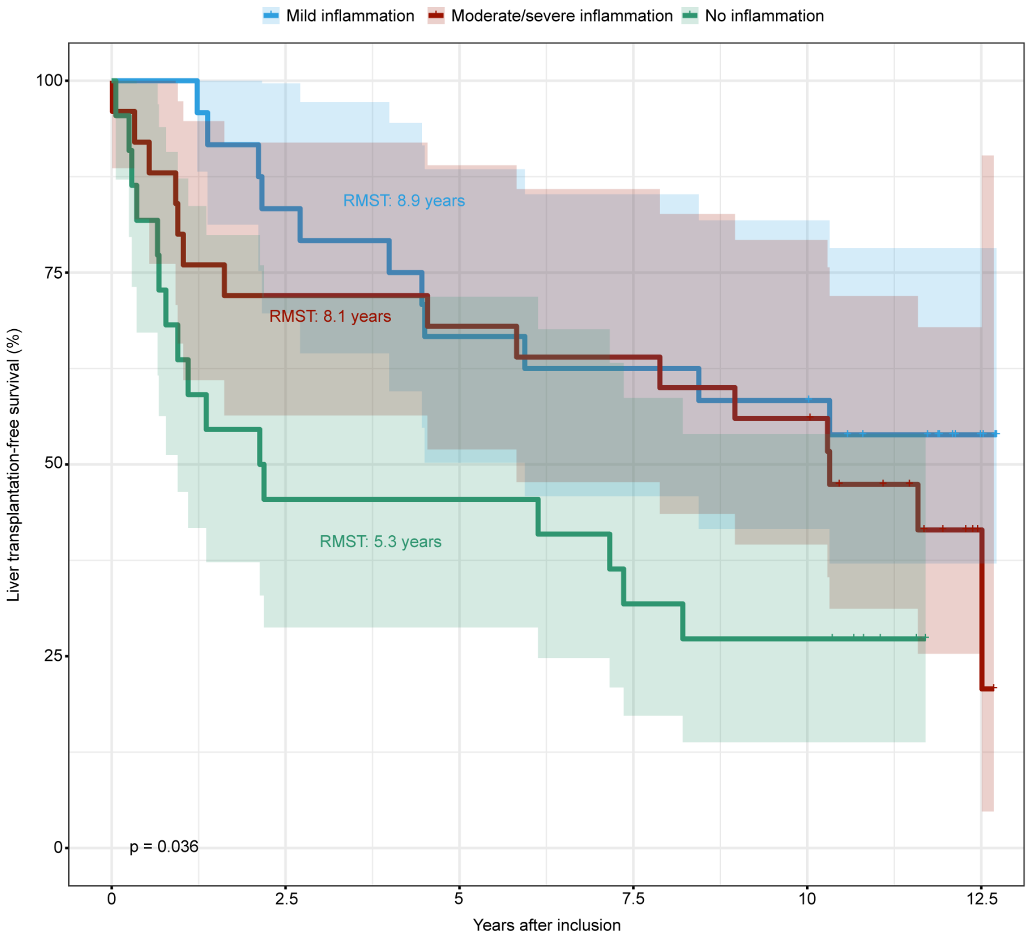

Supplementary Fig. 9 The severity of intestine inflammation is associated with different survival estimates. Kaplan-Meier analysis for non-transplanted PSC patients (N = 70, after exclusion of patients who experienced outcome <3 months after inclusion). Survival probabilities for each group are shown using the Kaplan-Meier estimator with corresponding (coloured) 95 % confidence intervals and the log-rank test (P = 0.036). Average survival times are shown in figure using restricted mean survival time (RMST). Source data are provided as a Source Data file.

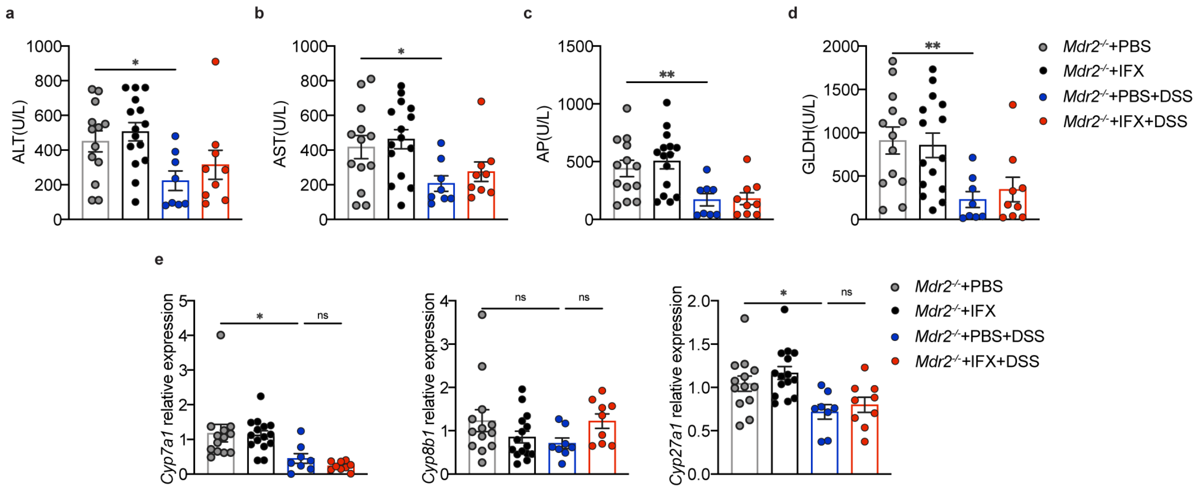

Supplementary Fig. 10 Infliximab (IFX) does not aggravate liver injury and fails to restore bile acid synthesis in *Mdr2*$^{-/-}$ mice after DSS treatment. a-d Serum markers of liver injury (*Mdr2*$^{-/-}$+PBS, n = 13; *Mdr2*$^{-/-}$+IFX, n = 15; *Mdr2*$^{-/-}$+PBS+DSS, n = 8; *Mdr2*$^{-/-}$+IFX+DSS, n = 9). Kruskal-Wallis test with Dunn's multiple comparison test (ALT: *Mdr2*$^{-/-}$+PBS vs *Mdr2*$^{-/-}$+PBS+DSS, P = 0.0151; AST: *Mdr2*$^{-/-}$+PBS vs *Mdr2*$^{-/-}$+PBS+DSS, P = 0.0318; AP: *Mdr2*$^{-/-}$+PBS vs *Mdr2*$^{-/-}$+PBS+DSS, P = 0.0099; GLDH: *Mdr2*$^{-/-}$+PBS vs *Mdr2*$^{-/-}$+PBS+DSS, P = 0.0052). **e** mRNA expression of liver *Cyp7a1*, *Cyp8b1*, and *Cyp27a1* (*Mdr2*$^{-/-}$+PBS, n = 13; *Mdr2*$^{-/-}$+IFX, n = 15; *Mdr2*$^{-/-}$+PBS+DSS, n = 8; *Mdr2*$^{-/-}$+IFX+DSS, n = 9). Kruskal-Wallis test with Dunn's multiple comparison test (*Cyp7a1*: *Mdr2*$^{-/-}$+PBS vs *Mdr2*$^{-/-}$+PBS+DSS, P = 0.0205; *Mdr2*$^{-/-}$+PBS+DSS vs *Mdr2*$^{-/-}$+IFX+DSS, no significant difference; *Cyp8b1*: *Mdr2*$^{-/-}$+PBS vs *Mdr2*$^{-/-}$+PBS+DSS and *Mdr2*$^{-/-}$+PBS+DSS vs *Mdr2*$^{-/-}$+IFX+DSS, no significant difference); one-way ANOVA with Bonferroni's multiple comparison test (*Cyp27a1*: *Mdr2*$^{-/-}$+PBS vs *Mdr2*$^{-/-}$+PBS+DSS, 95% CI 0.02962 to 0.6220, P = 0.0285; *Mdr2*$^{-/-}$+PBS+DSS vs *Mdr2*$^{-/-}$+IFX+DSS, no significant difference). All data are mean ± SEM and considered significant at p < 0.05 (*), p < 0.01 (**), p < 0.001 (***) and p < 0.0001 (****). All data are graphed as mean ± SEM and considered significant at p < 0.05 (*), p < 0.01 (**), p < 0.001 (***) and p < 0.0001 (****). Source data are provided as a Source Data file.

183 **Supplementary Fig. 11 Graphical abstract.**

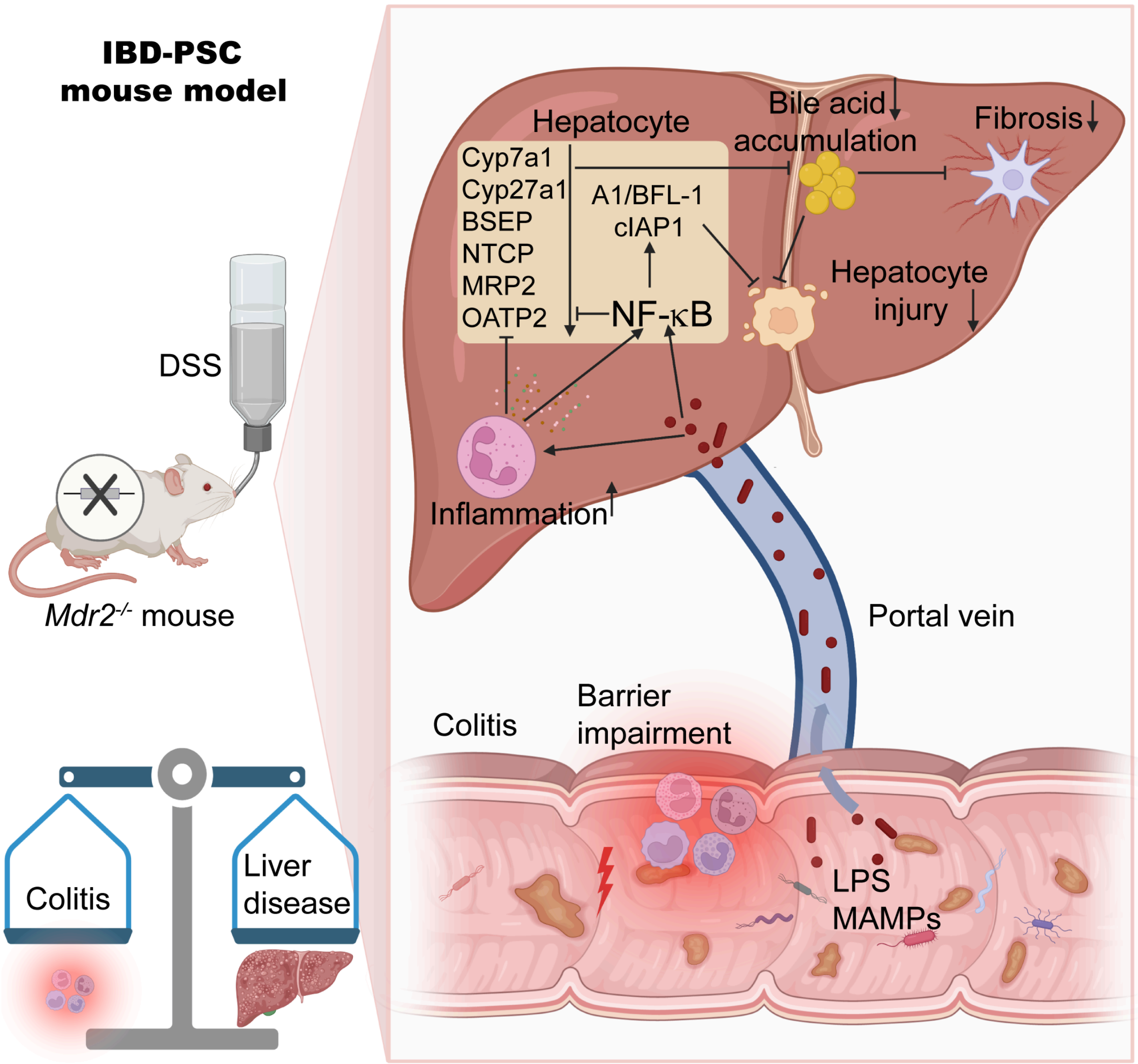

**Variable**	**Histological inflammation, N = 53**	**No histological inflammation, N = 22**	**P-value**
Age at inclusion	37 (13)	46 (11)	0.0079
Age at PSC diagnosis	33 (13)	36 (13)	0.17
Female	9 / 53 (17%)	5 / 22 (23%)	0.54
Small-duct PSC	1 / 53 (1.9%)	1 / 22 (4.5%)	0.50
Inflammatory bowel disease			0.00019
Ulcerative colitis	44 / 53 (83%)	10 / 22 (45%)	
Crohn's disease	5 / 53 (9.4%)	1 / 22 (4.5%)	
Indeterminate colitis	1 / 53 (1.9%)	1 / 22 (4.5%)	
None	3 / 53 (5.7%)	10 / 22 (45%)	
**Disease scores**			
AOM score	1.81 (0.61)	2.16 (0.80)	0.057
Mayo PSC score	0.07 (1.01)	0.72 (1.27)	0.041
APRI score	0.85 (0.80)	1.23 (1.28)	0.25
FIB-4 score	1.23 (1.24)	2.17 (1.72)	0.0036
**Medication**			
Ursodeoxycholic acid	24 / 53 (45%)	12 / 22 (55%)	0.46
5-aminosalicylate	32 / 53 (60%)	8 / 22 (36%)	0.058
Prednisolone	35 / 53 (66%)	8 / 22 (36%)	0.018
Budesonide	1 / 53 (1.9%)	0 / 22 (0%)	1
Azathioprine	11 / 53 (21%)	2 / 22 (9.1%)	0.32
**Serum parameters**			
Hemoglobin, g/dL	14.05 (1.68)	13.75 (1.42)	0.32
Platelet count, 10^9/L	297 (123)	248 (105)	0.082
Creatinine, µmol/L	68 (13)	74 (37)	0.89
Bilirubin, mg/dL	1.96 (2.77)	2.72 (2.71)	0.022
Albumin, g/L	41.1 (4.2)	40.1 (6.2)	0.54
INR	1.06 (0.13)	1.10 (0.12)	0.15
AST, U/L	80 (59)	89 (62)	0.63
ALT, U/L	115 (101)	113 (109)	0.73
ALP, U/L	274 (204)	284 (221)	0.74
Fecal calprotectin, mg/kg	265 (495)	131 (311)	0.47
Leukocytes, 10^9/L	7.27 (3.35)	6.15 (2.07)	0.21
CRP, mg/L	7 (8)	7 (7)	0.58

Supplementary Table 1. Baseline characteristics of the Oslo PSC cohort with or without intestine histological inflammation. The data are presented as Mean (SD) or n/N (%). Statistical comparisons were calculated with Wilcoxon rank sum test, Fisher's exact test or Pearson's Chi-squared (two-sided).

Supplementary Table 2. Primers used for qPCR.

Gene	Forward primer(5'-3')	Reverse primer(5'-3')
Nlrp3	TGTGAGAAGCAGGTTCTACTCT	TGTAGCGACTGTTGAGGTCCA
Nfkb2	GAGAAGCCTGGTGGACACATA	CCACCAGCCAGCTTGTAACTA
Asbt	GTCTGTCCCCCAAATGCAACT	CACCCCATAGAAAACATCACCA
Tnfα	GACCCTCACACTCAGATCATCTTCT	CCACTTGGTGGTTTGCTACGA
Cyp7a1	AGCAACTAAACAACCTGCCAGTACTA	GTCCGGATATTCAAGGATGCA
Bsep	CTGCCAAGGATGCTAATGCA	CGATGGCTACCCTTTGCTTCT
Shp	CGATCCTCTTCAACCCAGATG	AGGGCTCCAAGACTTCACACA
Nfkb1	GGTGCAGTGTCTTGAGCTTTC	GGAGGGACAGCAGTAACAACA
Ntcp	CACCATGGAGTTCAGCAAGA	AGCACTGAGGGGCATGATAC
Fxr	TCCAGGGTTTCAGACACTGG	GCCGAACGAAGAAACATGG
Col1a1	TGTGTGCGATGACGTGCAAT	GGGTCCCTCGACTCCTACA
Tlr4	TTCAGAACTTCAGTGGCTGGATT	CCATGCCTTGTCTTCAATTGTTT
Il1β	GACCTGTTCTTTGAAGTTGACG	AGATTTGAAGCTGGATGCTCTC
Ostα	GTCTCAAGTGATGAACTGCCA	TTGAGTGCTGAGTCCAGGTC
Ostβ	GACAAGCATGTTCCTCCTGAG	GATGCAGGTCTTCTGGTGTTTC
Timp1	GTGCACAGTGTTTCCCTGTTT	AGGACCTGATCCGTCCACAA
Mmp2	AACGGTCGGGAATACAGCAG	GTAAACAAGGCTTCATGGGGG
αSma	TGACAGAGGCACCACTGAACC	TCCAGAGTCCAGCACAATACCAGT
Ccl5	GCTGCTTTGCCTACCTCTCC	TCGAGTGACAAACACGACTGC
Beta-actin	AGCTGCGTTTTACACCCTTTCT	AGCCATGCCAATGTTGTCTCT
Gapdh	ACAACTTTGGTATCGTGGAAGG	GCCATCACGCCACAGTTTC
Rel	GACAACCCAAGACTTGTGAGC	AGACTGGCAGGAGACATCTGA
Rela	TGTGTCCATGTCTCACTCCAC	CTTCAGGGTACTCCATCAGCA
Occludin	GCTGTGATGTGTGTTGAGCT	GACGGTCTACCTGGAGGAAC
Relb	TTGGTACTGCTAGCCTTGTGG	TGCCTCCTCTGTTACCTCAGA
Cyp27a1	CCAGGCACAGGAGAGTACG	GGGCAAGTGCAGCACATAG
Cyp8b1	CCTCTGGACAAGGGTTTTGTG	GCACCGTGAAGACATCCCC
Mrp3	CACCATCAGCTCGGCTACAT	CAGGTCCACCCATGAGACAC
Col1a2	GCTGGTGTAATGGGTCCTCC	CGACCGGCATCTCCATTAGG
Col3a1	TCCTGGTGGTCCTGGTACTG	AGGAGAACCACTGTTGCCTG
Mrp4	ACCTCGGAGAGGAGCTTCAACGG	TGTTCACATCGTTGGACAGCAGGT
Cd34	TCCTGATGAACCGTCGCAGTTG	TGTCAGCCACCACATGTTGTC
Desmin	AACTTCCGAGAAACCAGCCC	CTGTGTAGCCTCGCTGACAA
Thy1	GCTCTCAGTCTTGCAGGTGTC	CAGGCGAAGGTTTTGGTTCA
Elastin	CAGAGCTCCTCCTCCTCCTC	CTTGCTCAACCTCCTCCATC
Msln	CCCATCGAAGTGGTCAGTCTC	GGTGTATGACGGTCAGCTTAGA
Ccl2	GTGTTGGCTCAGCCAGATGC	GACACCTGCTGCTGGTGATCC
Ifng	CTTCAGCAACAGCAAGGC	CGAATCAGCAGCGACTCC
Il12a	CCACTGGAACTACACAAGAA	GCACAGGGTCATCATCAA
Il12b	GTACCTACGCAGCCCTGATTG	GTTAAATGCCCGCAGAGCCAG
Il10	GGCGCTGTCATCGATTTCTC	CTCTTCACCTGCTCCACTGC
Il6	ACTTCACAAGTCGGAGGCTT	TGCAAGTGCATCATCGTTGT
Icam-1	GCCAGGAGCCTCCGGACTTTCG	GGCAGGAAACAGGCCTTCCAGGG
Vcam-1	ACTCCCGTCATTGAGGATATTGGA	CGTTGTATTCCTGGGAGAGATGTAG
F4/80	GGAAAGCACCATGTTAGCTGC	CCTCTGGCTGCCAAGTTAATG
Nos2	GTTCACCCAGTTGTGCATCG	GCACATCAAAGCGGCCATAG
Cox-2	GTGGAAAAACCTCGTCCAGA	GCTCGGCTTCCAGTATTGAG

Supplementary Table 3. Antibodies used for western blot, staining and flow cytometry.

Antibodies	Company	Clone number	Cat number
Rabbit monoclonal anti-MUC2, dil: 1:200	Abcam	EPR23479-47	Cat#ab272692
Rabbit polyclonal anti-IL-1 beta, dil: 1:1000	Abcam		Cat#ab9722
Rabbit monoclonal anti-pro Caspase-1 + p10 + p12, dil: 1:800	Abcam	EPR16883	Cat#ab179515
Rabbit monoclonal anti-Cytokeratin 19, dil: 1:200	Abcam	EP1580Y	Cat#ab52625
Rabbit monoclonal anti-alpha smooth muscle Actin, dil: 1:800	Abcam	E184	Cat#ab32575
Mouse monoclonal anti-FGF15, dil: 1:500	Santa Cruz Biotechnology	D-9	Cat#sc-514647
Mouse monoclonal anti-CYP7A1, dil: 1:500	Santa Cruz Biotechnology	E-10	Cat#sc-518007
Rabbit monoclonal anti-Ki67, dil: 1:200	Cell Signaling Technology	D3B5	Cat#12202
Rat monoclonal anti-CD11b, dil: 1:200	BD Biosciences	M1/70	Cat#550282
Rat monoclonal anti-Ly6G, dil: 1:200	BD Biosciences	1A8	Cat#551459
Rat monoclonal anti-CD45, APC-Cy7 conjugated, dil: 1:84	BD Biosciences	30-F11	Cat#557659
Rat monoclonal anti-CD11b, V450 conjugated, dil: 1:84	BD Biosciences	M1/70	Cat#560455
Mouse monoclonal anti-NLRP3, dil: 1:1000	AdipoGen	Cryo-2	Cat#AG-20B-0014
Rabbit monoclonal anti-cIAP1, dil: 1:1000	Cell Signaling Technology	E6R2S	Cat#70008S
Rabbit monoclonal anti-A1/Bfl-1, dil: 1:1000	Cell Signaling Technology	E4P2I	Cat#64310S
Rabbit polyclonal anti-Cleaved Caspase3, dil: 1:200	Cell Signaling Technology	Asp175	Cat#9661S
Rabbit monoclonal anti-Phospho-NF-kB P65, dil: 1:200	Cell Signaling Technology	Ser536	Cat#3033S
Goat polyclonal anti-HNF-4α, dil: 1:200	Santa Cruz Biotechnology	C-19	Cat#SC-6556
Mouse monoclonal anti-P-P65, dil: 1:500	Santa Cruz Biotechnology	27.Ser 536	Cat#SC-136548 HRP
Rabbit polyclonal anti-Occludin, dil: 1:1000	Thermo Fisher Scientific		Cat#71-1500
Rabbit polyclonal anti-ZO-1, dil: 1:200	Thermo Fisher Scientific		Cat#40-2200
Rat monoclonal anti-F4/80, PE-Cy7 conjugated, dil: 1:84	Thermo Fisher Scientific	BM8	Cat#25-4801-82
Rat monoclonal anti-Ly6C, PerCP-Cy5.5 conjugated, dil: 1:84	Thermo Fisher Scientific	HK1.4	Cat#45-5932-82
Rat monoclonal anti-CD19, Alexa Fluor 700 conjugated, dil: 1:84	Thermo Fisher Scientific	eBio1D3 (1D3)	Cat#56-0193-82
Armenian Hamster monoclonal anti-CD3e, APC conjugated, dil: 1:84	Thermo Fisher Scientific	145-2C11	Cat# 17-0031-82
Rat monoclonal anti-CD4, eFluor 450 conjugated, dil: 1:84	Thermo Fisher Scientific	GK1.5	Cat#48-0041-82
Rat monoclonal anti-CD8a, FITC conjugated, dil: 1:84	Thermo Fisher Scientific	53-6.7	Cat# 11-0081-85
Mouse monoclonal anti-NK1.1, PE-Cy7 conjugated, dil: 1:84	Thermo Fisher Scientific	PK136	Cat#25-5941-82
Rabbit polyclonal anti-Collagen I, dil: 1:200	Bio-Rad		Cat#2150-1410

Mouse monoclonal anti-GAPDH, dil: 1:1000	Bio-Rad	6C5	Cat# MCA4739
Mouse monoclonal anti-β-Actin, dil: 1:1000	Sigma-Aldrich	AC-15	Cat#A5441
Rat monoclonal anti-Ly6G, Alexa Fluor 700 conjugated, dil: 1:84	BioLegend	1A8	Cat#127622
Armenian Hamster monoclonal anti-CD11c, APC conjugated, dil: 1:84	BioLegend	N418	Cat#117310
Rat monoclonal anti-I-A/I-E(MHC II), FITC conjugated, dil: 1:84	BioLegend	M5/114.15.2	Cat#107605
Goat polyclonal anti-rabbit IgG, HRP linked, dil: 1:2000	Cell Signaling Technology		Cat#7074
Mouse monoclonal anti-mouse IgGκ light chain immunoglobulins, HRP linked, dil: 1:2000	Santa Cruz Biotechnology		Cat# sc-516102
Goat polyclonal anti-rat IgG(H+L), Cy3 linked, dil: 1:400	Thermo Fisher Scientific		Cat#A10522
Goat polyclonal anti-rabbit IgG(H+L), Alexa Fluo 546 linked, dil: 1:400	Thermo Fisher Scientific		Cat#A-11010
Goat polyclonal anti-rabbit IgG(H+L), Alexa Fluo 488 linked, dil: 1:400	Thermo Fisher Scientific		Cat#A-11008
Donkey polyclonal anti-Goat IgG (H+L) Highly Cross-Adsorbed Secondary Antibody, Alexa Fluor™ Plus 647, dil: 1:400	Thermo Fisher Scientific		Cat#A32849
