# Supplementary material for: Colitis ameliorates cholestatic liver disease via suppression of bile acid synthesis
Source: Nat Commun. 2023 Jun 6;14:3304. doi: 10.1038/s41467-023-38840-8 (PMC10244448; doi:10.1038/s41467-023-38840-8)
Supplement: Supplementary file 2 — Reporting Summary [file 41467_2023_38840_MOESM2_ESM.pdf]

nature portfolio

Corresponding author(s): Christian Trautwein, Kai Markus Schneider

Last updated by author(s): 2023-4-27

Reporting Summary

Nature Portfolio wishes to improve the reproducibility of the work that we publish. This form provides structure for consistency and transparency in reporting. For further information on Nature Portfolio policies, see our Editorial Policies and the Editorial Policy Checklist.

Statistics

For all statistical analyses, confirm that the following items are present in the figure legend, table legend, main text, or Methods section.

n/a	Confirmed	
☐	☒	The exact sample size (*n*) for each experimental group/condition, given as a discrete number and unit of measurement
☐	☒	A statement on whether measurements were taken from distinct samples or whether the same sample was measured repeatedly
☐	☒	The statistical test(s) used AND whether they are one- or two-sided<br>*Only common tests should be described solely by name; describe more complex techniques in the Methods section.*
☐	☒	A description of all covariates tested
☐	☒	A description of any assumptions or corrections, such as tests of normality and adjustment for multiple comparisons
☐	☒	A full description of the statistical parameters including central tendency (e.g. means) or other basic estimates (e.g. regression coefficient) AND variation (e.g. standard deviation) or associated estimates of uncertainty (e.g. confidence intervals)
☐	☒	For null hypothesis testing, the test statistic (e.g. *F*, *t*, *r*) with confidence intervals, effect sizes, degrees of freedom and *P* value noted<br>*Give P values as exact values whenever suitable.*
☒	☐	For Bayesian analysis, information on the choice of priors and Markov chain Monte Carlo settings
☒	☐	For hierarchical and complex designs, identification of the appropriate level for tests and full reporting of outcomes
☐	☒	Estimates of effect sizes (e.g. Cohen's *d*, Pearson's *r*), indicating how they were calculated

Our web collection on statistics for biologists contains articles on many of the points above.

Software and code

Policy information about availability of computer code

Data collection	AxioVision 4.9 (Zeiss), ImageJ 1.52P (NIH, USA),BD FACSDiva 6 (BD Bioscience)
Data analysis	For analyzing sequencing files, FastQC (v0.11.9) was used to evaluate the quality of all data. Next, Salmon (v1.8.0) was applied to perform transcript quantification. Further visualisations were carried out in R (version 4.0.3). Briefly, Salmon quantification results and metadata were imported into R by tximeta package (1.16.1). Differential gene expression was then analyzed using Deseq2 package (1.38.3). When adjusted P < 0.05, expression differences between groups were considered significant. Subsequently, a two-dimensional PCA clustering map was plotted to compare the global gene expression by using ggplot2 package (3.4.1). Pheatmap (1.0.12) served to graph gene expression heatmap. Gene set enrichment analysis (GSEA) was performed using ClusterProfiler (v3.18.1) . Spearman correlation was analyzed with Psych package (2.2.9). FlowJo (version 10.4) was used to analyze Flow cytometry data. Images of western blots and histological stainings were analyzed using ImageJ 1.52P (NIH, USA). R (version 4.0.3) and GraphPad Prism (version 9.0.0) were used to perform statistical analysis and plotting graph. MALDI-MSI data were interpreted using SCiLS Lab MVS (version 2021c).

For manuscripts utilizing custom algorithms or software that are central to the research but not yet described in published literature, software must be made available to editors and reviewers. We strongly encourage code deposition in a community repository (e.g. GitHub). See the Nature Portfolio guidelines for submitting code & software for further information.

Data

Policy information about availability of data

All manuscripts must include a data availability statement. This statement should provide the following information, where applicable:

- Accession codes, unique identifiers, or web links for publicly available datasets
- A description of any restrictions on data availability
- For clinical datasets or third party data, please ensure that the statement adheres to our policy

Data availability
In this study, RNA-seq raw data have been uploaded and deposited in the Sequence Read Archive (SRA) database of National Center for Biotechnology Information (NCBI), which becomes publicly accessible via the accession number PRJNA924130. The Gene Expression Omnibus (GEO) dataset of co-cultured primary human hepatocytes and Kupffer cells treated with LPS is available via the accession number GSE104601. The remaining data are available within the article, supplementary information or source data files. Source data are provided with this paper.

Human research participants

Policy information about studies involving human research participants and Sex and Gender in Research.

Reporting on sex and gender	Of the 75 patients included that had available tissue samples, 14 were female sex as based on biological attribute (extracted from patient records with national identification number indicating biological sex). Inclusion into cohort was based on availability during the prospective inclusion, hence sex distribution was determined by availability, which reflects the epidemiology. Due to sample-size, survival analysis was not performed stratified by sex.
Population characteristics	Patients (N=75) had a confirmed diagnosis of primary sclerosing cholangitis. Mean age of 39.9 years at inclusion, and mean Amsterdam-Oxford model PSC score of 1.92. 62/75 (82,7%) had a confirmed diagnosis of concomitant inflammatory bowel disease.
Recruitment	Patients were prospectively recruited at Rikshospitalet (Oslo, Norway) between 2005-2008, and underwent ileocolonoscopy. Cause of referral was follow-up or confirmation of PSC. Rikshospitalet is a tertiary care hospital, and patients could potentially have more severe liver disease, which may have an impact on generalisability to patients with less severe liver disease.
Ethics oversight	This human study was approved by the Regional Committee for Medical and Health Research Ethics (projects 2015/2140 and 2016/1690) at Rikshospitalet (Oslo, Norway) and all included patients gave informed consent.

Note that full information on the approval of the study protocol must also be provided in the manuscript.

Field-specific reporting

Please select the one below that is the best fit for your research. If you are not sure, read the appropriate sections before making your selection.

[x] Life sciences [ ] Behavioural & social sciences [ ] Ecological, evolutionary & environmental sciences

For a reference copy of the document with all sections, see nature.com/documents/nr-reporting-summary-flat.pdf

Life sciences study design

All studies must disclose on these points even when the disclosure is negative.

Sample size	In human studies, sample size was defined by availability.<br>In mouse studies, sample size was selected based on our group's previous experience (Schneider et al., Nature communications 2022; Schneider et al., Nature metabolism 2021)
Data exclusions	4 patients with histological inflammation of intestine and 1 patient without were excluded to avoid bias caused by patients already being listed for a liver transplantation since they reached endpoint within 3 months from inclusion.
Replication	All of the data were reproduced successfully and across multiple experiments the data were pooled at least from two independent trials.
Randomization	For all experiments involving mice, littermates of male mice were randomly allocated to experimental or control groups.<br>For the human study, inclusion into cohort was based on availability during the prospective inclusion. Therefore, randomization is not relevant.
Blinding	Preparation of libraries for RNA sequencing, analysis of RNA-Seq and flow cytometry data were carried out in a blinded manner.<br>In addition, researchers were blinded to imaging analysis, serum and tissue process as well as to genotype during FITC-dextran assay.

For RNA-sequencing and imaging analysis, the investigators were blinded to group allocation during data collection.
In experiments concerning DSS or LPS treatment, investigators were not blind to the treatment because it must be identified clearly to prevent inter-group cross-contamination.

Reporting for specific materials, systems and methods

We require information from authors about some types of materials, experimental systems and methods used in many studies. Here, indicate whether each material, system or method listed is relevant to your study. If you are not sure if a list item applies to your research, read the appropriate section before selecting a response.

Materials & experimental systems

n/a	Involved in the study
☐	☒ Antibodies
☒	☐ Eukaryotic cell lines
☒	☐ Palaeontology and archaeology
☐	☒ Animals and other organisms
☒	☐ Clinical data
☒	☐ Dual use research of concern

Methods

n/a	Involved in the study
☒	☐ ChIP-seq
☐	☒ Flow cytometry
☒	☐ MRI-based neuroimaging

Antibodies

Antibodies used

IHC and IF stainings:
1,Rabbit monoclonal anti-MUC2, Abcam, clone: EPR23479-47, Cat#ab272692, dil: 1:200
2,Rabbit monoclonal anti-Cytokeratin 19, Abcam, clone: EP1580Y, Cat#ab52625, dil: 1:200
3, Rabbit monoclonal anti-Ki67, Cell Signaling Technology, clone: D3B5, Cat#12202, dil: 1:200
4, Rat monoclonal anti-CD11b, BD Biosciences, clone: M1/70, Cat#550282, dil: 1:200
5, Rat monoclonal anti-Ly6G, BD Biosciences, clone: 1A8, Cat#551459, dil: 1:200
6, Rabbit polyclonal anti-Cleaved Caspase3,Cell Signaling Technology, clone: Asp175, Cat#9661S, dil: 1:200
7, Rabbit monoclonal anti-Phospho-NF-kB P65, Cell Signaling Technology, clone: Ser536, Cat#3033S, dil: 1:200
8, Goat polyclonal anti-HNF-4a, Santa Cruz Biotechnology, clone: C-19, Cat#SC-6556, dil: 1:200
9,Rabbit polyclonal anti-ZO-1, Thermo Fisher Scientific, Cat#40-2200, dil: 1:200
10,Rabbit polyclonal anti-Collagen I, Bio-Rad, Cat#2150-1410, dil: 1:200
11,Goat polyclonal anti-rat IgG(H+L), Cy3 linked, Thermo Fisher Scientific,Cat#A10522, dil: 1:400
12,Goat polyclonal anti-rabbit IgG(H+L), Alexa Fluo 546 linked, Thermo Fisher Scientific, Cat#A-11010, dil: 1:400
13,Goat polyclonal anti-rabbit IgG(H+L), Alexa Fluo 488 linked, Thermo Fisher Scientific, Cat#A-11008, dil: 1:400
14,Donkey polyclonal anti-Goat IgG (H+L) Highly Cross-Adsorbed Secondary Antibody, Alexa Fluor™ Plus 647, Thermo Fisher Scientific, Cat#A32849, dil: 1:400
Western blot:
1, Rabbit polyclonal anti-IL-1 beta, Abcam, Cat#ab9722, dil: 1:1000
2, Rabbit monoclonal anti-pro Caspase-1 + p10 + p12, Abcam, clone: EPR16883, Cat#ab179515, dil: 1:800
3, Rabbit monoclonal anti-alpha smooth muscle Actin, Abcam, clone: E184, Cat#ab32575, dil: 1:800
4, Mouse monoclonal anti-FGF15, Santa Cruz Biotechnology, clone: D-9, Cat#sc-514647, dil: 1:500
5, Mouse monoclonal anti-CYP7A1, Santa Cruz Biotechnology, clone: E-10, Cat#sc-518007, dil: 1:500
6, Mouse monoclonal anti-NLRP3, AdipoGen, clone: Cryo-2, Cat#AG-20B-0014, dil: 1:1000
7,Rabbit monoclonal anti-cIAP1, Cell Signaling Technology, clone: E6R2S, Cat#70008S, dil: 1:1000
8,Rabbit monoclonal anti-A1/Bfl-1, Cell Signaling Technology, clone: E4P2I, Cat#64310S, dil: 1:1000
9,Mouse monoclonal anti-P-P65, Santa Cruz Biotechnology, clone: 27.Ser 536, Cat#SC-136548 HRP, dil: 1:500
10, Rabbit polyclonal anti-Occludin, Thermo Fisher Scientific, Cat#71-1500, dil: 1:1000
11, Mouse monoclonal anti-GAPDH, Bio-Rad, clone: 6C5, Cat# MCA4739, dil: 1:1000
12,Mouse monoclonal anti-β-Actin, Sigma-Aldrich, clone: AC-15, Cat#A5441, dil: 1:1000
13,Goat polyclonal anti-rabbit IgG, HRP linked, Cell Signaling Technology, Cat#7074, dil: 1:2000
14, Mouse monoclonal anti-mouse IgGκ light chain immunoglobulins, HRP linked, Santa Cruz Biotechnology, Cat# sc-516102, dil: 1:2000
FACS:
1, Rat monoclonal anti-CD45, APC-Cy7 conjugated, BD Biosciences, clone:30-F11, Cat#557659, dil: 1:84
2, Rat monoclonal anti-CD11b, V450 conjugated, BD Biosciences, clone: M1/70, Cat#560455, dil: 1:84
3, Rat monoclonal anti-F4/80, PE-Cy7 conjugated, Thermo Fisher Scientific, clone: BM8, Cat#25-4801-82, dil: 1:84
4,Rat monoclonal anti-Ly6C, PerCP-Cy5.5 conjugated, Thermo Fisher Scientific, clone: HK1.4, Cat#45-5932-82, dil: 1:84
5,Rat monoclonal anti-CD19, Alexa Fluor 700 conjugated, Thermo Fisher Scientific, clone: eBio1D3 (1D3), Cat#56-0193-82, dil: 1:84
6,Armenian Hamster monoclonal anti-CD3e, APC conjugated, Thermo Fisher Scientific, clone: 145-2C11, Cat# 17-0031-82, dil: 1:84
7,Rat monoclonal anti-CD4, eFluor 450 conjugated, Thermo Fisher Scientific, clone: GK1.5, Cat#48-0041-82, dil: 1:84
8,Rat monoclonal anti-CD8a, FITC conjugated, Thermo Fisher Scientific, clone: 53-6.7, Cat# 11-0081-85, dil: 1:84
9,Mouse monoclonal anti-NK1.1, PE-Cy7 conjugated, Thermo Fisher Scientific, clone: PK136, Cat#25-5941-82, dil: 1:84
10,Rat monoclonal anti-Ly6G, Alexa Fluor 700 conjugated, BioLegend,clone: 1A8, Cat#127622, dil: 1:84

11,Armenian Hamster monoclonal anti-CD11c, APC conjugated,BioLegend, clone: N418, Cat#117310, dil: 1:84
12,Rat monoclonal anti-I-A/I-E(MHC II), FITC conjugated, BioLegend,clone: M5/114.15.2, Cat#107605, dil: 1:84

Validation

IHC and IF stainings:
1,Rabbit monoclonal anti-MUC2, Abcam, Cat#ab272692, dil: 1:200. Liu Y et al. Mucus production stimulated by IFN-AhR signaling triggers hypoxia of COVID-19. Cell Res 30:1078-1087 (2020).
2,Rabbit monoclonal anti-Cytokeratin 19, Abcam, Cat#ab52625, dil: 1:200. Kasuga A et al. Oncogenic KRAS-expressing organoids with biliary epithelial stem cell properties give rise to biliary tract cancer in mice. Cancer Sci 112:1822-1838 (2021).
3, Rabbit monoclonal anti-Ki67, Cell Signaling Technology, Cat#12202, dil: 1:200. Zha JM et al. Interleukin 22 Expands Transit-Amplifying Cells While Depleting Lgr5+ Stem Cells via Inhibition of Wnt and Notch Signaling. Cell Mol Gastroenterol Hepatol. 2019;7 (2):255-274.
4, Rat monoclonal anti-CD11b, BD Biosciences, Cat#550282, dil: 1:200. Evelina Blomberg et al. Programmed death ligand 1 gene silencing in murine glioma models reveals cell line-specific modulation of tumor growth in vivo, Neuro-Oncology Advances, Volume 4, Issue 1, January-December 2022.
5, Rat monoclonal anti-Ly6G, BD Biosciences, Cat#551459, dil: 1:200. Que H et al.Tripterin liposome relieves severe acute respiratory syndrome as a potent COVID-19 treatment. Sig Transduct Target Ther 7, 399 (2022).
6, Rabbit polyclonal anti-Cleaved Caspase3,Cell Signaling Technology, Cat#9661S, dil: 1:200. Nakayama A et al. Suppression of CCL2 angiocrine function by adrenomedullin promotes tumor growth. J Exp Med. 2023 Jan 2;220(1):e20211628.
7, Rabbit monoclonal anti-Phospho-NF-kB P65, Cell Signaling Technology, Cat#3033S, dil: 1:200. Boonyarat C et al. Neuroprotective and anticancer effects of 7-Methoxyheptaphylline via the TAK1 pathway. Oncol Rep. 2023 Jan;49(1):15.
8, Goat polyclonal anti-HNF-4a, Santa Cruz Biotechnology, Cat#SC-6556, dil: 1:200. Gougelet A et al. Antitumour activity of an inhibitor of miR-34a in liver cancer with β-catenin-mutations. Gut. 2016 Jun;65(6):1024-34.
9,Rabbit polyclonal anti-ZO-1, Thermo Fisher Scientific, Cat#40-2200, dil: 1:200. Nishino T et al. Distinct distribution of the tensin family in the mouse kidney and small intestine. Exp Anim. 2012;61(5):525-32.
10,Rabbit polyclonal anti-Collagen I, Bio-Rad, Cat#2150-1410, dil: 1:200. Uezumi, A. et al. Fibrosis and adipogenesis originate from a common mesenchymal progenitor in skeletal muscle. J Cell Sci (2011). 124: 3654-64.
Western blot:
1, Rabbit polyclonal anti-IL-1 beta, Abcam, Cat#ab9722, dil: 1:1000. Pang N et al. Transcranial Ultrasound Stimulation of Hypothalamus in Aging Mice. IEEE Trans Ultrason Ferroelectr Freq Control 68:29-37 (2021).
2, Rabbit monoclonal anti-pro Caspase-1 + p10 + p12, Abcam, Cat#ab179515, dil: 1:800. Yu S et al. Bafilomycin A1 enhances NLRP3 inflammasome activation in human monocytes independent of lysosomal acidification. FEBS J 288:3186-3196 (2021).
3, Rabbit monoclonal anti-alpha smooth muscle Actin, Abcam, Cat#ab32575, dil: 1:800. Okawa ER et al. Essential roles of insulin and IGF-1 receptors during embryonic lineage development. Mol Metab 47:101164 (2021).
4, Mouse monoclonal anti-FGF15, Santa Cruz Biotechnology, Cat#sc-514647, dil: 1:500. Schneider KM, et al. Gut microbiota depletion exacerbates cholestatic liver injury via loss of FXR signalling. Nat Metab. 2021 Sep;3(9):1228-1241.
5, Mouse monoclonal anti-CYP7A1, Santa Cruz Biotechnology, Cat#sc-518007, dil: 1:500. Schneider KM, et al. Gut microbiota depletion exacerbates cholestatic liver injury via loss of FXR signalling. Nat Metab. 2021 Sep;3(9):1228-1241.
6, Mouse monoclonal anti-NLRP3, AdipoGen, Cat#AG-20B-0014, dil: 1:1000. Gutierrez KD, et al. MLKL Activation Triggers NLRP3-Mediated Processing and Release of IL-1β Independently of Gasdermin-D. J Immunol. 2017 Mar 1;198(5):2156-2164.
7,Rabbit monoclonal anti-cIAP1, Cell Signaling Technology, Cat#70008S, dil: 1:1000. https://www.cellsignal.de/products/primary-antibodies/c-iap1-e6r2s-rabbit-mab/70008?site-search-type=Products&N=4294956287&Ntt=70008s&fromPage=plp&_requestid=10352612
8,Rabbit monoclonal anti-A1/Bfl-1, Cell Signaling Technology, Cat#64310S, dil: 1:1000. Paik S, et al. Somatostatin Ameliorates β-Amyloid-Induced Cytotoxicity via the Regulation of CRMP2 Phosphorylation and Calcium Homeostasis in SH-SY5Y Cells. Biomedicines. 2021 Jan 2;9(1):27.
9,Mouse monoclonal anti-P-P65, Santa Cruz Biotechnology, Cat#SC-136548 HRP, dil: 1:500. Cardoso RDR, et al. Resolvin D5 (RvD5) Reduces Renal Damage Caused by LPS Endotoxemia in Female Mice. Molecules. 2022 Dec 23;28(1):121.
10, Rabbit polyclonal anti-Occludin, Thermo Fisher Scientific, Cat#71-1500, dil: 1:1000. Sayoc-Becerra A, et al. The JAK-Inhibitor Tofacitinib Rescues Human Intestinal Epithelial Cells and Colonoids from Cytokine-Induced Barrier Dysfunction. Inflamm Bowel Dis. 2020 Feb 11;26(3):407-422.
11, Mouse monoclonal anti-GAPDH, Bio-Rad, Cat# MCA4739, dil: 1:1000. Gautheron J, et al.The necroptosis-inducing kinase RIPK3 dampens adipose tissue inflammation and glucose intolerance. Nat Commun. 2016 Jun 21;7:11869.
12,Mouse monoclonal anti-β-Actin, Sigma-Aldrich, Cat#A5441, dil: 1:1000. Sreedharan R, et al. The maximal cytoprotective function of the heat shock protein 27 is dependent on heat shock protein 70. Biochim. Biophys. Acta Gen. Subj., 1813(1), 129-135 (2011).
FACS:
1, Rat monoclonal anti-CD45, APC-Cy7 conjugated, BD Biosciences, Cat#557659. Beavis AJ, Pennline KJ. Allo-7: a new fluorescent tandem dye for use in flow cytometry. Cytometry. 1996; 24(4):390-395.
2, Rat monoclonal anti-CD11b, V450 conjugated, BD Biosciences, Cat#560455. Ault KA, Springer TA. Cross-reaction of a rat-anti-mouse phagocyte-specific monoclonal antibody (anti-Mac-1) with human monocytes and natural killer cells. J Immunol. 1981; 126 (1):359-364.
3, Rat monoclonal anti-F4/80, PE-Cy7 conjugated, Thermo Fisher Scientific, Cat#25-4801-82. Gal-Oz ST, et al. ImmGen report: sexual dimorphism in the immune system transcriptome. Nat Commun. 2019 Sep 20;10(1):4295.
4,Rat monoclonal anti-Ly6C, PerCP-Cy5.5 conjugated, Thermo Fisher Scientific, Cat#45-5932-82. Dixit A, et al. Frontline Science: Proliferation of Ly6C+ monocytes during urinary tract infections is regulated by IL-6 trans-signaling. J Leukoc Biol. 2018 Jan;103 (1):13-22.
5,Rat monoclonal anti-CD19, Alexa Fluor 700 conjugated, Thermo Fisher Scientific, Cat#56-0193-82. Phadwal K, et al. A novel method for autophagy detection in primary cells: impaired levels of macroautophagy in immunosenescent T cells. Autophagy. 2012 Apr;8 (4):677-89.
6,Armenian Hamster monoclonal anti-CD3e, APC conjugated, Thermo Fisher Scientific, Cat# 17-0031-82. Hermann-Kleiter N, et al. The Nuclear Orphan Receptor NR2F6 Is a Central Checkpoint for Cancer Immune Surveillance. Cell Rep. 2015 Sep 29;12(12):2072-85.
7,Rat monoclonal anti-CD4, eFluor 450 conjugated, Thermo Fisher Scientific, Cat#48-0041-82. Chopp LB, et al. An Integrated Epigenomic and Transcriptomic Map of Mouse and Human αβ T Cell Development. Immunity. 2020 Dec 15;53(6):1182-1201.e8.

8,Rat monoclonal anti-CD8a, FITC conjugated, Thermo Fisher Scientific, Cat# 11-0081-85. Chentoufi AA, et al. The herpes simplex virus type 1 latency-associated transcript inhibits phenotypic and functional maturation of dendritic cells. Viral Immunol. 2012 Jun;25 (3):204-15.
9,Mouse monoclonal anti-NK1.1, PE-Cy7 conjugated, Thermo Fisher Scientific, Cat#25-5941-82. Fahl SP, et al. c-Myb is required for pro-B cell differentiation. J Immunol. 2009 Nov 1;183(9):5582-92. doi: 10.4049/jimmunol.0901187.
10,Rat monoclonal anti-Ly6G, Alexa Fluor 700 conjugated, BioLegend,Cat#127622.Daley JM, et al. Use of Ly6G-specific monoclonal antibody to deplete neutrophils in mice. J Leukoc Biol. 2008 Jan;83(1):64-70.
11,Armenian Hamster monoclonal anti-CD11c, APC conjugated,BioLegend, Cat#117310. De Riva A,et al. Accelerated turnover of MHC class II molecules in nonobese diabetic mice is developmentally and environmentally regulated in vivo and dispensable for autoimmunity. J Immunol. 2013 Jun 15;190(12):5961-71.
12,Rat monoclonal anti-I-A/I-E(MHC II), FITC conjugated, BioLegend,Cat#107605. Nakamura M, et al.A genome-wide analysis identifies a notch-RBP-Jκ-IL-7Rα axis that controls IL-17-producing γδ T cell homeostasis in mice. J Immunol. 2015 Jan 1;194 (1):243-51.

Animals and other research organisms

Policy information about studies involving animals; ARRIVE guidelines recommended for reporting animal research, and Sex and Gender in Research

Laboratory animals: Male WT and Mdr2-/- mice (FVB) as well as Mdr2-/-Nemof/f and Mdr2-/-NemoΔhepa mice (C57BL/6) were housed at a temperature of 21°-23°C with relative humidity of 35%-65% and under specific pathogen free conditions of 12h:12h light-dark cycle in individually ventilated cages with standard chow diet (ssniff #V1534-300) and water ad libitum. 8–10-week-old WT and Mdr2-/- (FVB/N) mice were used in the acute DSS, LPS and infliximab models. 12-week-old WT and Mdr2-/- (FVB/N) mice were used in the chronic DSS model. 9–14-week-old Mdr2-/-Nemof/f and Mdr2-/-NemoΔhepa mice (C57BL/6) were used.

Wild animals: The study did not include wild animals.

Reporting on sex: Only male mice were used in this study.

Field-collected samples: The study did not include field-collected samples.

Ethics oversight: All experimental procedures were performed according to the Guide for the Care and Use of Laboratory Animals and approved by the local authorities (LANUV, Germany; no. AZ-84-02.04. 2017.A327 (C.T.), no. AZ-81-02.04. 2020.A033(C.T.), no. AZ-81-02.04. 2022.A230(C.T.))

Note that full information on the approval of the study protocol must also be provided in the manuscript.

Flow Cytometry

Plots

Confirm that:

- [x] The axis labels state the marker and fluorochrome used (e.g. CD4-FITC).
- [x] The axis scales are clearly visible. Include numbers along axes only for bottom left plot of group (a 'group' is an analysis of identical markers).
- [x] All plots are contour plots with outliers or pseudocolor plots.
- [x] A numerical value for number of cells or percentage (with statistics) is provided.

Methodology

Sample preparation: Around 0.3 g fresh liver from the same lobe was chopped into small pieces and digested by collagenase type IV and DNase I at 37°C for 30 min. The mixture was then filtered through 70µm cell strainer, proceeded by multiple centrifugation steps as described (Karlmark, K.R. et al., Hepatology 2009) and removal of erythrocytes with RBC (Red blood cell) lysing buffer. Next, immune cells were divided into two panels and stained with corresponding subset of fluorochrome-conjugated antibodies: 1, myeloid panel: CD45::APC-Cy7, CD11b::V450, Ly6G::AF700, CD11c::APC, MHC II::FITC, F4/80::PE-Cy7, Ly6C::PerCP-Cy5.5, CD80::PE; 2, Lymphoid panel: CD45::APC-Cy7, CD3::APC, CD19::AF700, CD4:: eFluor 450, CD8::FITC, NK1.1::PE-Cy7, TIM3::BV650, CTLA4::PE, PD1:: PE/Dazzle 594, CD25::PERCP-Cy5.5. In the end, labeled cells were acquired on LSRFortessa (BD Biosciences). The data were analyzed by FlowJo software (version 10).

Instrument: labeled cells were acquired on LSRFortessa (BD Biosciences, Heidelberg, Germany).

Software: BD FACSDiva Software (BD Bioscience), FlowJo (Ashland) version 10

Cell population abundance: The relative abundance or absolute abundance of different cell populations were presented in the manuscript. No FACS-sorting was performed in this study.

Gating strategy: Myeloid cells:
1. Gating on cells according to FSC-SSC signals
2. Single cells were choosen based on FSC-H, FSC-A
Identification of cell populations was dependent on the expression of defined surface markers:

1. Leukocytes: CD45+ population
2. Neutrophils: CD45+/CD11b+/Ly6G+
3. Monocyte derived macrophages (MoMFs): CD45+/Ly6G-/CD11b+/F4/80low
4. Kupffer Cells: CD45+/Ly6G-/CD11b+/F4/80high

Lymphoid cells:
1. Gating on cells according to FSC-SSC signals
2. Single cells were choosen based on FSC-H, FSC-A

Identification of cell populations was dependent on the expression of defined surface markers:
1. Leukocytes: CD45+ population
2. NK cells: CD45+/CD3-/NK1.1+
3. NKT cells: CD45+/CD3+/NK1.1+
4. B cells: CD45+/CD19+/CD3-
5. T cells: CD45+/CD19-/CD3+
6. CD8+ T cells: CD45+/CD19-/CD3+/CD8+/CD4-
7. CD4+ T cells: CD45+/CD19-/CD3+/CD4+/CD8-

☒ Tick this box to confirm that a figure exemplifying the gating strategy is provided in the Supplementary Information.
